# Large geographic distance *versus* small DNA barcode divergence: Insights from a comparison of European to South Siberian Lepidoptera

**DOI:** 10.1371/journal.pone.0206668

**Published:** 2018-11-02

**Authors:** Peter Huemer, Paul D. N. Hebert, Marko Mutanen, Christian Wieser, Benjamin Wiesmair, Axel Hausmann, Roman Yakovlev, Markus Möst, Brigitte Gottsberger, Patrick Strutzenberger, Konrad Fiedler

**Affiliations:** 1 Naturwissenschaftliche Sammlungen, Tiroler Landesmuseen Betriebsges.m.b.H., Innsbruck, Austria; 2 Centre for Biodiversity Genomics, University of Guelph, Guelph, Canada; 3 Ecology and Genetics Research Unit, University of Oulu, Oulu, Finland; 4 Landesmuseum Kärnten, Klagenfurt, Austria; 5 Section Lepidoptera, Bavarian State Collection of Zoology, Munich, Germany; 6 Ecology Department, Altai State University, Barnaul, Russia; 7 Tomsk State University, Tomsk, Russia; 8 Department of Ecology, University of Innsbruck, Innsbruck, Austria; 9 Department of Botany & Biodiversity Research, University of Vienna, Vienna, Austria; Tierarztliche Hochschule Hannover, GERMANY

## Abstract

Spanning nearly 13,000 km, the Palearctic region provides an opportunity to examine the level of geographic coverage required for a DNA barcode reference library to be effective in identifying species with broad ranges. This study examines barcode divergences between populations of 102 species of Lepidoptera from Europe and South Siberia, sites roughly 6,000 km apart. While three-quarters of these species showed divergence between their Asian and European populations, these divergence values ranged between 0–1%, distinctly less than the distance to the Nearest-Neighbor species in all but a few cases. Our results suggest that further taxonomic studies may be required for 16 species that showed either extremely low interspecific or high intraspecific variation. For example, seven species pairs showed low or no barcode divergence, but four of these cases are likely to reflect taxonomic over-splitting while the others involve species pairs that are either young or show evidence for introgression. Conversely, some of the nine species with deep intraspecific divergence at varied spatial levels may include overlooked species. Although these 16 cases require further investigation, our overall results indicate that barcode reference libraries based on records from one locality can be very effective in identifying specimens across an extensive geographic area.

## Introduction

In many cases, DNA barcoding can be an effective tool for both specimen identification and species discovery. In animals, a 648 base pair segment of the mitochondrial cytochrome *c* oxidase subunit 1 (COI) gene has been adopted as the barcode region [1], [2]. Numerous researchers have added data to BOLD, the Barcode of Life Data Systems (www.boldsystems.org), which at present includes more than 6 million barcode records from about 550,000 operational taxonomic units (i.e. BINs–see [3]). Currently, more than 22,000 registered users are accessing these records. Despite varied coverage among taxonomic groups and regions, these data are increasingly useful to address diverse research questions in ecology and evolutionary biology.

One important issue that needs further investigation relates to the performance of barcode-based species identifications across large distances. In particular, since species’ distributions vary from narrow endemism to global occurrence, it needs to be assessed whether DNA barcodes from one site or region can be used to identify specimens of the same species from distant localities. This is especially important in the Palearctic region because its elongate axis spans more than 13,000 km and many species are thought to occur from the Atlantic Ocean in the west to the Pacific Ocean in the east. For the same reason, the Palearctic region is ideal to quantify the influence of geographic distance on intraspecific variation under relatively comparable conditions (similar ecotypes). In recent years, a few studies on Lepidoptera have examined the congruence of DNA barcodes across larger geographic distances including 1,000 species shared by Fennoscandia and Central Europe [4], butterflies from Central Asia [5], and 1,500 species of Noctuoidea in North America [6]. However, these studies still are the exception and in contrast to the present paper either only cover a single taxonomic sub-group of Lepidoptera or a comparatively small geographic distance. Moreover, most prior work has examined patterns of sequence variation at a national or regional level [7], [8], [9].

Ideally, DNA barcodes from specimens collected at a single locality would enable the identification of conspecifics from the entire species distribution. This might not be the case if intraspecific sequence variation within widespread taxa is greater than interspecific differences. In other words, identification problems will arise whenever intraspecific variation blurs the ‘barcode gap’ which is critical to assign specimens to their correct species, either a Linnaean name or an Operational Taxonomic Unit (e.g. BIN). In such cases DNA barcodes fail to correctly identify species and additional diagnostic characters, particularly morphological traits and high density genetic markers, have to be considered to firmly identify species.

Our study is the first to examine patterns of DNA barcode variation across a very large geographic range for a broad set of Lepidoptera (102 species, 22 families) shared by Europe and South Siberia. Specifically, we ascertain levels of barcode divergence between putatively conspecific specimens from southern Siberia, i.e. Russian Altai, and Europe, particularly Northern Europe and the Alps. Although higher intraspecific variation within populations spanning Siberia and Europe compared to the respective populations from each region considered separately can be expected, the magnitude of this variation will determine whether an effective system for DNA barcode-based identifications can be based on a narrowly parameterized reference library. To examine this matter, we compared intraspecific divergences between populations of 102 species from Siberia and their divergences to the 5,016 species (41,583 specimens) in a carefully validated dataset of European Lepidoptera [10]. We also ascertained if intraspecific distances are lower in species with a near-continuous Euro-Siberian distribution than in those with a disjunct arctic-alpine or central-Asian-alpine distribution. Finally, we asked if patterns of isolation by geographic distance as measured by COI barcode sequences are influenced by overall sequence divergence or distribution type.

## Material and methods

### Taxon sampling strategy

**Fig 1:**
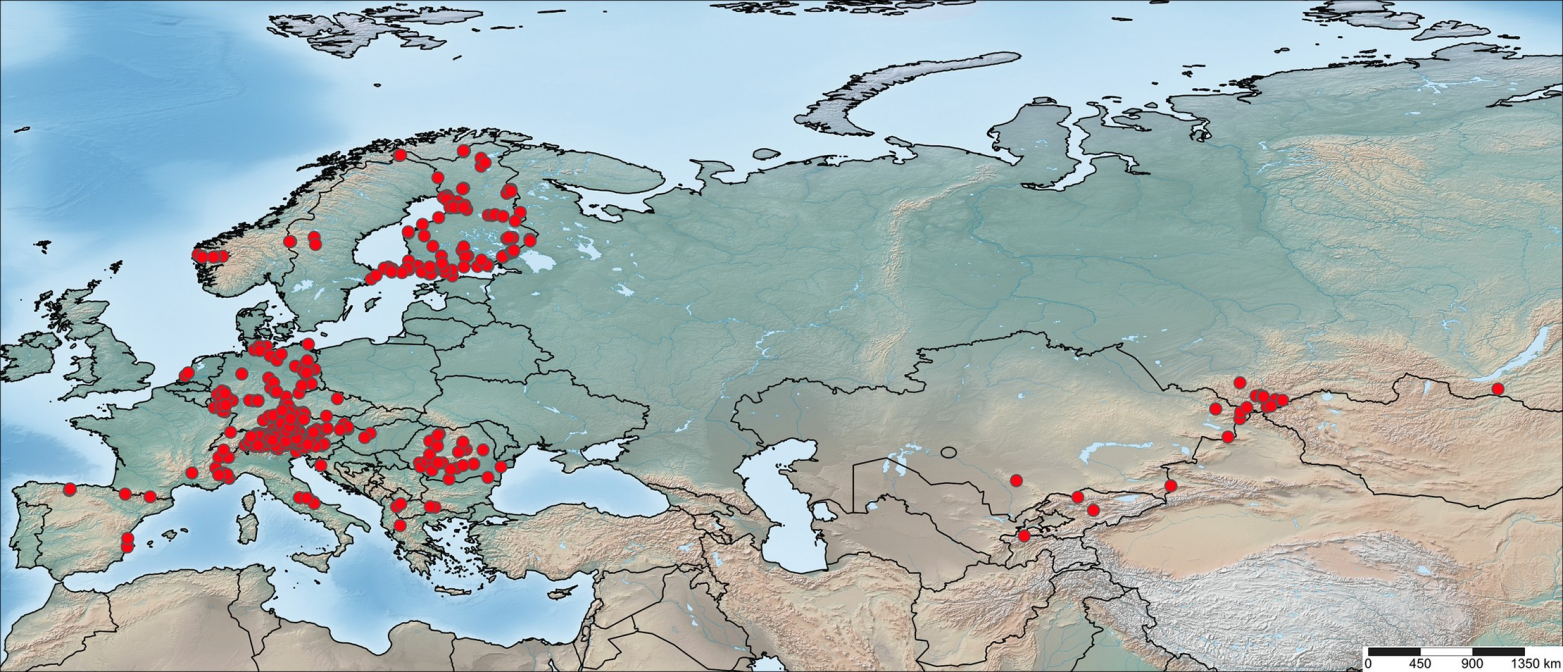
Geographic origin of the voucher specimens for the 102 sequenced species of Eurasian Lepidoptera. Map created with SimpleMappr (http://www.simplemappr.net).

This study examined two Palaearctic sub-regions separated by a distance of about 6,000 km: central/northern Europe with a focus on the Alps and Finland, and South Siberia (Altai Republic, Russia), supplemented by a few reliably identified specimens from other areas (Fig 1).

Whereas DNA barcode coverage for lepidopteran taxa is generally high for species from central and northern Europe, only few records are available from South Siberia. We therefore sought to obtain specimens of >100 species shared by these regions. We focused on species with a disjunct arctic-alpine and South Siberian-alpine distribution based on the expectation that they would be likely to show higher intraspecific barcode variation.

Species identification was exclusively based on morphological traits.

In general, we analyzed three specimens from South Siberia for each of these species to estimate intraspecific divergence, but only two specimens were available for 18 species whereas for 15 species the number of voucher specimens ranged between 4 and 8. The average number of successfully sequenced specimens per species from Asia was 3.24. By comparison, the number of sequenced specimens was much higher for most European representatives of these species with 16.34 sequenced specimens per species on average. Existing specimens from museum collections were analyzed where possible and were supplemented with material from an expedition to the Russian Altai Mountains from late July to mid-August 2016 [11]. A permit was not required for the Altai specimens as no protected species were collected. Collections in other countries were made in compliance with current legislation. In Finland, permits were issued by the Finnish Centre for Economic Development, Transport, and the Environment to MM under permissions VARELY/441/07.01/2012 and LAPELY/275/07.01/2012, while collecting permits were not necessary for scientific research in Austria/Tyrol. The Nagoya protocol was not applicable because our European material was collected before October 12, 2014 and because the protocol has not been ratified by Russia.

Most sequences considered in this study derive from specimens held in the Tiroler Landesmuseum Ferdinandeum, Innsbruck, Austria; the University of Oulu, Oulu, Finland; the Bavarian State Collection of Zoology, Munich, Germany; and another 25 specimen depositories. Wherever possible, data were supplemented by publicly available sequences in BOLD ([12], see http://www.boldsystems.org).

### DNA sequencing

For freshly collected specimens, a single leg was removed and placed in a 96-well lysis plate that was submitted for analysis to the CCDB (Canadian Center for DNA Barcoding, University of Guelph, Canada) where DNA extraction, PCR amplification, and sequencing were performed following standard high-throughput protocols [13].

Altogether, 315 specimens of 102 South Siberian species that also occur in Europe were sequenced. Moreover, we examined previously published 1,682 sequences (>500bp) [10] from specimens of the same species from sites in Europe including Finland (423), Austria (410), Germany (329), Russia (315), and 19 other countries (520) (Fig 1). Information regarding the institutions hosting each publicly available specimen, sample and process IDs and GenBank accession numbers are available in S1 Table. Further details on each specimen, including complete voucher data, and images are available on BOLD [12] in the public dataset “Lepidoptera of Altai Mountains (DS-LEPEUALT)” under the DOI: 10.5883/DS-LEPEUALT.

### Data analysis

The extent of intraspecific sequence variation in the COI sequences for each species was estimated using the Kimura-2-parameter (K2P) model of nucleotide substitution using analytical tools on BOLD v4.0 (http://www.boldsystems.org) and MEGA v.6 [14]. There has been an interesting debate over the choice and justification of K2P and other distance measures used in barcoding analyses (e.g., [15]), however, the ‘best method’ depends on the dataset under consideration and the effects of different distance measures and models on the distances and identification success are generally small (e.g., [16]). Therefore a consequence of model choice on the main results of this specific work is unlikely and we applied the K2P method as implemented in BOLD. For each species we obtained four estimates of intraspecific divergence by calculating the arithmetic mean for all pairwise distances (K2P) among conspecific individuals within the following spatial contexts: (a) ‘total intraspecific’ (mean distance for all data for each species); (b) ‘within Europe’ (mean distance for all European samples); (c) ‘within Asia’ (mean distance for all South Siberian-Central Asian samples); and (d) ‘inter Europe-Asia’ (mean distance within each species for all pairs of specimens from Europe vs. Asia).

Furthermore, we examined the potential impact of distribution type on intraspecific divergences. For this analysis, each species was assigned to one of two categories: (a) those with largely continuous distributions across Eurasia, i.e. with known gaps <500 km; and (b) those with highly disjunct distributions, i.e. with gaps between known populations >2,000 km. These two categories basically reflect what has been termed Euro-Siberian *versus* arctic-alpine and South Siberian-alpine distribution patterns in biogeographic studies [17].

We compared mean intraspecific sequence divergences across the three spatial levels (intra-Europe, intra-Asia, inter-Europe-Asia) using a non-parametric Friedman ANOVA of ranks because of uneven variance and sequence numbers for the 102 species. Total mean intraspecific barcode divergence between the two types of species distributions was compared using a Mann-Whitney U-test. In addition, we examined the strength of isolation by distance within every species. For this purpose, we calculated a Mantel correlation coefficient for the matrix of geographic distances between sampling localities and the K2P distance matrix for every species using the Geographic Distance Correlation tool in BOLD. These correlation coefficients were then tested for contingency upon distribution type or overall intraspecific sequence divergence using a Mann-Whitney test and a Spearman rank correlation, respectively. Statistical analyses were performed using Statistica 8.0 (StatSoft Inc.).

Finally, we compared the mean and maximum intraspecific divergence for each of the 102 species with its Nearest-Neighbor (NN) distance, because a gap between intraspecific and interspecific variation is essential for DNA barcoding to be effective in specimen identification. For this purpose we used the DS-MARKALL dataset (dx.doi.org/10.5883/DS-MARKALL). It includes >500 bp sequence records for 41,583 specimens representing 5,016 species of Lepidoptera [10]. We limited comparisons to this dataset because it is both comprehensive and identifications are very reliable. Sequences from the present study and from DS-MARKALL were pooled, and a barcode gap analysis was then carried out on BOLD using the K2P model. This analysis estimated the minimum genetic divergence to the NN and both the mean and maximum intraspecific divergences for each species.

## Results

### Sequenced species

We collected 1,997 sequences >500 bp from the 102 species. Among them, 54 sequences were not barcode compliant according to the standards in BOLD, i.e. a minimum sequence length of 500 bp, less than 1% ambiguous bases, the presence of two trace files, a minimum of low trace quality status, and the presence of a country specification in the record as set out by the Consortium for DNA Barcoding (CBOL), most likely due to partially degraded DNA. Nevertheless, these 54 sequences were still considered in the analysis as they were correctly placed with their conspecifics in an initial NJ tree. The seven families with the largest numbers of sequences were Noctuidae (551), Geometridae (389), Erebidae (175), Tortricidae (157), Nymphalidae (146), Gelechiidae (144), and Lycaenidae (133).

### Intraspecific barcode divergences

**Fig 2:**
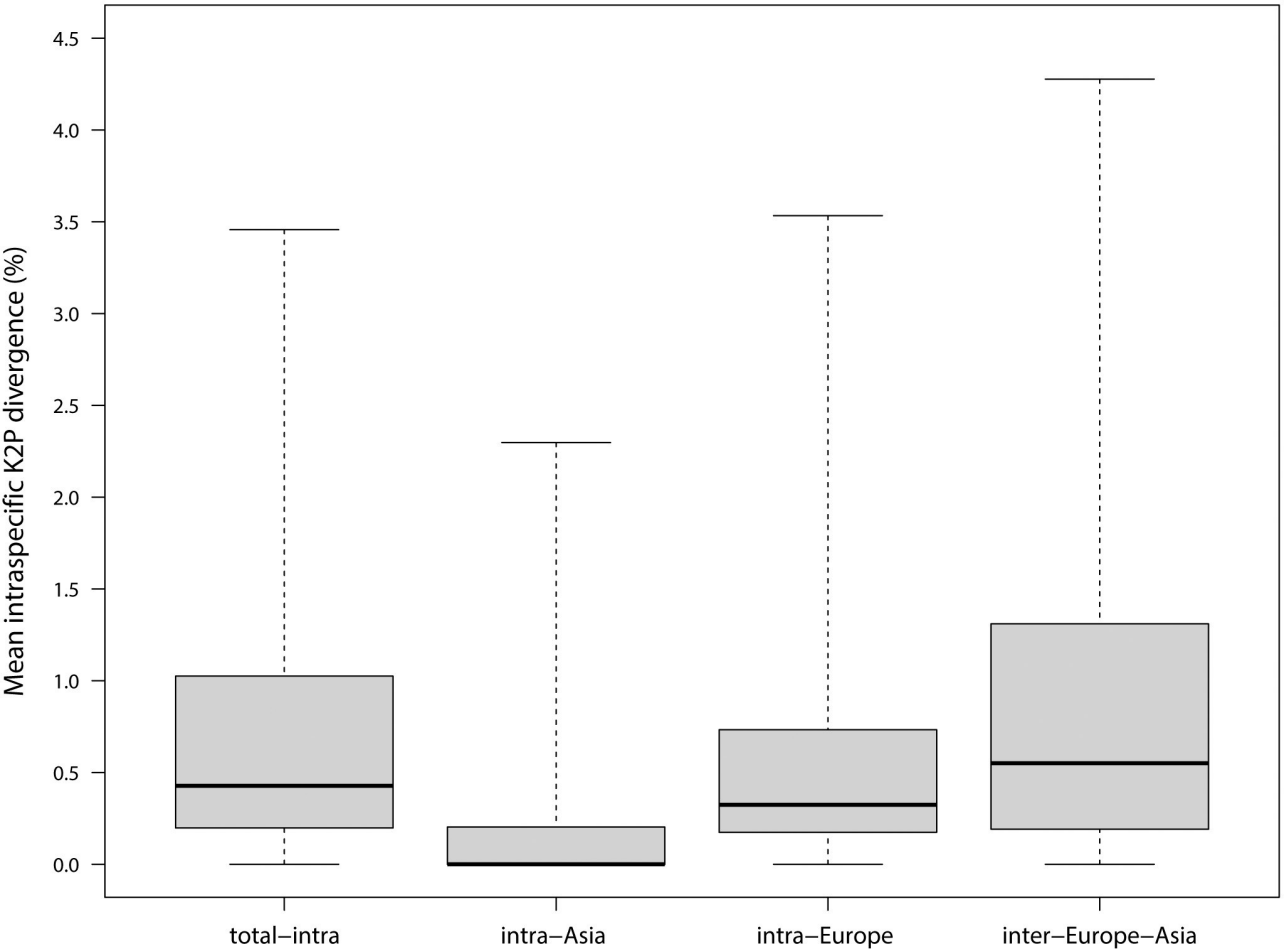
Mean intraspecific sequence divergences for 102 Lepidoptera species in geographic comparisons. Boxplots (median, interquartile range, total range) of mean intraspecific sequence divergences (Kimura-2-Parameter) for 102 Lepidoptera species: total intraspecific divergences (mean distance for all data for each species), and intraspecific divergences at three geographic levels: intra-Asia (mean distance for all South Siberian samples); intra-Europe (mean distance for all European samples); inter- Europe-Asia (mean distance within each species for all pairs of specimens from Europe vs. Asia).

Intraspecific barcode divergence was generally <1% with a mean (± SD) of 0.68 ± 0.67% (median: 0.43%; range: 0.00 to 3.46%) for the 102 species. As expected, there were highly significant differences among the three regional comparisons (Friedman ANOVA: χ^2^_2df_ = 77.82; *p*<0.0001). Divergences were lowest within the Asiatic samples as expected because they originated from few collecting sites with low numbers of specimens, while divergences within Europe averaged higher, and those between the European and Asiatic samples were highest (Fig 2, Table 1). In post-hoc comparisons, all three pairwise comparisons were highly significant (Wilcoxon-tests, *p*<0.007).

**Table 1:** Mean intraspecific barcode divergences (% Kimura-2P-distances) for 102 Lepidoptera species from Europe and South Siberia and for the geographic comparisons, and distribution type.

Species	total-intra	intra-Asia	intra-Europe	inter-Europe-Asia	distribution type
*Acleris aspersana*	0.23	0.20	0.25	0.22	continuous
*Acompsia cinerella*	0.86	0.00	0.94	0.79	continuous
*Acronicta auricoma*	0.09	0.15*	0.08	0.12	continuous
*Aethes kindermanniana*	1.08	0.46*	0.85	1.41	continuous
*Agrotis fatidica*	0.18	0.00	0.17	0.23	disjunct
*Anaplectoides prasina*	0.03	0.15*	1.63	0.89	continuous
*Apamea furva*	0.41	0.20	0.34	0.54	continuous
*Apamea lateritia*	0.11	0.00	0.13	0.07	continuous
*Arctia caja*	0.65	1.56	0.39	1.51	continuous
*Arctia flavia*	0.21	0.00	0.19	0.26	disjunct
*Argyresthia pygmaeella*	0.28	0.10	0.30	0.25	continuous
*Arichanna melanaria*	0.12	0.00	0.07	0.19	continuous
*Athrips pruinosella*	1.43	0.00	1.51	1.63	continuous
*Autographa pulchrina*	0.17	0.00	0.17	0.10	continuous
*Boloria dia*	0.32	0.21	0.12	0.62	continuous
*Boloria napaea*	1.00	0.79	0.28	1.54	disjunct
*Boloria titania*	0.74	0.07	0.20	1.90	disjunct
*Brenthis ino*	0.47	0.22	0.53	0.42	continuous
*Carsia sororiata*	0.54	0.00	0.53	0.60	disjunct
*Caryocolum leucomelanella*	1.09	2.06	0.53	1.89	continuous
*Caryocolum pullatella*	2.02	2.30*	2.02	2.10	disjunct
*Catoptria languidellus*	0.98	0.10	0.52	1.52	disjunct
*Celypha rivulana*	0.54	0.46*	0.58	0.48	continuous
*Cerapteryx graminis*	0.39	0.41	0.36	0.47	continuous
*Charissa ambiguata*	0.86	0.31*	0.60	1.59	continuous
*Chionodes distinctella*	1.35	1.11	1.38	1.27	continuous
*Chionodes holosericella*	0.26	0.00	0.19	0.41	disjunct
*Coenonympha glycerion*	0.32	0.00	0.33	0.32	continuous
*Coenonympha tullia*	0.33	0.18	0.22	0.51	continuous
*Colostygia aptata*	0.43	0.15*	0.43	0.43	disjunct
*Coscinia cribraria*	3.46	0.95	3.53	3.16	continuous
*Crambus perlella*	0.39	0.38	0.27	0.57	continuous
*Crocallis elinguaria*	1.38	0.20	1.49	0.92	continuous
*Cupido minimus*	0.23	0.11	0.24	0.21	continuous
*Cyaniris semiargus*	0.31	0.40	0.29	0.34	continuous
*Diarsia brunnea*	0.20	0.00	0.21	0.15	continuous
*Diarsia mendica*	1.86	0.00	2.00	1.38	continuous
*Dicallomera fascelina*	1.50	0.51	0.22	3.53	continuous
*Eana osseana*	1.83	0.10	0.39	3.90	continuous
*Eana penziana*	0.33	0.00	0.32	0.35	continuous
*Eilema lutarella*	0.13	0.00*	0.15	0.08	continuous
*Elachista bedellella*	1.03	0.65	0.87	1.31	continuous
*Entephria caesiata*	0.70	0.00	0.65	0.94	continuous
*Epermenia illigerella*	0.73	0.00	0.83	0.60	continuous
*Epinotia cruciana*	0.92	0.00	1.12	0.67	continuous
*Epinotia trigonella*	1.05	1.13	1.06	0.10	continuous
*Eudonia alpina*	0.06	0.00*	0.10	0.05	continuous
*Eulamprotes wilkella*	1.87	0.10	1.97	1.71	continuous
*Eulithis populata*	0.25	0.00*	0.27	0.19	continuous
*Eulithis prunata*	2.47	0.00	2.02	4.28	continuous
*Eulithis testata*	0.37	0.00*	0.19	0.62	continuous
*Eumedonia eumedon*	1.08	1.53	0.74	1.69	continuous
*Euphyia unangulata*	0.16	0.15*	0.15	0.19	continuous
*Eupithecia pusillata*	0.20	0.00*	0.22	0.10	continuous
*Eurois occulta*	0.06	0.00	0.07	0.04	continuous
*Euxoa recussa*	0.06	0.00	0.08	0.04	continuous
*Gazoryctra ganna*	2.16	1.39*	1.08	3.58	disjunct
*Graphiphora augur*	0.10	0.00	0.12	0.06	continuous
*Gypsonoma nitidulana*	1.35	0.00*	1.16	2.12	continuous
*Hadena compta*	0.16	0.00	0.19	0.10	continuous
*Lasionycta imbecilla*	0.54	0.10	0.54	0.58	continuous
*Lasionycta proxima*	0.72	0.48	0.57	1.02	continuous
*Levipalpus hepatariella*	0.45	0.00	0.52	0.44	disjunct
*Lycaena virgaureae*	0.21	0.00	0.23	0.13	continuous
*Macaria brunneata*	0.47	0.00	0.46	0.57	continuous
*Matilella fusca*	0.24	0.00*	0.28	0.17	continuous
*Miltochrista miniata*	0.00	0.00*	0.00	0.00	continuous
*Mompha locupletella*	0.08	0.00	0.10	0.05	continuous
*Monopis spilotella*	1.03	0.51	0.61	1.33	continuous
*Noctua interposita*	0.06	0.00	0.07	0.04	continuous
*Ochsenheimeria urella*	2.10	0.15	2.10	2.54	continuous
*Oidaematophorus rogenhoferi*	0.51	0.31	0.46	0.61	disjunct
*Papestra biren*	0.09	0.00*	0.11	0.06	continuous
*Parnassius phoebus*	0.49	0.69	0.24	0.57	disjunct
*Pediasia aridella*	0.46	0.00*	0.51	0.42	continuous
*Perizoma hydrata*	0.00	0.00	0.00	0.00	continuous
*Phiaris obsoletana*	0.09	0.10	0.08	0.09	continuous
*Plebejus orbitulus*	0.43	0.10	0.08	0.82	disjunct
*Polia bombycina*	0.07	0.00*	0.07	0.04	continuous
*Polypogon tentacularia*	0.23	0.00	0.23	0.25	continuous
*Pontia callidice*	1.81	0.10	0.23	3.42	disjunct
*Protolampra sobrina*	0.03	0.10	0.00	0.05	continuous
*Pyrausta aerealis*	0.99	0.00	1.13	0.82	continuous
*Scopula incanata*	1.15	0.10	1.24	0.94	continuous
*Scopula virgulata*	0.25	0.00	0.31	0.20	continuous
*Scotopteryx chenopodiata*	0.15	0.00*	0.17	0.09	continuous
*Scrobipalpula diffluella*	2.06	0.18	0.51	3.55	disjunct
*Selagia spadicella*	0.82	0.00	0.55	1.21	continuous
*Setina irrorella*	2.04	0.00	2.22	1.95	continuous
*Sparganothis pilleriana*	0.40	0.20	0.47	0.37	continuous
*Syngrapha ain*	0.11	0.06*	0.03	0.20	continuous
*Syngrapha hochenwarthi*	0.31	0.00	0.21	0.59	disjunct
*Syngrapha interrogationis*	0.07	0.00*	0.06	0.09	continuous
*Trichiura crataegi*	0.82	0.20	0.73	1.21	continuous
*Udea uliginosalis*	1.76	0.00	1.65	2.20	disjunct
*Xanthorhoe decoloraria*	0.59	0.00	0.60	0.60	disjunct
*Xanthorhoe montanata*	1.39	0.00	1.53	0.79	continuous
*Xestia speciosa*	1.44	0.93	1.33	1.91	continuous
*Yponomeuta evonymella*	0.22	0.46*	0.15	0.41	continuous
*Ypsolopha dentella*	0.34	0.00	0.23	0.49	continuous
*Ypsolopha nemorella*	0.54	0.00	0.62	0.50	continuous
*Zeiraphera griseana*	0.05	0.00	0.06	0.03	continuous
**Mean Values**	**0.55**	**0.21**	**0.47**	**0.72**	

Species with an asterisk (*) indicate intraspecific variation assessed from 2 specimens.

### Relationship between distribution type and intraspecific barcode divergences

**Fig 3:**
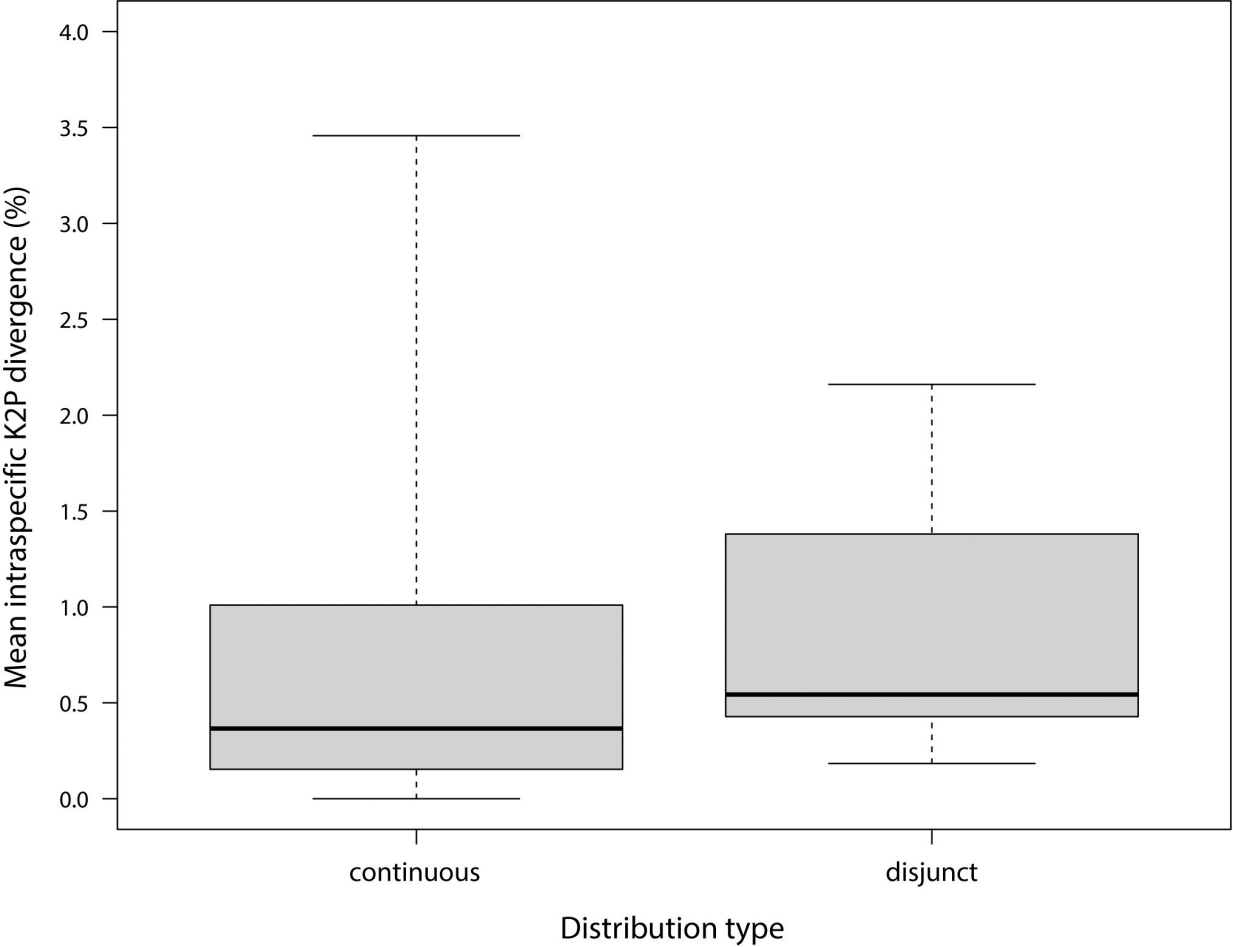
Mean intraspecific sequence divergences for 102 Lepidoptera species in different distribution types. Boxplot (median, interquartile range, total range) of total mean intraspecific sequence divergences (Kimura-2-Parameter) for 102 Lepidoptera species from Europe and South Siberia, comparing species with continuous versus disjunct distributions.

Contrary to expectation, total intraspecific divergence values were only slightly larger in species with disjunct as opposed to those with continuous distributions (Mann-Whitney test: *z* = 2.09; *p* = 0.036; Fig 3). Species with continuous ranges (n = 83) had an average intraspecific sequence divergence of 0.63± 0.66% (median: 0.37%; range = 0.00–3.46%), while those with disjunct distributions (n = 19) showed a divergence of 0.89± 0.70% (median: 0.54%; range = 0.18–2.16%).

### Factors affecting isolation by distance within species

**Fig 4:**
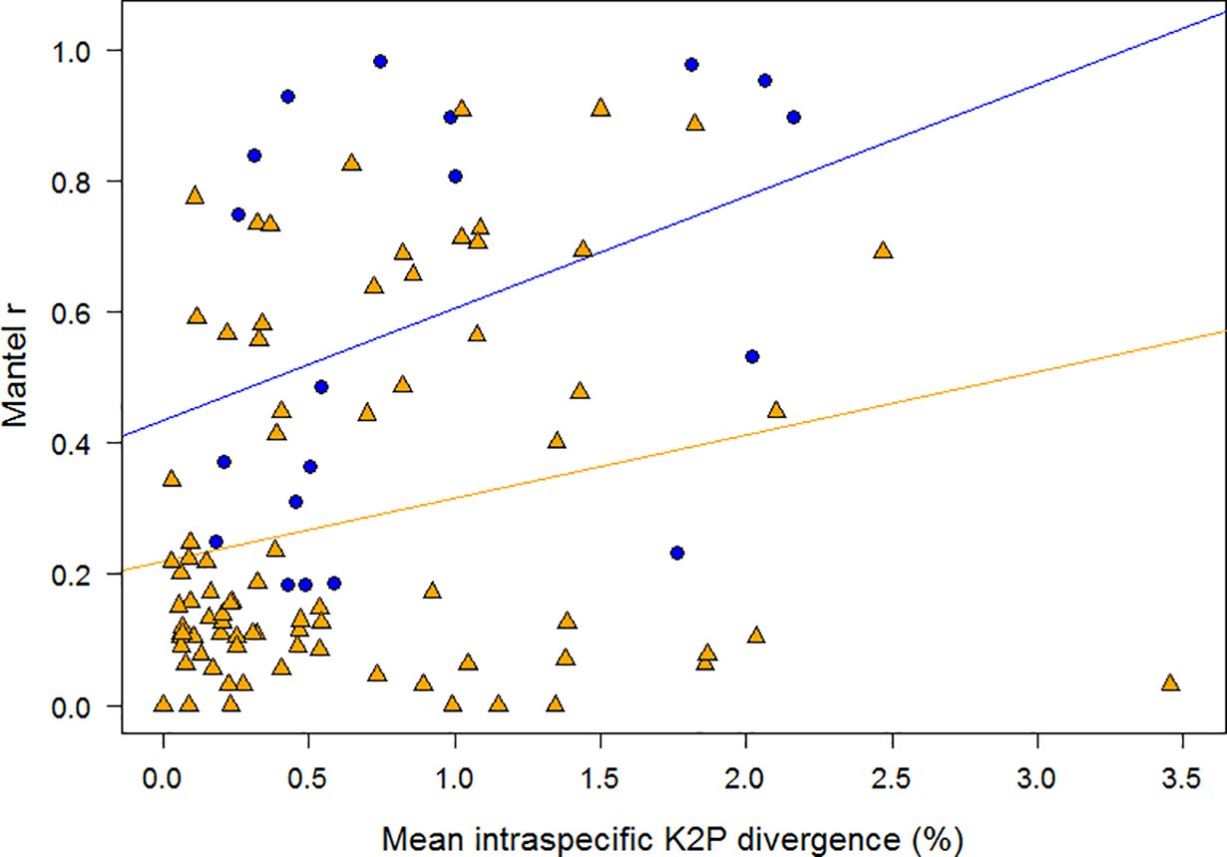
Relationship of intraspecific sequence divergence and geographic distance. Relationship between mean overall intraspecific sequence divergence and the extent of isolation by distance (as quantified by the Mantel correlation coefficient, *r*), with species patitioned according to their type of distribution. Species with disjunct distributions (blue circles) tended to show stronger isolation-by-distance (i.e. higher *r* values) than species with continuous distributions (orange triangles), and this pattern was marginally stronger in species with higher overall levels of intraspecific sequence divergence.

As expected, the extent of sequence divergence between members of a species was often related to the distance between their sites of collection. However, the extent of this isolation-by-distance effect was highly variable among species. Sequence divergences in 56 of the 102 species showed no association with distance, while 13 species showed a weakly significant Mantel correlation (*p*<0.05) and 33 species showed a strong relationship (*p*<0.01). Evidence for isolation-by-distance was stronger in species with disjunct (mean Mantel *r* = 0.59±0.32) than continuous distributions (mean Mantel *r* = 0.28±0.27; Mann-Whitney test: *z* = 4.19, *p*<0.0001; Fig 4). In species with disjunct distributions, the extent of isolation-by-distance was only weakly and non-significantly related to overall sequence divergence (Spearman rank correlation: *r*_S_ = 0.40, *p* = 0.087), and this relationship was even weaker and also non-significant for species with continuous ranges (*r*_S_ = 0.20; *p* = 0.073). The strength of isolation-by-distance patterns within species did not co-vary with the maximum distance between sampling sites (*r*_S_ = -0.005, *p* = 0.96), but it was negatively related to the number of sequences available for a taxon (*r*_S_ = -0.27, *p* = 0.007).

### Relationships between interspecific and intraspecific divergences

**Fig 5:**
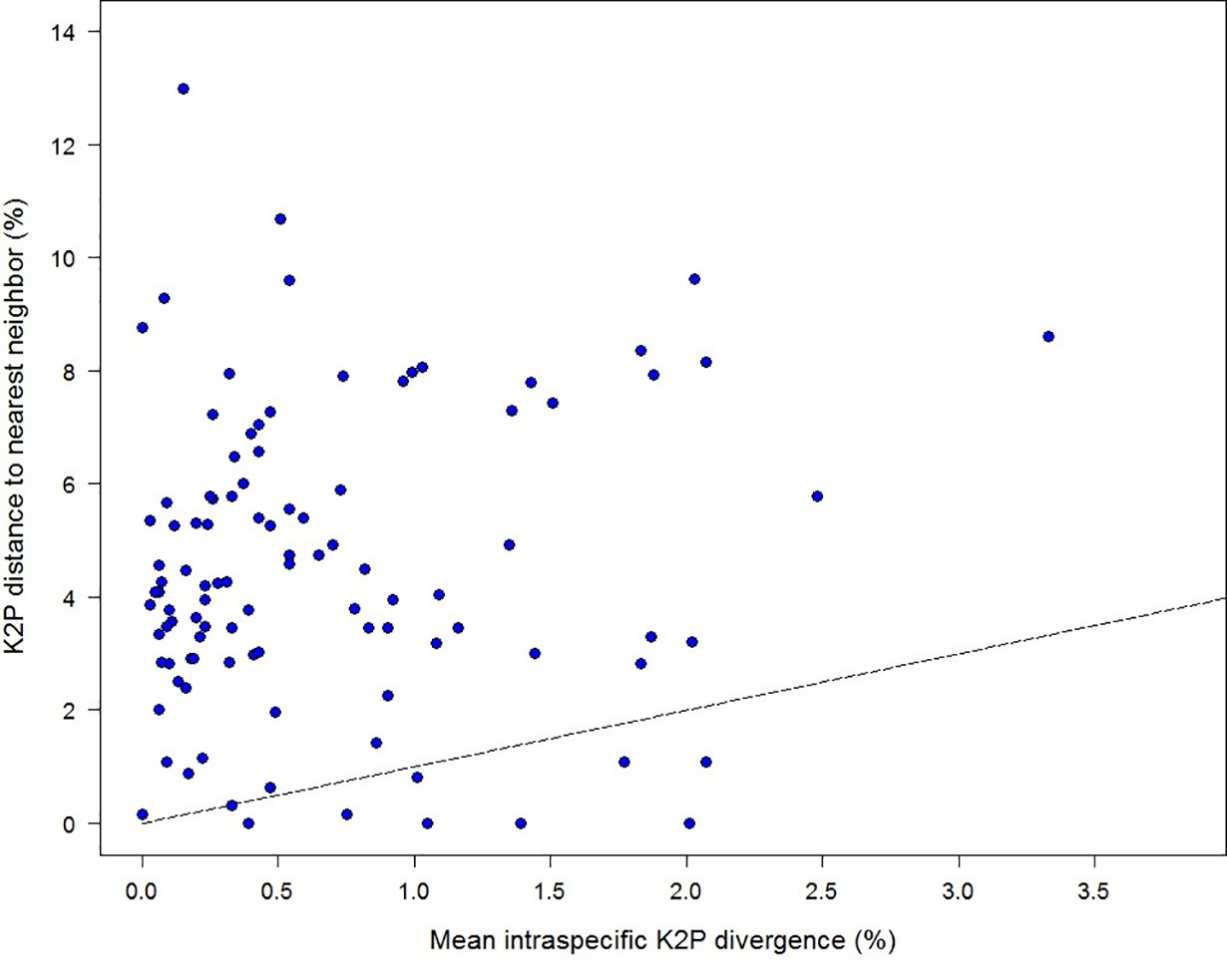
Mean intraspecific sequence divergences for 102 Lepidoptera species in relation to nearest neighbor. Barcode sequence distances to the nearest neighbor species in relation to mean intraspecific distances for 102 species of Palearctic Lepidoptera. The straight line indicates where distance to nearest neighbor equals the respective intraspecific distance, viz. for species above this line the ‘barcode gap’ does exist.

**Fig 6:**
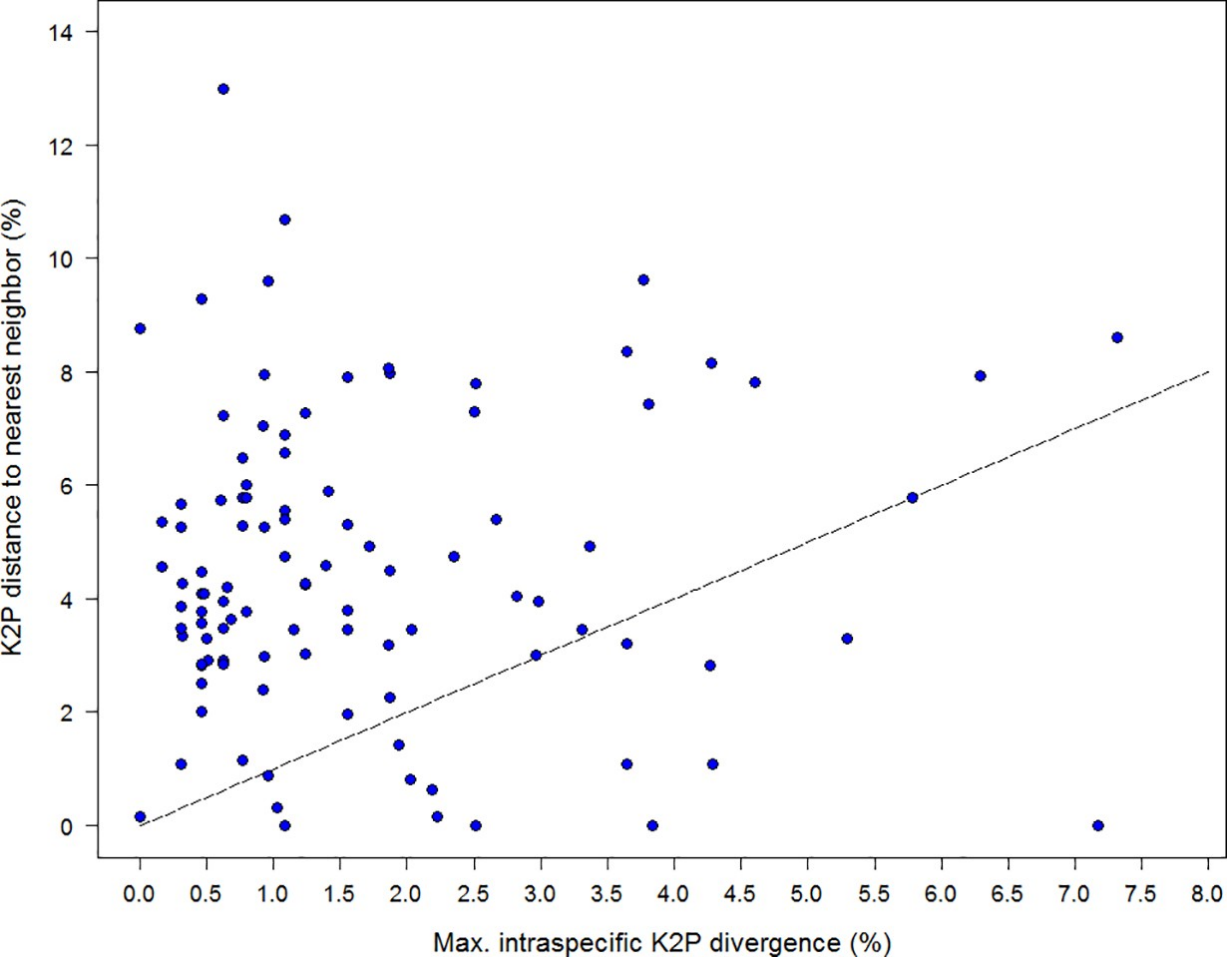
Maximum intraspecific sequence divergences for 102 Lepidoptera species in relation to nearest neighbor. Barcode sequence distances to the nearest neighbor species in relation to maximum intraspecific distances for 102 species of Palearctic Lepidoptera. The straight line indicates where distance to nearest neighbor equals the respective intraspecific distance, viz. for species above this line the ‘barcode gap’ does exist.

Nearest Neighbor distances (K2P) for the 102 species averaged 4.52%, but ranged from 0.00–12.98%. By comparison, maximum intraspecific divergence values averaged 1.69% (range = 0.00–7.32%) while mean intraspecific variation values averaged 0.68% (range = 0.00–3.46%). Therefore, the gap to the NN species averaged 2.73-fold the maximum intraspecific variation (Wilcoxon test: *z* = 7.22, *p*<0.0001), and 6.90-fold the mean intraspecific variation (*z* = 8.47, *p*<0.0001). While the barcode gap was clear in most cases, divergence to the NN was either absent or less than intraspecific variation in 12 cases (Figs 5 and 6, Table 2). The four cases (Table 2) which completely lacked interspecific divergence may reflect taxonomic over-splitting or introgression, as discussed in Mutanen et al. (2016) [10].

**Table 2:** Nearest-Neighbor distances (% K2P) for 102 species of Lepidoptera as well as the mean and maximum intraspecific divergences for the new records obtained in the present study and DS-MARKALL dataset.

Species	N	Nearest Neighbor	Distance to NN	Max intra	Mean intra
*Acleris aspersana*	14	*Acleris shepherdana*	4.20	0.65	0.23
*Acompsia cinerella*	23	*Acompsia subpunctella*	3.45	2.03	0.90
*Acronicta auricoma*	24	*Acronicta rumicis*	5.67	0.31	0.09
*Aethes kindermanniana*	8	*Aethes smeathmanniana*	3.18	1.86	1.08
*Agrotis fatidica*	12	*Agrotis cinerea*	2.92	0.51	0.18
*Anaplectoides prasina*	20	*Eurois occulta*	3.86	0.31	0.03
*Apamea furva*	15	*Apamea platinea*	2.98	0.93	0.41
*Apamea lateritia*	18	*Apamea schildei*	3.56	0.46	0.11
*Arctia caja*	24	*Arctia flavia*	4.75	2.35	0.65
*Arctia flavia*	9	*Borearctia menetriesi*	3.30	0.50	0.21
*Argyresthia pygmaeella*	14	*Argyresthia curvella*	4.25	1.24	0.28
*Arichanna melanaria*	12	*Bupalus piniaria*	5.25	0.31	0.12
*Athrips pruinosella*	8	*Athrips mouffetella*	7.80	2.51	1.43
*Autographa pulchrina*	108	*Autographa buraetica*	0.88	0.96	0.17
*Boloria dia*	29	*Boloria titania*	5.79	0.77	0.33
*Boloria napaea*	15	*Boloria aquilonaris*	0.82	2.02	1.01
*Boloria titania*	26	*Boloria chariclea*	0.15	2.23	0.75
*Brenthis ino*	28	*Brenthis daphne*	0.62	2.19	0.47
*Carsia sororiata*	14	*Aplocera simpliciata*	9.59	0.96	0.54
*Caryocolum leucomelanella*	16	*Caryocolum mazeli*	3.79	1.55	0.78
*Caryocolum pullatella*	12	*Caryocolum marmorea*	3.20	3.64	2.02
*Catoptria languidellus*	9	*Catoptria digitellus*	7.98	1.87	0.99
*Celypha rivulana*	10	*Celypha flavipalpana*	4.74	1.08	0.54
*Cerapteryx graminis*	22	*Tholera decimalis*	3.77	0.80	0.39
*Charissa ambiguata*	14	*Charissa predotae*	1.43	1.94	0.86
*Chionodes distinctella*	31	*Chionodes continuella*	4.91	3.37	1.35
*Chionodes holosericella*	20	*Chionodes fumatella*	5.73	0.61	0.26
*Coenonympha glycerion*	27	*Coenonympha rhodopensis*	7.94	0.93	0.32
*Coenonympha tullia*	21	Coenonympha rhodopensis*	0.31	1.03	0.33
*Colostygia aptata*	22	*Colostygia aqueata*	6.57	1.08	0.43
*Coscinia cribraria*	44	*Euplagia quadripunctaria*	8.6	7.32	3.33
*Crambus perlella*	17	Crambus monochromellus*	0.00	1.08	0.39
*Crocallis elinguaria*	30	Crocallis albarracina*	0.00	7.17	1.39
*Cupido minimus*	43	*Cupido osiris*	3.48	0.62	0.23
*Cyaniris semiargus*	27	*Agriades glandon*	4.27	1.24	0.31
*Diarsia brunnea*	31	*Diarsia dahlii*	3.63	0.68	0.20
*Diarsia mendica*	37	*Diarsia dahlii*	3.30	5.29	1.87
*Dicallomera fascelina*	13	*Gynaephora selenitica*	7.42	3.81	1.51
*Eana osseana*	12	*Eana argentana*	2.81	4.27	1.83
*Eana penziana*	30	*Eana nervana*	3.46	1.15	0.33
*Eilema lutarella*	17	*Setema cereola*	2.50	0.46	0.13
*Elachista bedellella*	13	*Elachista lugdunensis*	2.26	1.87	0.90
*Entephria caesiata*	30	*Entephria nobiliaria*	4.92	1.72	0.70
*Epermenia illigerella*	16	*Epermenia falciformis*	7.90	1.55	0.74
*Epinotia cruciana*	14	*Epinotia mercuriana*	3.96	2.98	0.92
*Epinotia trigonella*	12	Epinotia indecorana*	0.00	2.51	1.05
*Eudonia alpina*	5	*Eudonia mercurella*	4.55	0.16	0.06
*Eulamprotes wilkella*	19	*Eulamprotes libertinella*	7.92	6.29	1.88
*Eulithis populata*	27	*Eulithis prunata*	5.77	0.80	0.25
*Eulithis prunata*	27	*Eulithis populata*	5.77	5.78	2.48
*Eulithis testata*	8	*Eulithis prunata*	6.00	0.80	0.37
*Eumedonia eumedon*	25	*Plebejus orbitulus*	4.05	2.82	1.09
*Euphyia unangulata*	16	*Euphyia adumbraria*	4.48	0.46	0.16
*Eupithecia pusillata*	40	*Eupithecia oxycedrata*	5.31	1.55	0.20
*Eurois occulta*	20	*Spaelotis suecica*	3.34	0.32	0.06
*Euxoa recussa*	15	*Euxoa vitta*	2.01	0.46	0.06
*Gazoryctra ganna*	8	*Gazoryctra fuscoargenteus*	8.14	4.28	2.07
*Graphiphora augur*	15	*Eurois occulta*	3.77	0.46	0.10
*Gypsonoma nitidulana*	18	*Archips crataegana*	7.30	2.50	1.36
*Hadena compta*	19	*Hadena magnolii*	2.38	0.92	0.16
*Lasionycta imbecilla*	17	*Papestra biren*	5.55	1.08	0.54
*Lasionycta proxima*	20	*Polia bombycina*	5.89	1.41	0.73
*Levipalpus hepatariella*	14	*Agonopterix cluniana*	7.05	0.92	0.43
*Lycaena virgaureae*	28	*Lycaena tityrus*	2.91	0.62	0.19
*Macaria brunneata*	21	*Macaria wauaria*	7.26	1.24	0.47
*Matilella fusca*	12	*Selagia spadicella*	5.29	0.77	0.24
*Miltochrista miniata*	28	*Eucarta virgo*	8.75	0.00	0.00
*Mompha locupletella*	12	*Mompha miscella*	9.28	0.46	0.08
*Monopis spilotella*	6	*Monopis laevigella*	8.06	1.86	1.03
*Noctua interposita*	24	*Noctua atlantica*	4.09	0.46	0.06
*Ochsenheimeria urella*	14	*Ochsenheimeria vacculella*	9.63	3.77	2.03
*Oidaematophorus rogenhoferi*	15	*Oidaematophorus vafradactylus*	10.68	1.08	0.51
*Papestra biren*	15	*Lacanobia oleracea*	3.47	0.31	0.09
*Parnassius phoebus*	17	*Parnassius apollo*	1.97	1.55	0.49
*Pediasia aridella*	9	*Pediasia truncatellus*	5.25	0.93	0.47
*Perizoma hydrata*	19	*Perizoma affinitata*	0.15	0.00	0.00
*Phiaris obsoletana*	7	*Phiaris metallicana*	1.08	0.31	0.09
*Plebejus orbitulus*	10	*Agriades glandon*	3.03	1.24	0.43
*Polia bombycina*	18	*Polia hepatica*	4.26	0.32	0.07
*Polypogon tentacularia*	17	*Zanclognatha zelleralis*	3.95	0.62	0.23
*Pontia callidice*	9	*Pieris bryoniae*	8.35	3.64	1.83
*Protolampra sobrina*	10	*Spaelotis suecica*	5.34	0.16	0.03
*Pyrausta aerealis*	15	*Anania crocealis*	7.82	4.60	0.96
*Scopula incanata*	22	*Scopula marginepunctata*	3.46	3.31	1.16
*Scopula virgulata*	13	*Calamodes subscudularia*	7.22	0.62	0.26
*Scotopteryx chenopodiata*	28	*Scotopteryx bipunctaria*	12.98	0.62	0.15
*Scrobipalpula diffluella*	14	*Scrobipalpula tussilaginis*	1.09	4.29	2.07
*Selagia spadicella*	10	*Ortholepis betulae*	3.45	1.55	0.83
*Setina irrorella*	23	*Setina aurita*	0.00	3.84	2.01
*Sparganothis pilleriana*	9	*Doloploca punctulana*	6.88	1.08	0.40
*Syngrapha ain*	14	*Syngrapha microgamma*	2.82	0.46	0.10
*Syngrapha hochenwarthi*	13	*Syngrapha interrogationis*	2.85	0.62	0.32
*Syngrapha interrogationis*	25	*Syngrapha hochenwarthi*	2.85	0.46	0.07
*Trichiura crataegi*	28	*Trichiura castiliana*	4.50	1.87	0.82
*Udea uliginosalis*	23	*Udea alpinalis*	1.08	3.64	1.77
*Xanthorhoe decoloraria*	16	*Xanthorhoe montanata*	5.40	1.08	0.59
*Xanthorhoe montanata*	31	*Xanthorhoe decoloraria*	5.40	2.67	0.43
*Xestia speciosa*	39	*Xestia viridescens*	3.01	2.96	1.44
*Yponomeuta evonymella*	14	*Yponomeuta cagnagella*	1.15	0.77	0.22
*Ypsolopha dentella*	10	*Ypsolopha falcella*	6.48	0.77	0.34
*Ypsolopha nemorella*	12	*Ypsolopha falcella*	4.58	1.39	0.54
*Zeiraphera griseana*	23	*Zeiraphera rufimitrana*	4.09	0.48	0.05

Species with an asterisk (*) indicate cases where the nearest neighbor may represent an example of taxonomic over-splitting (cf. [10]). ‘Mean intra’ values correspond to ‘total-intra’ values in Table 1.

## Discussion

Our analysis of DNA barcode sequences from a phylogenetically diverse group of Lepidoptera from Asia and Europe revealed that intraspecific divergences increased with sampling intensity and distance. However, intraspecific divergences in most species remained low with mean K2P divergences averaging 0.68% and exceeding 2.5% in 23 species of the complete sample. However, divergence was >2.5% in just 9 of the 102 species in one or more of the three spatial levels of our analysis. By comparison, the species with a higher divergence than 2.5% showed a mean sequence divergence of 4.62% to European populations of 5,016 species of Lepidoptera. This result corroborates patterns from earlier studies on North American [6] and European Lepidoptera [4], confirming that the barcode region of COI is an efficient tool for species identification, given that the databases are of high quality, even when the reference sequences used for species identification derive from sites far distant from the locality under study. Irrespective of their origin, most sequences could be unambiguously allocated to a taxonomically defined species although several cases of high intraspecific divergence may reflect overlooked species (as discussed later). Conversely, 4 of the 7 species pairs (*Crambus perlella*/*monochromella*, *Crocallis elinguaria*/*albarracina*, *Epinotia trigonella*/*indecorana*, *Coenonympha tullia*/*rhodopensis*) that either lacked or possessed very limited (<0.5%) divergence from their NN may indicate taxonomic over-splitting rather than the failure of DNA barcoding to discriminate valid species (see [10]). For three other species pairs (*Setina irrorella*/*aurita*, *Boloria titania*/*chariclea* and *Perizoma hydrata*/*affinitata*), the low NN values suggest a recent divergence of valid, morphologically well-defined species or recent mitochondrial introgression. For example, an earlier study suggested that the low NN divergence between *P*. *hydrata* and *P*. *affinitata* resulted from mitochondrial introgression from *P*. *hydrata* to *P*. *affinitata* [18].

Our comparisons of European and South Siberian populations revealed regional sequence divergence in the respective region in about half the species, but most values were well below 2%. In addition, regional barcode variation was similar in species with disjunct distributions and in those with continuous ranges, indicating substantial gene flow in both cases. In part, this may reflect the fact that current distributions of Euro-Siberian Lepidoptera largely result from range expansions in the brief interval since the last glacial maximum, i.e. within less than 15,000 years [19], [20]. However, when intraspecific sequence divergences were examined using an isolation-by-distance approach, they were slightly stronger in species with disjunct ranges.

Despite our limited sampling, some species (e.g. *Elachista bedellella*, *Boloria napaea*, *B*. *titania* and *Plebejus orbitulus*) showed clear divergence between South Siberian and European populations (see Table 1). In addition, populations of some species from northern Europe clustered with those from Asia rather than from central Europe (e.g. *Xestia speciosa*). This pattern likely indicates that formerly glaciated areas in northern Europe were sometimes recolonized by lineages from Asia. All these intraspecific patterns need to be examined in more detail by increased sampling effort in intermediate areas, and should be cross-checked using morphology and nuclear markers to clarify phylogeographic histories. Yet, for the purpose of species identification, we did not encounter any significant barriers, even in these taxa.

### High intraspecific divergences–potential cryptic diversity

High intraspecific barcode divergences (> 2–3%) may be indicative for the existence of overlooked species of Lepidoptera, but may also be due to mitochondrial introgression from a sister species [21]. Therefore, all such cases should be analyzed in more detail by examining divergence patterns at nuclear loci and morphological characters. We detected high intraspecific divergences (> 2.5% max divergence) between European and Asian populations for 9 of the 102 species (Table 3). Six of these species have a disjunct distribution, suggesting the possible existence of cryptic species in South Siberia versus Europe. In three other species (e.g. *Coscinia cribraria*), barcode variation was high even within Europe without an obvious geographical pattern. The remainder of this section discusses these nine species in more detail. All of them group into two or more different BINs [3] (S1 Table), operational taxonomic units which in Lepidoptera are frequently but not always congruent with species boundaries (e.g. [7], [22]). In fact deep barcode splits may be caused by pseudogenes, *Wolbachia* infection, hybridization etc. [23] and these cases need to be analysed using an integrative approach (e.g., [24]).

**Table 3:** Nine Euro-Siberian species of Lepidoptera with a max intra-specific K2P distance for COI >2.5% between Asia and Europe.

Species	total-Eurasia	intra-Asia	intra-Europe	inter-Europe-Asia
*Caryocolum pullatella*	**3.1**	2.3	2.0	**3.1**
*Coscinia cribraria*	**3.5**	1.0	**3.5**	**3.2**
*Dicallomera fascelina*	1.5	0.5	0.2	**3.5**
*Eana osseana*	**3.0**	0.1	0.4	**3.9**
*Eulithis prunata*	2.5	0.0	2.0	**4.3**
*Gazoryctra ganna*	2.2	1.4	1.1	**3.6**
*Ochsenheimeria urella*	2.1	0.2	0.21	**2.5**
*Pontia callidice*	1.8	0.1	0.2	**3.4**
*Scrobipalpula diffluella*	2.1	0.2	0.5	**3.6**

#### 1. *Caryocolum pullatella* (Tengström, 1848) (Gelechiidae)

*C*. *pullatella* is a Holarctic species that is widespread in northern Europe, but restricted to isolated localities in the Alps and Balkans [25]. As its Palearctic populations include two DNA barcode clusters with allopatric distributions (central/south-east Europe versus north Europe-South Siberia), this may indicate cryptic diversity. The situation potentially gains further complexity when North American specimens are considered as they include additional BINs and requires further assessment.

#### 2. *Coscinia cribraria* (Linnaeus, 1758) (Erebidae)

This morphologically variable species is widely distributed across the Palearctic. Numerous forms and subspecies have been described, including ssp. *sibirica* (Staudinger, 1892) from the Altai Mountains which was recently synonymized by Dubatolov (2010) [26]. However, Witt & Ronkay (2011) [27] suspected that sequence data would indicate the existence of a species complex. Current DNA barcode sequences are assigned to five clades; specimens from Altai belong to the same BIN as those from northern and central Europe. As the clusters within Europe do not show a clear phylogeographic pattern, sequence variation may indicate introgression or the impacts of *Wolbachia* infection [23].

#### 3. *Dicallomera fascelina* (Linnaeus, 1758) (Erebidae)

*D*. *fascelina* is almost continuously distributed in temperate Eurasia, extending from northern Spain east to Korea, although absent from the Mediterranean region and the British Isles. Several subspecies have been recognized. Populations from the Altai region have been attributed to the nominotypical subspecies, but the clear differences in their external morphology and genitalia [28], coupled with their barcode divergence, suggest they represent a cryptic species.

#### 4. *Eana osseana* (Scopoli, 1763) (Tortricidae)

*E*. *osseana* is a widespread Holarctic species, restricted to mountainous areas at the southern limits of its distribution. DNA barcodes indicate two divergent BINs, one from Europe, and a second from the Altai Mountains. As three additional BINs are known from North America, the species requires integrative revisionary work.

#### 5. *Eulithis prunata* (Linnaeus, 1758) (Geometridae)

This species is almost continuously distributed in temperate Eurasia, but is restricted to mountainous areas in the southern parts of its range. Hausmann & Viidalepp (2012) [29] found high COI sequence divergence in *E*. *prunata*, with distances reaching 5.9% and at least six divergent haplotypes in Europe and Turkey. South Siberian populations have been assigned to the ssp. *leucoptera* (Djakonov, 1929), but it may represent a distinct species given its deep barcode divergence from other populations.

#### 6. *Gazoryctra ganna* (Hübner, 1804) (Hepialidae)

*G*. *ganna* is an arctic-alpine species with a disjunct distribution. It occurs in the Alps and High Tatra Mountains, northern Finland, and European Russia, as well as at isolated localities to the Far East [30]. Moderate sequence divergence exists between northern and central European populations [8] while those from the Altai Mountains show high sequence divergence from both European clusters. Because of their differing flight times (late afternoon in Asia versus early morning in Europe) and slightly different phenotypes, the Asian specimens likely represent an overlooked species.

#### 7. *Ochsenheimeria urella* (Fischer von Röslerstamm, 1842) (Ypsolophidae)

*O*. *urella* is widely although locally distributed in central and northern Europe, including European Russia. A previously doubtful record from the Far East [30], together with our record from Altai [11], indicates a much wider distribution in Asia. Members of this species are placed in two BINs, one shared by the Alps and Finland, and the other by Finland and the Altai Mountains.

#### 8. *Pontia callidice* (Hübner, 1800) (Pieridae)

*P*. *callidice* shows a disjunct distribution in the high mountains of Eurasia from the Pyrenees to the Himalayas, and in the subarctic Tundra from the Ural Mountains to the Far East. Linked to their geographic isolation, populations show considerable variation in wing patterns and have been assigned to several subspecies. The nominotypical subspecies occurs in the high mountains of Europe (Pyrenees and Alps). Della Bruna et al. (2004) [31] assigned populations from the Altai to spp. *hinducucica* Verity, 1811 (type locality Hindu Kush), whereas Tshikolovets et al. (2009) [32] listed spp. *kalora* (Moore, 1865) from Altai (type locality NW Himalaya). Korb & Bolshakov (2016) [33] listed ssp. *halasia* Huang et Murayama, 1992 from SW Altai (described from Halasi, [Chinese] Altai). Despite this nomenclatural uncertainty, the DNA barcode results indicate that specimens from the Alps belong to a very distinct barcode cluster from those in Russia (Altai), Kyrgyzstan and Tajikistan.

#### 9. *Scrobipalpula diffluella* (Frey, 1870) (Gelechiidae)

In the Palearctic, the genus *Scrobipalpula* includes a complex of closely related species with disputed taxonomy [25]. *S*. *diffluella* shows a typical boreo-montane distribution with most records from northern and central Europe, extending to the southern Urals. Specimens of the newly detected population from the Altai show close morphological similarity with European material, but clear barcode divergence, suggesting cryptic diversity.

## Conclusions

This study on a phylogenetically diverse sample of Lepidoptera across a wide geographic range within the Palearctic region corroborates the utility of DNA barcode data for enabling both species identification and species discovery. For most species, unequivocal identifications could be established for samples from a widely distant region (the Russian Altai mountains), even though available reference data largely derived from regions in north and central Europe. On the other hand, in a few ‘species’ taxonomically known since Linnean times, patterns of sequence divergence suggest the possibility of unrecognized cryptic species diversity and demand further assessment using an integrative taxonomic approach. Hence, this study exemplifies the usefulness of well curated DNA barcode libraries whose power and versatility will expand as more sequence data are collated under strict quality standards.

## Supporting information

S1 Table Accession numbers and BINs. List of species names, sample-IDs, process-IDs (from BOLD database), GenBank Accession numbers, BINs, and Institution/collection storing vouchers.
(PDF)
