# Supplementary material for: Large geographic distance versus small DNA barcode divergence: Insights from a comparison of European to South Siberian Lepidoptera
Source: PLoS One. 2018 Nov 2;13(11):e0206668. doi: 10.1371/journal.pone.0206668 (PMC6214556; doi:10.1371/journal.pone.0206668)
Supplement: S1 Table — List of species names, sample-IDs, process-IDs (from BOLD database), GenBank Accession numbers, BINs, and Institution/collection storing vouchers. (PDF) [file pone.0206668.s001.pdf]

S1 Table. Accession numbers and BINs. List of species names, sample-IDs, process-IDs (from BOLD database), GenBank Accession numbers, BINs, and Institution/collection storing vouchers.

Species	Process ID	Sample ID	GenBank	BIN	Institution
Acleris aspersana	LASTS219-14	TLMF Lep 14671	KP253567	BOLD:AAB3526	Research Collection of Toni Mayr
Acleris aspersana	LEALT251-16	TLMF Lep 20474	MG522446	BOLD:AAB3526	Tiroler Landesmuseum
Acleris aspersana	LEATJ873-15	TLMF Lep 18733	MG522237	BOLD:AAB3526	Tiroler Landesmuseum
Acleris aspersana	LEFIC736-10	MM04833	HM872557	BOLD:AAB3526	University of Oulu
Acleris aspersana	LEALT252-16	TLMF Lep 20475	MG522594	BOLD:AAB3526	Tiroler Landesmuseum
Acleris aspersana	LEFID855-10	MM06981	HM873612	BOLD:AAB3526	University of Oulu
Acleris aspersana	LEATI315-15	TLMF Lep 17795	MG522667	BOLD:AAB3526	Tiroler Landesmuseum
Acleris aspersana	LEFIB473-10	MM02024	HM871372	BOLD:AAB3526	University of Oulu
Acleris aspersana	ABOLB349-15	TLMF Lep 17354	MG522752	BOLD:AAB3526	Tiroler Landesmuseum
Acleris aspersana	LEATJ1025-15	TLMF Lep 18885	MG522498	BOLD:AAB3526	Tiroler Landesmuseum
Acleris aspersana	LEFIG535-10	MM14665	HM876208	BOLD:AAB3526	University of Oulu
Acleris aspersana	LEALT253-16	TLMF Lep 20476	MG522071	BOLD:AAB3526	Tiroler Landesmuseum
Acleris aspersana	PHLAI056-12	KLM Lep 00531	MG522181	BOLD:AAB3526	Landesmuseum Kaernten
Acleris aspersana	LEFIF898-10	MM13312	HM875580	BOLD:AAB3526	University of Oulu
Acompsia cinerella	PHLAB107-10	TLMF Lep 00907	HM381479	BOLD:AAD0078	Tiroler Landesmuseum
Acompsia cinerella	PHLAB649-10	TLMF Lep 01449	HQ968667	BOLD:AAD0078	Tiroler Landesmuseum
Acompsia cinerella	LEALT067-16	TLMF Lep 20290	MG522586	BOLD:AAD0078	Tiroler Landesmuseum
Acompsia cinerella	FBLMZ372-12	BC ZSM Lep 64453	KX046922	BOLD:AAD0078	Research Collection of Theo Gruenewald
Acompsia cinerella	PHLAC894-10	TLMF Lep 02929	JF860370	BOLD:AAD0078	Tiroler Landesmuseum
Acompsia cinerella	LEATE765-13	TLMF Lep 12177	MG522038	BOLD:AAD0078	Tiroler Landesmuseum
Acompsia cinerella	PHLAB685-10	TLMF Lep 01485	HQ968699	BOLD:AAD0078	Tiroler Landesmuseum
Acompsia cinerella	PHLAG121-12	TLMF Lep 06431	MG522370	BOLD:AAD0078	Tiroler Landesmuseum
Acompsia cinerella	PHLAB686-10	TLMF Lep 01486	HQ968700	BOLD:AAD0078	Tiroler Landesmuseum
Acompsia cinerella	LEFIF951-10	MM13521	HM875632	BOLD:AAD0078	University of Oulu
Acompsia cinerella	GMGMM1300-14	BIOUG17377-E01	MG522818	BOLD:AAD0078	Zoologisches Forschungsmuseum Alexander Koenig
Acompsia cinerella	PHLAF063-11	TLMF Lep 05233	MG522738	BOLD:AAD0078	Tiroler Landesmuseum

Acompsia cinerella	LEFIB725-10	MM02545	HM871603	BOLD:AAD0078	University of Oulu
Acompsia cinerella	LEATF034-14	TLMF Lep 13346	MG522267	BOLD:AAD0078	Tiroler Landesmuseum
Acompsia cinerella	PHLAH533-12	TLMF Lep 08352	MG522028	BOLD:AAD0078	Tiroler Landesmuseum
Acompsia cinerella	LEFIC841-10	MM05048	HM872659	BOLD:AAD0078	University of Oulu
Acompsia cinerella	PHLAF064-11	TLMF Lep 05234	MG522652	BOLD:AAD0078	Tiroler Landesmuseum
Acompsia cinerella	PHLAB103-10	TLMF Lep 00903	HM381475	BOLD:AAD0078	Tiroler Landesmuseum
Acompsia cinerella	LEALT054-16	TLMF Lep 20277	MG522315	BOLD:AAD0078	Tiroler Landesmuseum
Acompsia cinerella	PHLAV284-12	TLMF Lep 08103	KM572568	BOLD:AAD0078	inatura, Dornbirn
Acompsia cinerella	LEALT053-16	TLMF Lep 20276	MG522159	BOLD:AAD0078	Tiroler Landesmuseum
Acompsia cinerella	ABOLB193-15	TLMF Lep 17198	MG522686	BOLD:AAD0078	Tiroler Landesmuseum
Acompsia cinerella	PHLAB092-10	TLMF Lep 00892	HQ968266	BOLD:AAD0078	Tiroler Landesmuseum
Acronicta auricoma	ABOLC162-16	TLMF Lep 20195	MG522464	BOLD:ACF2281	Tiroler Landesmuseum
Acronicta auricoma	PHLAE313-11	TLMF Lep 04628	JN261551	BOLD:ACF2281	Tiroler Landesmuseum
Acronicta auricoma	GBLAA204-14	BC ZSM Lep 80200	MG521949	BOLD:ACF2281	SNSB, Zoologische Staatssammlung Muenchen
Acronicta auricoma	NLLEA097-12	RMNH.INS.538712	KX048207	BOLD:ACF2281	Naturalis Biodiversity Centre
Acronicta auricoma	GBLAA1400-15	BC ZSM Lep 87096	MG522133	BOLD:ACF2281	SNSB, Zoologische Staatssammlung Muenchen
Acronicta auricoma	GWOTL271-13	BC ZSM Lep 67297	MG522303	BOLD:ACF2281	SNSB, Zoologische Staatssammlung Muenchen
Acronicta auricoma	GBLAF527-14	BC ZSM Lep 82233	MG522098	BOLD:ACF2281	SNSB, Zoologische Staatssammlung Muenchen
Acronicta auricoma	GBLAC520-13	BC ZSM Lep 78141	MG522487	BOLD:ACF2281	SNSB, Zoologische Staatssammlung Muenchen
Acronicta auricoma	LEATB455-13	TLMF Lep 10632	MG522826	BOLD:ACF2281	Tiroler Landesmuseum
Acronicta auricoma	ABOLA009-14	KLM Lep 01529	MG522276	BOLD:ACF2281	Landesmuseum Kaernten
Acronicta auricoma	ABOLA896-15	TLMF Lep 16856	MG522373	BOLD:ACF2281	Tiroler Landesmuseum
Acronicta auricoma	LEFIE050-10	MM08162	HM873798	BOLD:ACF2281	University of Oulu
Acronicta auricoma	GBLGC350-12	BC ZSM Lep R 21680	MG522015	BOLD:ACF2281	SNSB, Zoologische Staatssammlung Muenchen
Acronicta auricoma	GBLGC349-12	BC ZSM Lep R 21679	MG522673	BOLD:ACF2281	SNSB, Zoologische Staatssammlung Muenchen
Acronicta auricoma	LEFIC581-10	MM04363	HM872402	BOLD:ACF2281	University of Oulu
Acronicta auricoma	GWORK255-09	BC ZSM Lep 21680	JF415742	BOLD:ACF2281	SNSB, Zoologische Staatssammlung Muenchen
Acronicta auricoma	LEATD363-13	TLMF Lep 13010	MG522476	BOLD:ACF2281	Tiroler Landesmuseum

Acronicta auricoma	LEFID393-10	MM06306	HM873190	BOLD:ACF2281	University of Oulu
Acronicta auricoma	LEATB640-13	TLMF Lep 10817	MG522203	BOLD:ACF2281	Naturmuseum Suedtirol
Acronicta auricoma	GBLAA1399-15	BC ZSM Lep 87095	MG522316	BOLD:ACF2281	SNSB, Zoologische Staatssammlung Muenchen
Acronicta auricoma	ABOLA736-14	TLMF Lep 16423	MG522801	BOLD:ACF2281	Tiroler Landesmuseum
Acronicta auricoma	NLLEA105-12	RMNH.INS.538720	KX049751	BOLD:ACF2281	Naturalis Biodiversity Centre
Acronicta auricoma	LEALT508-16	TLMF Lep 20731	MG522423	BOLD:ACF2281	Tiroler Landesmuseum
Acronicta auricoma	LEALT507-16	TLMF Lep 20730	MG522563	BOLD:ACF2281	Tiroler Landesmuseum
Aethes kindermanniana	LEALT207-16	TLMF Lep 20430	MG521968	BOLD:AAF5176	Tiroler Landesmuseum
Aethes kindermanniana	LEALT208-16	TLMF Lep 20431	MG522246	BOLD:AAF5176	Tiroler Landesmuseum
Aethes kindermanniana	BTLBP139-11	CCDB-11132-D08	MG522756	BOLD:AAF5176	Sofia University, St. Kliment Ohridski
Aethes kindermanniana	LEFIC450-10	MM04010	HM872284	BOLD:AAF5176	University of Oulu
Aethes kindermanniana	BTLBP138-11	CCDB-11132-D07	MG522499	BOLD:AAF5176	Sofia University, St. Kliment Ohridski
Aethes kindermanniana	LEFIC253-10	MM03668	HM872097	BOLD:AAF5176	University of Oulu
Aethes kindermanniana	BTLBP137-11	CCDB-11132-D06	MG522072	BOLD:AAF5176	Sofia University, St. Kliment Ohridski
Aethes kindermanniana	LEFIF880-10	MM13281	HM875562	BOLD:AAF5176	University of Oulu
Agriades orbitulus	GBLAC373-13	BC ZSM Lep 75239	MG522682	BOLD:AAE5039	SNSB, Zoologische Staatssammlung Muenchen
Agriades orbitulus	LEATG098-14	TLMF Lep 13885	MG521921	BOLD:AAE5039	Tiroler Landesmuseum
Agriades orbitulus	PHLSA728-11	TLMF Lep 06183	KP253495	BOLD:AAE5039	Tiroler Landesmuseum
Agriades orbitulus	PHLAA136-09	TLMF Lep 00176	GU689154	BOLD:AAE5039	Tiroler Landesmuseum
Agriades orbitulus	LEATG093-14	TLMF Lep 13880	MG522061	BOLD:AAE5039	Tiroler Landesmuseum
Agriades orbitulus	LEATG418-14	TLMF Lep 14205	MG522439	BOLD:AAE5039	Naturmuseum Suedtirol
Agriades orbitulus	PHLAC715-10	TLMF Lep 02750	JF860268	BOLD:AAE5039	Tiroler Landesmuseum
Agrotis fatidica	LEALT300-16	TLMF Lep 20523	MG522362	BOLD:AAA1629	Tiroler Landesmuseum
Agrotis fatidica	ABOLB825-15	KLM Lep 03485	MG522016	BOLD:AAA1629	Landesmuseum Kaernten
Agrotis fatidica	PHLAC667-10	TLMF Lep 02702	JF860232	BOLD:AAA1629	Tiroler Landesmuseum
Agrotis fatidica	LEALT301-16	TLMF Lep 20524	MG522834	BOLD:AAA1629	Tiroler Landesmuseum
Agrotis fatidica	PHLAA144-09	TLMF Lep 00184	GU689194	BOLD:AAA1629	Tiroler Landesmuseum
Agrotis fatidica	PHLAA145-09	TLMF Lep 00185	GU689152	BOLD:AAA1629	Tiroler Landesmuseum
Agrotis fatidica	PHLAA126-09	TLMF Lep 00166	GU689162	BOLD:AAA1629	Tiroler Landesmuseum
Agrotis fatidica	PHLAC937-10	TLMF Lep 02972	JF860405	BOLD:AAA1629	Tiroler Landesmuseum

Agrotis fatidica	PHLAA125-09	TLMF Lep 00165	GU689161	BOLD:AAA1629	Tiroler Landesmuseum
Agrotis fatidica	LEALT302-16	TLMF Lep 20525	MG522636	BOLD:AAA1629	Tiroler Landesmuseum
Agrotis fatidica	LEATH567-14	TLMF Lep 15779	MG522272	BOLD:AAA1629	Naturmuseum Suedtirol
Agrotis fatidica	LEATH568-14	TLMF Lep 15780	MG522749	BOLD:AAA1629	Naturmuseum Suedtirol
Anaplectoides prasina	GWORO839-09	BC ZSM Lep 30531	GU688485	BOLD:AAA2948	SNSB, Zoologische Staatssammlung Muenchen
Anaplectoides prasina	GWORK390-09	BC ZSM Lep 21720	JF415468	BOLD:AAA2948	SNSB, Zoologische Staatssammlung Muenchen
Anaplectoides prasina	LEFIA549-10	MM01638	KM572060	BOLD:AAA2948	University of Oulu
Anaplectoides prasina	GBLAA207-14	BC ZSM Lep 80203	MG522205	BOLD:AAA2948	SNSB, Zoologische Staatssammlung Muenchen
Anaplectoides prasina	ABOLC028-16	TLMF Lep 20061	MG522428	BOLD:AAA2948	Tiroler Landesmuseum
Anaplectoides prasina	PHLAC367-10	TLMF Lep 02402	KX042475	BOLD:AAA2948	Tiroler Landesmuseum
Anaplectoides prasina	LEALT499-16	TLMF Lep 20722	MG522067	BOLD:AAA2948	Tiroler Landesmuseum
Anaplectoides prasina	PHLAV317-12	TLMF Lep 08136	KM573497	BOLD:AAA2948	inatura, Dornbirn
Anaplectoides prasina	LEATB574-13	TLMF Lep 10751	MG521999	BOLD:AAA2948	Naturmuseum Suedtirol
Anaplectoides prasina	LEATD236-13	TLMF Lep 12883	MG522366	BOLD:AAA2948	Tiroler Landesmuseum
Anaplectoides prasina	LEFIC855-10	MM05089	HM872673	BOLD:AAA2948	University of Oulu
Anaplectoides prasina	LEFIA550-10	MM01639	KM571996	BOLD:AAA2948	University of Oulu
Anaplectoides prasina	GWORA2541-09	BC ZSM Lep 31798	HQ957223	BOLD:AAA2948	Research Collection of Ralph Sturm
Anaplectoides prasina	ABOLB525-15	KLM Lep 02710	MG522115	BOLD:AAA2948	Landesmuseum Kaernten
Anaplectoides prasina	GBLAD169-14	BC ZSM Lep 78740	MG522027	BOLD:AAA2948	SNSB, Zoologische Staatssammlung Muenchen
Anaplectoides prasina	LEATC192-13	TLMF Lep 11319	MG521916	BOLD:AAA2948	Tiroler Landesmuseum
Anaplectoides prasina	GWORK453-09	BC ZSM Lep 21783	JF415469	BOLD:AAA2948	SNSB, Zoologische Staatssammlung Muenchen
Anaplectoides prasina	LEFIF641-10	MM12547	HM875325	BOLD:AAA2948	University of Oulu
Anaplectoides prasina	LEALT477-16	TLMF Lep 20700	MG522493	BOLD:AAA2948	Tiroler Landesmuseum
Anaplectoides prasina	GWORL340-09	BC ZSM Lep 22052	GU686928	BOLD:AAA2948	SNSB, Zoologische Staatssammlung Muenchen
Apamea furva	LEFIK458-10	MM18033	JF854075	BOLD:AAC7157	University of Oulu
Apamea furva	LEATG135-14	TLMF Lep 13922	MG521938	BOLD:AAC7157	Tiroler Landesmuseum
Apamea furva	LEATC169-13	TLMF Lep 11296	MG522210	BOLD:AAC7157	Naturmuseum Suedtirol
Apamea furva	ABOLB454-15	KLM Lep 02639	MG522193	BOLD:AAC7157	Landesmuseum Kaernten
Apamea furva	LEATG134-14	TLMF Lep 13921	MG522070	BOLD:AAC7157	Tiroler Landesmuseum
Apamea furva	LEALT541-16	TLMF Lep 20764	MG522598	BOLD:AAC7157	Tiroler Landesmuseum

Apamea furva	PHLAF360-11	TLMF Lep 05530	MG522477	BOLD:AAC7157	Tiroler Landesmuseum
Apamea furva	PHLAI275-13	TLMF Lep 08770	KM572209	BOLD:AAC7157	Tiroler Landesmuseum
Apamea furva	PHLAH819-12	TLMF Lep 08623	MG522339	BOLD:AAC7157	Tiroler Landesmuseum
Apamea furva	LEFIF191-10	MM10783	HM874885	BOLD:AAC7157	University of Oulu
Apamea furva	LEALT356-16	TLMF Lep 20579	MG522173	BOLD:AAC7157	Tiroler Landesmuseum
Apamea furva	LEATB579-13	TLMF Lep 10756	MG522534	BOLD:AAC7157	Naturmuseum Suedtirol
Apamea furva	PHLAF276-11	TLMF Lep 05446	MG521983	BOLD:AAC7157	Tiroler Landesmuseum
Apamea furva	LEFIC777-10	MM04908	HM872596	BOLD:AAC7157	University of Oulu
Apamea furva	LEALT348-16	TLMF Lep 20571	MG522792	BOLD:AAC7157	Tiroler Landesmuseum
Apamea lateritia	LEFIA621-10	MM01735	HM870870	BOLD:ACF5481	University of Oulu
Apamea lateritia	LEATB447-13	TLMF Lep 10624	MG522110	BOLD:ACF5481	Tiroler Landesmuseum
Apamea lateritia	LEALT481-16	TLMF Lep 20704	MG522364	BOLD:ACF5481	Tiroler Landesmuseum
Apamea lateritia	GWORO706-09	BC ZSM Lep 27642	JF415482	BOLD:ACF5481	SNSB, Zoologische Staatssammlung Muenchen
Apamea lateritia	LEATG132-14	TLMF Lep 13919	MG521975	BOLD:ACF5481	Tiroler Landesmuseum
Apamea lateritia	LEFIJ2398-14	MM23781	MG522092	BOLD:ACF5481	University of Oulu
Apamea lateritia	LEATG133-14	TLMF Lep 13920	MG522325	BOLD:ACF5481	Tiroler Landesmuseum
Apamea lateritia	PHLAF384-11	TLMF Lep 05554	MG522190	BOLD:ACF5481	Tiroler Landesmuseum
Apamea lateritia	LEATB547-13	TLMF Lep 10724	MG522764	BOLD:ACF5481	Naturmuseum Suedtirol
Apamea lateritia	PHLAH670-12	TLMF Lep 08489	KM572198	BOLD:ACF5481	inatura, Dornbirn
Apamea lateritia	LEFIA620-10	MM01734	HM870869	BOLD:ACF5481	University of Oulu
Apamea lateritia	LEALT338-16	TLMF Lep 20561	MG522794	BOLD:ACF5481	Tiroler Landesmuseum
Apamea lateritia	PHLAJ053-13	KLM Lep 00813	MG522324	BOLD:ACF5481	Landesmuseum Kaernten
Apamea lateritia	LEFIE775-10	MM09935	HM874494	BOLD:ACF5481	University of Oulu
Apamea lateritia	LEATD475-13	TLMF Lep 13122	MG522269	BOLD:ACF5481	Naturmuseum Suedtirol
Apamea lateritia	LEALT413-16	TLMF Lep 20636	MG522824	BOLD:ACF5481	Tiroler Landesmuseum
Apamea lateritia	ABOLB455-15	KLM Lep 02640	MG522271	BOLD:ACF5481	Landesmuseum Kaernten
Apamea lateritia	LEATB577-13	TLMF Lep 10754	MG522784	BOLD:ACF5481	Naturmuseum Suedtirol
Arctia caja	PHLSA661-11	TLMF Lep 06116	KM572296	BOLD:AAA8530	Tiroler Landesmuseum
Arctia caja	GBLAF517-14	BC ZSM Lep 82223	MG522363	BOLD:AAA8530	SNSB, Zoologische Staatssammlung Muenchen
Arctia caja	LEATG183-14	TLMF Lep 13970	MG521926	BOLD:AAA8530	Tiroler Landesmuseum

Arctia caja	GWOTL161-13	BC ZSM Lep 67092	MG522301	BOLD:AAA8530	SNSB, Zoologische Staatssammlung Muenchen
Arctia caja	LEEUA664-11	MM20723	KM573193	BOLD:AAA8530	University of Oulu
Arctia caja	LEATH576-14	TLMF Lep 15788	MG522355	BOLD:AAA8530	Naturmuseum Suedtirol
Arctia caja	GBLAD206-14	BC ZSM Lep 78777	MG522156	BOLD:AAA8530	SNSB, Zoologische Staatssammlung Muenchen
Arctia caja	FGMLB659-13	BC ZSM Lep 72295	MG522093	BOLD:AAA8530	SNSB, Zoologische Staatssammlung Muenchen
Arctia caja	FBLMU561-09	BC ZSM Lep 27211	GU707163	BOLD:AAA8530	SNSB, Zoologische Staatssammlung Muenchen
Arctia caja	GWORA2556-09	BC ZSM Lep 31813	HM393250	BOLD:AAA8530	Research Collection of Ralph Sturm
Arctia caja	LEALT310-16	TLMF Lep 20533	MG522165	BOLD:AAA8530	Tiroler Landesmuseum
Arctia caja	GBLAA359-14	BC ZSM Lep 80355	MG522440	BOLD:AAA8530	SNSB, Zoologische Staatssammlung Muenchen
Arctia caja	LEALT397-16	TLMF Lep 20620	MG522488	BOLD:AAA8530	Tiroler Landesmuseum
Arctia caja	NLLEA809-12	RMNH.INS.541004	KX047607	BOLD:AAA8530	Naturalis Biodiversity Centre
Arctia caja	NLLEA807-12	RMNH.INS.541002	KX048504	BOLD:AAA8530	Naturalis Biodiversity Centre
Arctia caja	NLLEA802-12	RMNH.INS.540997	KX048322	BOLD:AAA8530	Naturalis Biodiversity Centre
Arctia caja	LEFID959-10	MM07925	HM873709	BOLD:AAA8530	University of Oulu
Arctia caja	LEATG181-14	TLMF Lep 13968	MG522790	BOLD:AAA8530	Tiroler Landesmuseum
Arctia caja	LEFIC785-10	MM04921	HM872604	BOLD:AAA8530	University of Oulu
Arctia caja	FBLMZ510-12	BC ZSM Lep 61266	KX044660	BOLD:AAA8530	SNSB, Zoologische Staatssammlung Muenchen
Arctia caja	LEFIA864-10	MM09741	HM387002	BOLD:AAA8530	University of Oulu
Arctia caja	LEALT309-16	TLMF Lep 20532	MG522701	BOLD:AAA8530	Tiroler Landesmuseum
Arctia caja	LEATB711-13	TLMF Lep 10888	MG522420	BOLD:AAA8530	Naturmuseum Suedtirol
Arctia caja	ABOLA145-14	KLM Lep 01665	MG522623	BOLD:AAA8530	Landesmuseum Kaernten
Arctia flavia	LEALT308-16	TLMF Lep 20531	MG522805	BOLD:AAV9830	Tiroler Landesmuseum
Arctia flavia	PHLAE214-11	TLMF Lep 04434	JN262975	BOLD:AAV9830	Tiroler Landesmuseum
Arctia flavia	LEALT306-16	TLMF Lep 20529	MG522134	BOLD:AAV9830	Tiroler Landesmuseum
Arctia flavia	ABOLB543-15	KLM Lep 02728	MG522405	BOLD:AAV9830	Landesmuseum Kaernten
Arctia flavia	ABOLA872-15	TLMF Lep 16832	MG522590	BOLD:AAV9830	Tiroler Landesmuseum
Arctia flavia	ABOLA435-14	TLMF Lep 16122	MG522343	BOLD:AAV9830	Tiroler Landesmuseum
Arctia flavia	LEATC085-13	TLMF Lep 11212	MG522642	BOLD:AAV9830	Naturmuseum Suedtirol
Arctia flavia	LEALT307-16	TLMF Lep 20530	MG522179	BOLD:AAV9830	Tiroler Landesmuseum
Arctia flavia	PHLAI348-13	TLMF Lep 08843	MG522225	BOLD:AAV9830	Tiroler Landesmuseum

Argyresthia pygmaeella	LEALT028-16	TLMF Lep 20251	MG522250	BOLD:ADD9964	Tiroler Landesmuseum
Argyresthia pygmaeella	FBLMZ258-12	BC ZSM Lep 64339	KX046515	BOLD:ADD9964	Research Collection of Theo Gruenewald
Argyresthia pygmaeella	PHLAG283-12	TLMF Lep 06593	MG522459	BOLD:ADD9964	Research Collection of Peter Buchner
Argyresthia pygmaeella	FBLMZ082-12	BC ZSM Lep 61408	MG522183	BOLD:ADD9964	Research Collection of Richard Heindel
Argyresthia pygmaeella	LEALT030-16	TLMF Lep 20253	MG522793	BOLD:ADD9964	Tiroler Landesmuseum
Argyresthia pygmaeella	LEFIB743-10	MM02585	HM871621	BOLD:ADD9964	University of Oulu
Argyresthia pygmaeella	LEATI208-15	TLMF Lep 17593	MG522333	BOLD:ADD9964	Tiroler Landesmuseum
Argyresthia pygmaeella	LEATC293-13	TLMF Lep 12275	MG521967	BOLD:ADD9964	Naturmuseum Suedtirol
Argyresthia pygmaeella	LEFIB960-10	MM03135	HM871836	BOLD:ADD9964	University of Oulu
Argyresthia pygmaeella	LEFIB967-10	MM03153	HM871841	BOLD:ADD9964	University of Oulu
Argyresthia pygmaeella	LEATE334-13	TLMF Lep 11746	MG522426	BOLD:ADD9964	Tiroler Landesmuseum
Argyresthia pygmaeella	LEALT029-16	TLMF Lep 20252	MG522058	BOLD:ADD9964	Tiroler Landesmuseum
Argyresthia pygmaeella	PHLAF159-11	TLMF Lep 05329	MG522424	BOLD:ADD9964	Tiroler Landesmuseum
Argyresthia pygmaeella	FBLMZ630-12	BC ZSM Lep 61481	KX046095	BOLD:ADD9964	Research Collection of Peter Lichtmannecker
Arichanna melanaria	GBLGC110-12	BC ZSM Lep R 21060	MG522100	BOLD:AAC8717	SNSB, Zoologische Staatssammlung Muenchen
Arichanna melanaria	LEFIA306-10	MM01352	HM386649	BOLD:AAC8717	University of Oulu
Arichanna melanaria	LEFIE035-10	MM08115	HM873784	BOLD:AAC8717	University of Oulu
Arichanna melanaria	LEALT441-16	TLMF Lep 20664	MG521955	BOLD:AAC8717	Tiroler Landesmuseum
Arichanna melanaria	LEATB499-13	TLMF Lep 10676	MG522139	BOLD:AAC8717	Tiroler Landesmuseum
Arichanna melanaria	GWORE2222-09	BC ZSM Lep 24045	GU654869	BOLD:AAC8717	Research Collection of Alfred Haslberger
Arichanna melanaria	LEALT497-16	TLMF Lep 20720	MG522608	BOLD:AAC8717	Tiroler Landesmuseum
Arichanna melanaria	GWOR3776-09	BC ZSM Lep 21060	HQ601063	BOLD:AAC8717	SNSB, Zoologische Staatssammlung Muenchen
Arichanna melanaria	GWORD1063-08	BC ZSM Lep 01815	KX071570	BOLD:AAC8717	SNSB, Zoologische Staatssammlung Muenchen
Arichanna melanaria	PHLAC842-10	TLMF Lep 02877	JF860339	BOLD:AAC8717	Tiroler Landesmuseum
Arichanna melanaria	LEALT498-16	TLMF Lep 20721	MG522256	BOLD:AAC8717	Tiroler Landesmuseum
Arichanna melanaria	LEFIA305-10	MM01351	HM386648	BOLD:AAC8717	University of Oulu
Athrips pruinosella	LEALT050-16	TLMF Lep 20273	MG522600	BOLD:AAD2577	Tiroler Landesmuseum
Athrips pruinosella	LEALT056-16	TLMF Lep 20279	MG522395	BOLD:AAD2577	Tiroler Landesmuseum
Athrips pruinosella	LEALT057-16	TLMF Lep 20280	MG522433	BOLD:AAD2577	Tiroler Landesmuseum

Athrips pruinosella	PHLAJ290-14	KLM Lep 01905	MG522515	BOLD:AAD2577	Landesmuseum Kaernten
Athrips pruinosella	LEFIC321-10	MM03825	HM872165	BOLD:AAD2577	University of Oulu
Athrips pruinosella	LEFIA797-10	MM04151	HM386938	BOLD:AAD2577	University of Oulu
Athrips pruinosella	LEFIA798-10	MM04152	HM386939	BOLD:AAD2577	University of Oulu
Athrips pruinosella	PHLAJ294-14	KLM Lep 01909	MG522403	BOLD:AAD2577	Landesmuseum Kaernten
Autographa pulchrina	LEFIB391-10	MM01195	HM871290	BOLD:AAA3990	University of Oulu
Autographa pulchrina	LEFIF664-10	MM12614	HM875348	BOLD:AAA3990	University of Oulu
Autographa pulchrina	GBLGC325-12	BC ZSM Lep R 21655	MG522394	BOLD:AAA3990	SNSB, Zoologische Staatssammlung Muenchen
Autographa pulchrina	LEFID920-10	MM07382	HM873670	BOLD:AAA3990	University of Oulu
Autographa pulchrina	LEFIF658-10	MM12595	HM875342	BOLD:AAA3990	University of Oulu
Autographa pulchrina	FBLMV407-09	BC ZSM Lep 28387	GU707378	BOLD:AAA3990	Research Collection of Theo Gruenewald
Autographa pulchrina	LEFIG443-10	MM14481	HM876119	BOLD:AAA3990	University of Oulu
Autographa pulchrina	LEFIF172-10	MM10721	HM874866	BOLD:AAA3990	University of Oulu
Autographa pulchrina	GBLAA1705-15	BC ZSM Lep 80086	MG522240	BOLD:AAA3990	SNSB, Zoologische Staatssammlung Muenchen
Autographa pulchrina	LEFIB397-10	MM01201	HM871296	BOLD:AAA3990	University of Oulu
Autographa pulchrina	LEFIC625-10	MM04561	HM872446	BOLD:AAA3990	University of Oulu
Autographa pulchrina	LEFIE581-10	MM09480	HM874304	BOLD:AAA3990	University of Oulu
Autographa pulchrina	LEFIC601-10	MM04521	HM872422	BOLD:AAA3990	University of Oulu
Autographa pulchrina	FBLMV221-09	BC ZSM Lep 28201	HM391871	BOLD:AAA3990	SNSB, Zoologische Staatssammlung Muenchen
Autographa pulchrina	FBLMV215-09	BC ZSM Lep 28195	GU707313	BOLD:AAA3990	SNSB, Zoologische Staatssammlung Muenchen
Autographa pulchrina	LEFIH155-10	MM15092	HM876775	BOLD:AAA3990	University of Oulu
Autographa pulchrina	GWORO437-09	BC ZSM Lep 19792	GU688240	BOLD:AAA3990	SNSB, Zoologische Staatssammlung Muenchen
Autographa pulchrina	LEFIA949-10	MM13877	HM387081	BOLD:AAA3990	University of Oulu
Autographa pulchrina	LEALT549-16	TLMF Lep 20772	MG522245	BOLD:AAA3990	Tiroler Landesmuseum
Autographa pulchrina	LEFIA908-10	MM09786	HM387044	BOLD:AAA3990	University of Oulu
Autographa pulchrina	LEFIA199-10	MM01192	HM396544	BOLD:AAA3990	University of Oulu
Autographa pulchrina	LEFIC596-10	MM04516	HM872417	BOLD:AAA3990	University of Oulu
Autographa pulchrina	PHLAA704-09	TLMF Lep 00744	HM426101	BOLD:AAA3990	Tiroler Landesmuseum
Autographa pulchrina	PHLAA298-09	TLMF Lep 00338	HM425830	BOLD:AAA3990	Tiroler Landesmuseum

Autographa pulchrina	LEFIH158-10	MM15099	HM876778	BOLD:AAA3990	University of Oulu
Autographa pulchrina	LEFIH126-10	MM15022	HM876746	BOLD:AAA3990	University of Oulu
Autographa pulchrina	LEFIA862-10	MM09739	HM387000	BOLD:AAA3990	University of Oulu
Autographa pulchrina	LEFID658-10	MM06685	HM873420	BOLD:AAA3990	University of Oulu
Autographa pulchrina	LEFIA934-10	MM13862	HM387068	BOLD:AAA3990	University of Oulu
Autographa pulchrina	LEFIC603-10	MM04523	HM872424	BOLD:AAA3990	University of Oulu
Autographa pulchrina	LEFIB395-10	MM01199	HM871294	BOLD:AAA3990	University of Oulu
Autographa pulchrina	LEFIF171-10	MM10720	HM874865	BOLD:AAA3990	University of Oulu
Autographa pulchrina	LEFID962-10	MM07947	HM873712	BOLD:AAA3990	University of Oulu
Autographa pulchrina	LEFIH161-10	MM15105	HM876781	BOLD:AAA3990	University of Oulu
Autographa pulchrina	LEFIH129-10	MM15025	HM876749	BOLD:AAA3990	University of Oulu
Autographa pulchrina	LEFIF657-10	MM12594	HM875341	BOLD:AAA3990	University of Oulu
Autographa pulchrina	LEFIA909-10	MM09787	HM387045	BOLD:AAA3990	University of Oulu
Autographa pulchrina	PHLAA246-09	TLMF Lep 00286	HM425786	BOLD:AAA3990	Tiroler Landesmuseum
Autographa pulchrina	LEFIB389-10	MM01193	HM871288	BOLD:AAA3990	University of Oulu
Autographa pulchrina	LEFIC599-10	MM04519	HM872420	BOLD:AAA3990	University of Oulu
Autographa pulchrina	LEFIC595-10	MM04515	HM872416	BOLD:AAA3990	University of Oulu
Autographa pulchrina	LEFIH157-10	MM15094	HM876777	BOLD:AAA3990	University of Oulu
Autographa pulchrina	LEFIH147-10	MM15068	HM876767	BOLD:AAA3990	University of Oulu
Autographa pulchrina	LEFIC598-10	MM04518	HM872419	BOLD:AAA3990	University of Oulu
Autographa pulchrina	GWORR795-10	BC ZSM Lep 34001	HM904550	BOLD:AAA3990	SNSB, Zoologische Staatssammlung Muenchen
Autographa pulchrina	LEFIG453-10	MM14492	HM876129	BOLD:AAA3990	University of Oulu
Autographa pulchrina	PHLAA255-09	TLMF Lep 00295	HM425792	BOLD:AAA3990	Tiroler Landesmuseum
Autographa pulchrina	PHLAD087-11	TLMF Lep 03072	JN276672	BOLD:AAA3990	Tiroler Landesmuseum
Autographa pulchrina	LEFIA861-10	MM09738	HM386999	BOLD:AAA3990	University of Oulu
Autographa pulchrina	PHLAH820-12	TLMF Lep 08624	MG521952	BOLD:AAA3990	Tiroler Landesmuseum
Autographa pulchrina	LEFIE114-10	MM08303	HM873862	BOLD:AAA3990	University of Oulu
Autographa pulchrina	LEFIH148-10	MM15070	HM876768	BOLD:AAA3990	University of Oulu
Autographa pulchrina	LEFIJ1455-12	MM22723	KX048954	BOLD:AAA3990	University of Oulu
Autographa pulchrina	LEFIA193-10	MM01186	HM396538	BOLD:AAA3990	University of Oulu

Autographa pulchrina	LEFIC604-10	MM04524	HM872425	BOLD:AAA3990	University of Oulu
Autographa pulchrina	DEEUR552-15	TLMF Lep 17747	MG522402	BOLD:AAA3990	Research Collection of Peter Buchner
Autographa pulchrina	LEFIH153-10	MM15087	HM876773	BOLD:AAA3990	University of Oulu
Autographa pulchrina	GBLAB1844-14	BC ZSM Lep 77089	MG522703	BOLD:AAA3990	Research Collection of Alfred Haslberger
Autographa pulchrina	LEFIE869-10	MM10133	HM874587	BOLD:AAA3990	University of Oulu
Autographa pulchrina	GWORK553-09	BC ZSM Lep 21883	GU655892	BOLD:AAA3990	SNSB, Zoologische Staatssammlung Muenchen
Autographa pulchrina	LEFIB815-10	MM02751	HM871692	BOLD:AAA3990	University of Oulu
Autographa pulchrina	LEFIE120-10	MM08325	HM873868	BOLD:AAA3990	University of Oulu
Autographa pulchrina	PHLAC424-10	TLMF Lep 02459	JF860000	BOLD:AAA3990	Tiroler Landesmuseum
Autographa pulchrina	LEFIH127-10	MM15023	HM876747	BOLD:AAA3990	University of Oulu
Autographa pulchrina	GWORO837-09	BC ZSM Lep 30529	GU688504	BOLD:AAA3990	SNSB, Zoologische Staatssammlung Muenchen
Autographa pulchrina	LEFIH128-10	MM15024	HM876748	BOLD:AAA3990	University of Oulu
Autographa pulchrina	LEFIH152-10	MM15086	HM876772	BOLD:AAA3990	University of Oulu
Autographa pulchrina	PHLAB1218-10	TLMF Lep 02018	HQ968428	BOLD:AAA3990	Tiroler Landesmuseum
Autographa pulchrina	LEFIF175-10	MM10724	HM874869	BOLD:AAA3990	University of Oulu
Autographa pulchrina	LEFIH150-10	MM15075	HM876770	BOLD:AAA3990	University of Oulu
Autographa pulchrina	LEFIH151-10	MM15085	HM876771	BOLD:AAA3990	University of Oulu
Autographa pulchrina	LEALT548-16	TLMF Lep 20771	MG522379	BOLD:AAA3990	Tiroler Landesmuseum
Autographa pulchrina	LEFIE868-10	MM10127	HM874586	BOLD:AAA3990	University of Oulu
Autographa pulchrina	LEFIC597-10	MM04517	HM872418	BOLD:AAA3990	University of Oulu
Autographa pulchrina	LEFIB398-10	MM01202	HM871297	BOLD:AAA3990	University of Oulu
Autographa pulchrina	LEFIC602-10	MM04522	HM872423	BOLD:AAA3990	University of Oulu
Autographa pulchrina	LEFIB814-10	MM02750	HM871691	BOLD:AAA3990	University of Oulu
Autographa pulchrina	LEATB292-13	TLMF Lep 10469	MG522187	BOLD:AAA3990	Naturmuseum Suedtirol
Autographa pulchrina	LEFIB393-10	MM01197	HM871292	BOLD:AAA3990	University of Oulu
Autographa pulchrina	GBLAA914-14	BC ZSM Lep 83475	MG522207	BOLD:AAA3990	SNSB, Zoologische Staatssammlung Muenchen
Autographa pulchrina	LEFID852-10	MM06978	HM873609	BOLD:AAA3990	University of Oulu
Autographa pulchrina	LEFIA196-10	MM01189	HM396541	BOLD:AAA3990	University of Oulu
Autographa pulchrina	LEFIC605-10	MM04525	HM872426	BOLD:AAA3990	University of Oulu
Autographa pulchrina	LEFIE174-10	MM08447	HM873920	BOLD:AAA3990	University of Oulu

Autographa pulchrina	LEFIB390-10	MM01194	HM871289	BOLD:AAA3990	University of Oulu
Autographa pulchrina	LEFIF168-10	MM10717	HM874862	BOLD:AAA3990	University of Oulu
Autographa pulchrina	LEFIB396-10	MM01200	HM871295	BOLD:AAA3990	University of Oulu
Autographa pulchrina	LEFIG201-10	MM14084	HM875881	BOLD:AAA3990	University of Oulu
Autographa pulchrina	LEFIF170-10	MM10719	HM874864	BOLD:AAA3990	University of Oulu
Autographa pulchrina	LEFID919-10	MM07381	HM873669	BOLD:AAA3990	University of Oulu
Autographa pulchrina	LEFIB394-10	MM01198	HM871293	BOLD:AAA3990	University of Oulu
Autographa pulchrina	LEFIA198-10	MM01191	HM396543	BOLD:AAA3990	University of Oulu
Autographa pulchrina	LEFID041-10	MM05503	HM872855	BOLD:AAA3990	University of Oulu
Autographa pulchrina	LEFIA195-10	MM01188	HM396540	BOLD:AAA3990	University of Oulu
Autographa pulchrina	LEFIH154-10	MM15091	HM876774	BOLD:AAA3990	University of Oulu
Autographa pulchrina	LEFIE121-10	MM08326	HM873869	BOLD:AAA3990	University of Oulu
Autographa pulchrina	LEALT550-16	TLMF Lep 20773	MG522769	BOLD:AAA3990	Tiroler Landesmuseum
Autographa pulchrina	LEFIH156-10	MM15093	HM876776	BOLD:AAA3990	University of Oulu
Autographa pulchrina	LEFIA948-10	MM13876	HM387080	BOLD:AAA3990	University of Oulu
Autographa pulchrina	LEFID946-10	MM07759	HM873696	BOLD:AAA3990	University of Oulu
Autographa pulchrina	LEFID657-10	MM06684	HM873419	BOLD:AAA3990	University of Oulu
Autographa pulchrina	LEFIB392-10	MM01196	HM871291	BOLD:AAA3990	University of Oulu
Autographa pulchrina	FBLMV216-09	BC ZSM Lep 28196	HM391869	BOLD:AAA3990	SNSB, Zoologische Staatssammlung Muenchen
Autographa pulchrina	PHLAE213-11	TLMF Lep 04433	JN276673	BOLD:AAA3990	Tiroler Landesmuseum
Autographa pulchrina	LEFID050-10	MM05524	HM872864	BOLD:AAA3990	University of Oulu
Autographa pulchrina	LEFIE175-10	MM08448	HM873921	BOLD:AAA3990	University of Oulu
Autographa pulchrina	PHLAD086-11	TLMF Lep 03071	JN276671	BOLD:AAA3990	Tiroler Landesmuseum
Autographa pulchrina	GWOTL273-13	BC ZSM Lep 67299	MG522836	BOLD:AAA3990	SNSB, Zoologische Staatssammlung Muenchen
Boloria dia	GBLAA349-14	BC ZSM Lep 80345	MG522336	BOLD:AAB5849	SNSB, Zoologische Staatssammlung Muenchen
Boloria dia	LEALT532-16	TLMF Lep 20755	MG522078	BOLD:AAB5849	Tiroler Landesmuseum
Boloria dia	FBLMV675-09	BC ZSM Lep 28655	GU707295	BOLD:AAB5849	SNSB, Zoologische Staatssammlung Muenchen
Boloria dia	LEALT560-16	TLMF Lep 20783	MG522224	BOLD:AAB5849	Tiroler Landesmuseum
Boloria dia	EZROM850-08	RVcoll.06-N012	HQ004072	BOLD:AAB5849	Institut de Biologia Evolutiva (CSIC-UPF)
Boloria dia	EZROM851-08	RVcoll.07-C423	HQ004073	BOLD:AAB5849	Institut de Biologia Evolutiva (CSIC-UPF)

Boloria dia	LOWA774-06	2005-LOWA-774	FJ663360	BOLD:AAB5849	McGuire Centre for Lepidoptera and Biodiversity
Boloria dia	LOWA090-06	2005-LOWA-90	FJ663363	BOLD:AAB5849	McGuire Centre for Lepidoptera and Biodiversity
Boloria dia	EZROM854-08	RVcoll.07-D909	HQ004078	BOLD:AAB5849	Institut de Biologia Evolutiva (CSIC-UPF)
Boloria dia	LOWA775-06	2005-LOWA-775	FJ663359	BOLD:AAB5849	McGuire Centre for Lepidoptera and Biodiversity
Boloria dia	GBLAF769-14	BC ZSM Lep 82855	MG522815	BOLD:AAB5849	SNSB, Zoologische Staatssammlung Muenchen
Boloria dia	EZROM093-08	RV-06-M957	HQ004076	BOLD:AAB5849	Institut de Biologia Evolutiva (CSIC-UPF)
Boloria dia	PHLAH474-12	TLMF Lep 08293	KP253455	BOLD:AAB5849	inatura, Dornbirn
Boloria dia	GBLAB321-13	BC ZSM Lep 72622	MG522152	BOLD:AAB5849	SNSB, Zoologische Staatssammlung Muenchen
Boloria dia	EZROM091-08	RV-06-K536	HQ004074	BOLD:AAB5849	Institut de Biologia Evolutiva (CSIC-UPF)
Boloria dia	EZROM853-08	RVcoll.07-D562	HQ004080	BOLD:AAB5849	Institut de Biologia Evolutiva (CSIC-UPF)
Boloria dia	LEATI047-15	TLMF Lep 17432	MG522744	BOLD:AAB5849	Tiroler Landesmuseum
Boloria dia	LOWA091-06	2005-LOWA-91	FJ663362	BOLD:AAB5849	McGuire Centre for Lepidoptera and Biodiversity
Boloria dia	GBLAD222-14	BC ZSM Lep 78793	MG522518	BOLD:AAB5849	SNSB, Zoologische Staatssammlung Muenchen
Boloria dia	EZROM852-08	RVcoll.07-D504	HQ004079	BOLD:AAB5849	Institut de Biologia Evolutiva (CSIC-UPF)
Boloria dia	LEALT533-16	TLMF Lep 20756	MG522002	BOLD:AAB5849	Tiroler Landesmuseum
Boloria dia	LEATG056-14	TLMF Lep 13843	MG522374	BOLD:AAB5849	Tiroler Landesmuseum
Boloria dia	EZROM094-08	RV-07-C954	HQ004077	BOLD:AAB5849	Institut de Biologia Evolutiva (CSIC-UPF)
Boloria dia	GBLAA1377-15	BC ZSM Lep 87073	MG522702	BOLD:AAB5849	SNSB, Zoologische Staatssammlung Muenchen
Boloria dia	GWORR671-10	BC ZSM Lep 32167	JF415680	BOLD:AAB5849	SNSB, Zoologische Staatssammlung Muenchen
Boloria dia	EZROM855-08	RVcoll.07-D922	HQ004081	BOLD:AAB5849	Institut de Biologia Evolutiva (CSIC-UPF)
Boloria dia	LOWA092-06	2005-LOWA-92	FJ663361	BOLD:AAB5849	McGuire Centre for Lepidoptera and Biodiversity
Boloria dia	EZROM092-08	RV-06-K538	HQ004075	BOLD:AAB5849	Institut de Biologia Evolutiva (CSIC-UPF)
Boloria dia	EZROM849-08	RVcoll.08-M354	HQ004071	BOLD:AAB5849	Institut de Biologia Evolutiva (CSIC-UPF)
Boloria napaea	LOWA148-06	2005-LOWA-148	FJ663334	BOLD:AAA9406	McGuire Centre for Lepidoptera and Biodiversity
Boloria napaea	LEATH794-14	TLMF Lep 16006	MG522777	BOLD:AAA9406	Tiroler Landesmuseum
Boloria napaea	LEFIJ543-10	MM17168	JN272537	BOLD:AAA9406	University of Oulu
Boloria napaea	LEALT296-16	TLMF Lep 20519	MG522510	BOLD:AAA9406	Tiroler Landesmuseum
Boloria napaea	LEALT295-16	TLMF Lep 20518	MG522516	BOLD:AAA9406	Tiroler Landesmuseum
Boloria napaea	PHLAH355-12	TLMF Lep 07794	MG522452	BOLD:AAA9406	Tiroler Landesmuseum
Boloria napaea	LEATG041-14	TLMF Lep 13828	MG522437	BOLD:AAA9406	Tiroler Landesmuseum

Boloria napaea	LEFIC094-10	MM03407	HQ570285	BOLD:AAA9406	University of Oulu
Boloria napaea	LOWA204-06	2005-LOWA-204	FJ663332	BOLD:AAA9406	McGuire Centre for Lepidoptera and Biodiversity
Boloria napaea	LOWA149-06	2005-LOWA-149	FJ663333	BOLD:AAA9406	McGuire Centre for Lepidoptera and Biodiversity
Boloria napaea	LEALT294-16	TLMF Lep 20517	MG522409	BOLD:AAA9406	Tiroler Landesmuseum
Boloria napaea	LEATC570-13	TLMF Lep 12552	KM572854	BOLD:AAA9406	Tiroler Landesmuseum
Boloria napaea	PHLSA407-11	TLMF Lep 05862	MG522304	BOLD:AAA9406	Tiroler Landesmuseum
Boloria napaea	LEFIA746-10	MM04100	HM386890	BOLD:AAA9406	University of Oulu
Boloria napaea	PHLAH356-12	TLMF Lep 07795	MG522031	BOLD:AAA9406	Tiroler Landesmuseum
Boloria titania	LEALT380-16	TLMF Lep 20603	MG522502	BOLD:AAA2067	Tiroler Landesmuseum
Boloria titania	EZROM804-08	RVcoll.08-M602	HQ004109	BOLD:ACS2433	Institut de Biologia Evolutiva (CSIC-UPF)
Boloria titania	PHLAI487-13	TLMF Lep 09049	MG522254	BOLD:ACS2433	Tiroler Landesmuseum
Boloria titania	FBLMX197-11	BC ZSM Lep 50408	KP870710	BOLD:ACS2433	Research Collection of Alfred Haslberger
Boloria titania	LEATF453-14	TLMF Lep 13765	MG522062	BOLD:ACS2433	Tiroler Landesmuseum
Boloria titania	LOWA218-06	2005-LOWA-218	FJ663378	BOLD:AAA2067	McGuire Centre for Lepidoptera and Biodiversity
Boloria titania	FBLMU472-09	BC ZSM Lep 27122	HM391822	BOLD:ACS2433	SNSB, Zoologische Staatssammlung Muenchen
Boloria titania	PHLAA705-09	TLMF Lep 00745	HM426102	BOLD:ACS2433	Tiroler Landesmuseum
Boloria titania	EZROM806-08	RVcoll.08-M604	HQ004111	BOLD:ACS2433	Institut de Biologia Evolutiva (CSIC-UPF)
Boloria titania	LOWA219-06	2005-LOWA-219	FJ663377	BOLD:AAA2067	McGuire Centre for Lepidoptera and Biodiversity
Boloria titania	PHLAB1033-10	TLMF Lep 01833	HQ968296	BOLD:ACS2433	Tiroler Landesmuseum
Boloria titania	EZROM805-08	RVcoll.08-M603	HQ004110	BOLD:ACS2433	Institut de Biologia Evolutiva (CSIC-UPF)
Boloria titania	GWORR672-10	BC ZSM Lep 32168	JF415689	BOLD:ACS2433	SNSB, Zoologische Staatssammlung Muenchen
Boloria titania	PHLAF622-11	TLMF Lep 05792	MG522376	BOLD:ACS2433	Tiroler Landesmuseum
Boloria titania	EZROM803-08	RVcoll.08-M601	HQ004108	BOLD:ACS2433	Institut de Biologia Evolutiva (CSIC-UPF)
Boloria titania	LEALT375-16	TLMF Lep 20598	MG522392	BOLD:AAA2067	Tiroler Landesmuseum
Boloria titania	GWORA2496-09	BC ZSM Lep 30708	HM393214	BOLD:ACS2433	Research Collection of Ralph Sturm
Boloria titania	LEATD455-13	TLMF Lep 13102	MG521930	BOLD:ACS2433	Tiroler Landesmuseum
Boloria titania	PHLAA681-09	TLMF Lep 00721	HM426082	BOLD:ACS2433	Tiroler Landesmuseum
Boloria titania	PHLAB1034-10	TLMF Lep 01834	HQ968297	BOLD:ACS2433	Tiroler Landesmuseum
Boloria titania	PHLAB1035-10	TLMF Lep 01835	HQ968298	BOLD:ACS2433	Tiroler Landesmuseum
Boloria titania	LEALT376-16	TLMF Lep 20599	MG522604	BOLD:AAA2067	Tiroler Landesmuseum

Boloria titania	PHLAB293-10	TLMF Lep 01093	HQ968460	BOLD:ACS2433	Tiroler Landesmuseum
Boloria titania	EZROM055-08	RV-06-N010	HQ004107	BOLD:ACS2433	Institut de Biologia Evolutiva (CSIC-UPF)
Boloria titania	LEATF455-14	TLMF Lep 13767	MG522697	BOLD:ACS2433	Tiroler Landesmuseum
Boloria titania	EZRMN428-09	RVcoll.09-V681	HQ004106	BOLD:ACS2433	Institut de Biologia Evolutiva (CSIC-UPF)
Brenthis ino	LEFIJ542-10	MM17167	JF853659	BOLD:AAA9312	University of Oulu
Brenthis ino	PHLAI546-13	TLMF Lep 09108	MG522679	BOLD:AAA9312	Tiroler Landesmuseum
Brenthis ino	LOWA550-06	2005-LOWA-550	FJ663338	BOLD:AAA9312	McGuire Centre for Lepidoptera and Biodiversity
Brenthis ino	PHLAW007-13	TLMF Lep 09804	KM572384	BOLD:AAA9312	Tiroler Landesmuseum
Brenthis ino	GBLAB322-13	BC ZSM Lep 72623	MG522453	BOLD:AAA9312	SNSB, Zoologische Staatssammlung Muenchen
Brenthis ino	LOWA193-06	2005-LOWA-193	FJ663340	BOLD:AAA9312	McGuire Centre for Lepidoptera and Biodiversity
Brenthis ino	LEATG009-14	TLMF Lep 13796	MG522564	BOLD:AAA9312	Tiroler Landesmuseum
Brenthis ino	EZROM815-08	RVcoll.07-D309	HQ004132	BOLD:AAA9312	Institut de Biologia Evolutiva (CSIC-UPF)
Brenthis ino	LEATF460-14	TLMF Lep 13772	MG522099	BOLD:AAA9312	Tiroler Landesmuseum
Brenthis ino	EZROM814-08	RVcoll.06-M899	HQ004131	BOLD:AAA9312	Institut de Biologia Evolutiva (CSIC-UPF)
Brenthis ino	EZROM065-08	RV-07-D345	HQ004134	BOLD:AAA9312	Institut de Biologia Evolutiva (CSIC-UPF)
Brenthis ino	LEALT428-16	TLMF Lep 20651	MG522726	BOLD:AAA9312	Tiroler Landesmuseum
Brenthis ino	LEATJ1246-16	TLMF Lep 19569	MG522621	BOLD:AAA9312	Tiroler Landesmuseum
Brenthis ino	LEATG514-14	TLMF Lep 14301	MG522707	BOLD:AAA9312	Naturmuseum Suedtirol
Brenthis ino	EZROM063-08	RV-06-M865	HQ004136	BOLD:AAA9312	Institut de Biologia Evolutiva (CSIC-UPF)
Brenthis ino	EZROM817-08	RVcoll.07-D346	HQ004130	BOLD:AAA9312	Institut de Biologia Evolutiva (CSIC-UPF)
Brenthis ino	LOWA194-06	2005-LOWA-194	FJ663339	BOLD:AAA9312	McGuire Centre for Lepidoptera and Biodiversity
Brenthis ino	GWORA2493-09	BC ZSM Lep 30705	HM393212	BOLD:AAA9312	Research Collection of Ralph Sturm
Brenthis ino	LEFIC069-10	MM03354	HM871938	BOLD:AAA9312	University of Oulu
Brenthis ino	EZROM816-08	RVcoll.07-D318	HQ004129	BOLD:AAA9312	Institut de Biologia Evolutiva (CSIC-UPF)
Brenthis ino	GBLAF776-14	BC ZSM Lep 82862	MG522629	BOLD:AAA9312	SNSB, Zoologische Staatssammlung Muenchen
Brenthis ino	LEFIC068-10	MM03353	HM871937	BOLD:AAA9312	University of Oulu
Brenthis ino	LOWA551-06	2005-LOWA-551	FJ663337	BOLD:AAA9312	McGuire Centre for Lepidoptera and Biodiversity
Brenthis ino	EZROM807-08	RVcoll.08-M478	HQ004133	BOLD:AAA9312	Institut de Biologia Evolutiva (CSIC-UPF)
Brenthis ino	GWORR666-10	BC ZSM Lep 32162	JF415690	BOLD:AAA9312	SNSB, Zoologische Staatssammlung Muenchen
Brenthis ino	EZROM064-08	RV-06-M906	HQ004135	BOLD:AAA9312	Institut de Biologia Evolutiva (CSIC-UPF)

Brenthis ino	LEALT426-16	TLMF Lep 20649	MG522806	BOLD:AAA9312	Tiroler Landesmuseum
Brenthis ino	LEALT427-16	TLMF Lep 20650	MG522609	BOLD:AAA9312	Tiroler Landesmuseum
Carsia sororiata	PHLAC761-10	TLMF Lep 02796	KX042145	BOLD:AAC1640	Tiroler Landesmuseum
Carsia sororiata	LEFIB866-10	MM02919	HM871743	BOLD:AAC1640	University of Oulu
Carsia sororiata	LEALT496-16	TLMF Lep 20719	MG522490	BOLD:AAC1640	Tiroler Landesmuseum
Carsia sororiata	LEALT495-16	TLMF Lep 20718	MG522305	BOLD:AAC1640	Tiroler Landesmuseum
Carsia sororiata	LEALT494-16	TLMF Lep 20717	MG521950	BOLD:AAC1640	Tiroler Landesmuseum
Carsia sororiata	LEATC568-13	TLMF Lep 12550	KM573561	BOLD:AAC1640	Tiroler Landesmuseum
Carsia sororiata	ABOLB271-15	TLMF Lep 17276	MG522704	BOLD:AAC1640	Tiroler Landesmuseum
Carsia sororiata	NOLEP060-14	BCZMBNLep0060	MG522118	BOLD:AAC1640	University of Bergen, Natural History Collections
Carsia sororiata	LEATF419-14	TLMF Lep 13731	MG522547	BOLD:AAC1640	Tiroler Landesmuseum
Carsia sororiata	LEFIE036-10	MM08117	HM873785	BOLD:AAC1640	University of Oulu
Carsia sororiata	LEFIA381-10	MM01438	HM386723	BOLD:AAC1640	University of Oulu
Carsia sororiata	PHLAC760-10	TLMF Lep 02795	JF860293	BOLD:AAC1640	Tiroler Landesmuseum
Carsia sororiata	NOLEP068-14	BCZMBNLep0068	MG522101	BOLD:AAC1640	University of Bergen, Natural History Collections
Carsia sororiata	PHLAC676-10	TLMF Lep 02711	JF860238	BOLD:AAC1640	Tiroler Landesmuseum
Caryocolum leucomelanella	PHLAF105-11	TLMF Lep 05275	KJ427059	BOLD:AAM3503	Tiroler Landesmuseum
Caryocolum leucomelanella	ABOLA937-15	TLMF Lep 16897	MG521942	BOLD:AAM3503	Tiroler Landesmuseum
Caryocolum leucomelanella	PHLAF100-11	TLMF Lep 05270	KJ427073	BOLD:AAM3503	Tiroler Landesmuseum
Caryocolum leucomelanella	LEATJ1462-16	TLMF Lep 19785	MG522633	BOLD:AAM3503	Tiroler Landesmuseum
Caryocolum leucomelanella	LASTS837-15	TLMF Lep 16702	MG522541	BOLD:AAM3503	Tiroler Landesmuseum
Caryocolum leucomelanella	LEALT017-16	TLMF Lep 20240	MG522281	BOLD:AAM3503	Tiroler Landesmuseum
Caryocolum leucomelanella	LEATG364-14	TLMF Lep 14151	MG522005	BOLD:AAM3503	Tiroler Landesmuseum
Caryocolum leucomelanella	LASTS836-15	TLMF Lep 16701	MG522200	BOLD:AAM3503	Tiroler Landesmuseum
Caryocolum leucomelanella	ABOLA936-15	TLMF Lep 16896	MG521985	BOLD:AAM3503	Tiroler Landesmuseum
Caryocolum leucomelanella	PHLAD578-11	TLMF Lep 03753	JN271012	BOLD:AAM3503	Tiroler Landesmuseum
Caryocolum leucomelanella	ABOLB203-15	TLMF Lep 17208	MG522497	BOLD:AAM3503	Tiroler Landesmuseum
Caryocolum leucomelanella	PHLAB830-10	TLMF Lep 01630	HQ968834	BOLD:AAM3503	Tiroler Landesmuseum
Caryocolum leucomelanella	LEATE421-13	TLMF Lep 11833	KJ427051	BOLD:AAM3503	Tiroler Landesmuseum
Caryocolum leucomelanella	PHLAB829-10	TLMF Lep 01629	HQ968833	BOLD:AAM3503	Tiroler Landesmuseum

Caryocolum leucomelanella	LASTS808-15	TLMF Lep 16673	MG521943	BOLD:AAM3503	Tiroler Landesmuseum
Caryocolum leucomelanella	LEALT016-16	TLMF Lep 20239	MG522059	BOLD:AAM3503	Tiroler Landesmuseum
Caryocolum leucomelanella	LEALT018-16	TLMF Lep 20241	MG522206	BOLD:AAM3503	Tiroler Landesmuseum
Caryocolum pullatella	LEALT026-16	TLMF Lep 20249	MG522462	BOLD:AAC1599	Tiroler Landesmuseum
Caryocolum pullatella	PHLAF102-11	TLMF Lep 05272	JX034621	BOLD:AAC1598	Tiroler Landesmuseum
Caryocolum pullatella	PHLAF103-11	TLMF Lep 05273	JX034655	BOLD:AAC1598	Tiroler Landesmuseum
Caryocolum pullatella	LEATJ138-15	TLMF Lep 18568	MG522669	BOLD:AAC1598	Tiroler Landesmuseum
Caryocolum pullatella	LEALT027-16	TLMF Lep 20250	MG522587	BOLD:AAC1599	Tiroler Landesmuseum
Caryocolum pullatella	LEFIG527-10	MM14646	HM876200	BOLD:AAC1599	University of Oulu
Caryocolum pullatella	PHLAC841-10	TLMF Lep 02876	JF860338	BOLD:AAC1598	Tiroler Landesmuseum
Caryocolum pullatella	LEFIB936-10	MM03085	HM871813	BOLD:AAC1599	University of Oulu
Caryocolum pullatella	PHLAC838-10	TLMF Lep 02873	KJ427028	BOLD:AAC1598	Tiroler Landesmuseum
Caryocolum pullatella	PHLAF106-11	TLMF Lep 05276	JX034697	BOLD:AAC1598	Tiroler Landesmuseum
Caryocolum pullatella	LEFIE429-10	MM09053	HM874153	BOLD:AAC1599	University of Oulu
Caryocolum pullatella	LEFIB286-10	MM00778	HM871187	BOLD:AAC1599	University of Oulu
Catoptria languidellus	PHLAB779-10	TLMF Lep 01579	HQ968787	BOLD:AAO4293	Tiroler Landesmuseum
Catoptria languidellus	LEATG055-14	TLMF Lep 13842	MG522090	BOLD:AAO4293	Tiroler Landesmuseum
Catoptria languidellus	PHLAB778-10	TLMF Lep 01578	HQ968786	BOLD:AAO4293	Tiroler Landesmuseum
Catoptria languidellus	PHLAG100-12	TLMF Lep 06410	MG522714	BOLD:AAO4293	Tiroler Landesmuseum
Catoptria languidellus	PHLAF207-11	TLMF Lep 05377	MG521965	BOLD:AAO4293	Tiroler Landesmuseum
Catoptria languidellus	LEALT033-16	TLMF Lep 20256	MG522580	BOLD:AAO4293	Tiroler Landesmuseum
Catoptria languidellus	LEALT032-16	TLMF Lep 20255	MG522216	BOLD:AAO4293	Tiroler Landesmuseum
Catoptria languidellus	LEALT031-16	TLMF Lep 20254	MG522335	BOLD:AAO4293	Tiroler Landesmuseum
Catoptria languidellus	PHLAF206-11	TLMF Lep 05376	MG522266	BOLD:AAO4293	Tiroler Landesmuseum
Celypha rivulana	LEALT038-16	TLMF Lep 20261	MG521984	BOLD:AAF2534	Tiroler Landesmuseum
Celypha rivulana	LEALT036-16	TLMF Lep 20259	MG522230	BOLD:AAF2534	Tiroler Landesmuseum
Celypha rivulana	LASTS078-14	TLMF Lep 14530	MG522294	BOLD:AAF2534	Tiroler Landesmuseum
Celypha rivulana	LEATG652-14	TLMF Lep 14439	MG522675	BOLD:AAF2534	Tiroler Landesmuseum
Celypha rivulana	LASTS074-14	TLMF Lep 14526	MG522390	BOLD:AAF2534	Tiroler Landesmuseum
Celypha rivulana	LEFIB900-10	MM03001	HM871777	BOLD:AAF2534	University of Oulu

Celypha rivulana	LEATE769-13	TLMF Lep 12181	MG522327	BOLD:AAF2534	Tiroler Landesmuseum
Celypha rivulana	LEFIF886-10	MM13292	HM875568	BOLD:AAF2534	University of Oulu
Celypha rivulana	LEFIB494-10	MM02072	HM871389	BOLD:AAF2534	University of Oulu
Celypha rivulana	PHLAV201-12	TLMF Lep 08020	KM573271	BOLD:AAF2534	inatura, Dornbirn
Cerapteryx graminis	LEALT509-16	TLMF Lep 20732	MG522326	BOLD:AAB4284	Tiroler Landesmuseum
Cerapteryx graminis	GWOSC413-10	BC ZSM Lep 36089	HQ566459	BOLD:AAB4284	SNSB, Zoologische Staatssammlung Muenchen
Cerapteryx graminis	FBLMV074-09	BC ZSM Lep 28054	GU707453	BOLD:AAB4284	SNSB, Zoologische Staatssammlung Muenchen
Cerapteryx graminis	GBLAD130-14	BC ZSM Lep 78701	MG522690	BOLD:AAB4284	SNSB, Zoologische Staatssammlung Muenchen
Cerapteryx graminis	LEFIA528-10	MM01612	KM572110	BOLD:AAB4284	University of Oulu
Cerapteryx graminis	GWOTL094-13	BC ZSM Lep 67025	MG522595	BOLD:AAB4284	SNSB, Zoologische Staatssammlung Muenchen
Cerapteryx graminis	LEALT510-16	TLMF Lep 20733	MG522252	BOLD:AAB4284	Tiroler Landesmuseum
Cerapteryx graminis	PHLAC593-10	TLMF Lep 02628	JF860160	BOLD:AAB4284	Tiroler Landesmuseum
Cerapteryx graminis	GBLAF282-14	BC ZSM Lep 81988	MG522280	BOLD:AAB4284	SNSB, Zoologische Staatssammlung Muenchen
Cerapteryx graminis	LEFIE021-10	MM08079	HM873770	BOLD:AAB4284	University of Oulu
Cerapteryx graminis	LEALT449-16	TLMF Lep 20672	MG522375	BOLD:AAB4284	Tiroler Landesmuseum
Cerapteryx graminis	LEFIB299-10	MM00812	HM871200	BOLD:AAB4284	University of Oulu
Cerapteryx graminis	LEFIA529-10	MM01613	KM573093	BOLD:AAB4284	University of Oulu
Cerapteryx graminis	LEATG313-14	TLMF Lep 14100	MG522258	BOLD:AAB4284	Tiroler Landesmuseum
Cerapteryx graminis	LEATG312-14	TLMF Lep 14099	MG522381	BOLD:AAB4284	Tiroler Landesmuseum
Cerapteryx graminis	GWORK481-09	BC ZSM Lep 21811	JF415507	BOLD:AAB4284	SNSB, Zoologische Staatssammlung Muenchen
Cerapteryx graminis	LEATB539-13	TLMF Lep 10716	MG522822	BOLD:AAB4284	Naturmuseum Suedtirol
Cerapteryx graminis	GBLAA1107-15	BC ZSM Lep 86613	MG522075	BOLD:AAB4284	SNSB, Zoologische Staatssammlung Muenchen
Cerapteryx graminis	GBLAA1002-14	BC ZSM Lep 83658	MG522552	BOLD:AAB4284	SNSB, Zoologische Staatssammlung Muenchen
Cerapteryx graminis	GBLAA1740-15	BC ZSM Lep 79741	MG521964	BOLD:AAB4284	SNSB, Zoologische Staatssammlung Muenchen
Cerapteryx graminis	NOLEP037-14	BCZMBNLep0037	MG522413	BOLD:AAB4284	University of Bergen, Natural History Collections
Cerapteryx graminis	PHLAE302-11	TLMF Lep 04617	KM573410	BOLD:AAB4284	Tiroler Landesmuseum
Charissa ambiguata	LEALT416-16	TLMF Lep 20639	MG522427	BOLD:ACE4202	Tiroler Landesmuseum
Charissa ambiguata	LEALT537-16	TLMF Lep 20760	MG522169	BOLD:ACE4202	Tiroler Landesmuseum
Charissa ambiguata	LEATD226-13	TLMF Lep 12873	MG522829	BOLD:ACF4447	Tiroler Landesmuseum
Charissa ambiguata	LEATB639-13	TLMF Lep 10816	MG522321	BOLD:ACF4447	Naturmuseum Suedtirol

Charissa ambiguata	PHLAV353-12	TLMF Lep 08172	KP253664	BOLD:ACF4447	inatura, Dornbirn
Charissa ambiguata	PHLAA350-09	TLMF Lep 00390	HM425876	BOLD:ACF4447	Tiroler Landesmuseum
Charissa ambiguata	LEATD159-13	TLMF Lep 12806	MG522323	BOLD:ACF4447	Tiroler Landesmuseum
Charissa ambiguata	PHLSA712-11	TLMF Lep 06167	KP253347	BOLD:ACF4447	Tiroler Landesmuseum
Charissa ambiguata	GWORB2997-08	BC ZSM Lep 01775	HQ601102	BOLD:ACF4447	SNSB, Zoologische Staatssammlung Muenchen
Charissa ambiguata	LEATD194-13	TLMF Lep 12841	MG521932	BOLD:ACE4202	Tiroler Landesmuseum
Charissa ambiguata	PHLAC397-10	TLMF Lep 02432	JF859973	BOLD:ACF4447	Tiroler Landesmuseum
Charissa ambiguata	LEATB624-13	TLMF Lep 10801	MG521939	BOLD:ACF4447	Naturmuseum Suedtirol
Charissa ambiguata	PHLAA269-09	TLMF Lep 00309	HM425806	BOLD:ACF4447	Tiroler Landesmuseum
Charissa ambiguata	GWORM009-09	BC ZSM Lep 24058	GU687332	BOLD:ACF4447	Research Collection of Alfred Haslberger
Chionodes distinctella	LEALT111-16	TLMF Lep 20334	MG522212	BOLD:AAE9034	Tiroler Landesmuseum
Chionodes distinctella	LEALT204-16	TLMF Lep 20427	MG522292	BOLD:AAE9034	Tiroler Landesmuseum
Chionodes distinctella	LEALT110-16	TLMF Lep 20333	MG522111	BOLD:AAE9034	Tiroler Landesmuseum
Chionodes distinctella	LEALT205-16	TLMF Lep 20428	MG522455	BOLD:AAE9034	Tiroler Landesmuseum
Chionodes distinctella	LEALT206-16	TLMF Lep 20429	MG522284	BOLD:AAE9034	Tiroler Landesmuseum
Chionodes distinctella	PHLAJ040-13	KLM Lep 00800	MG521963	BOLD:AAE9034	Landesmuseum Kaernten
Chionodes distinctella	ABOLA298-14	TLMF Lep 15320	MG522660	BOLD:AAE9034	Tiroler Landesmuseum
Chionodes distinctella	ABOLB184-15	TLMF Lep 17189	MG522361	BOLD:AAE9034	Tiroler Landesmuseum
Chionodes distinctella	ABOLA377-14	TLMF Lep 15399	MG522432	BOLD:AAE9034	Tiroler Landesmuseum
Chionodes distinctella	LEFIE796-10	MM09981	HM874515	BOLD:AAE9034	University of Oulu
Chionodes distinctella	LEATJ1443-16	TLMF Lep 19766	MG522479	BOLD:AAE9034	Tiroler Landesmuseum
Chionodes distinctella	LEFIJ234-10	MM13548	KM373634	BOLD:AAE9034	University of Oulu
Chionodes distinctella	LEFIE939-10	MM10327	HM874656	BOLD:AAE9034	University of Oulu
Chionodes distinctella	LEFIE821-10	MM10027	HM874540	BOLD:AAE9035	University of Oulu
Chionodes distinctella	LEFIJ1289-11	MM21149	KT782352	BOLD:AAE9034	University of Oulu
Chionodes distinctella	PHLAE094-11	TLMF Lep 04314	JN271074	BOLD:AAE9034	Tiroler Landesmuseum
Chionodes distinctella	LEATH338-14	TLMF Lep 15550	MG522248	BOLD:AAE9034	Tiroler Landesmuseum
Chionodes distinctella	PHLAJ039-13	KLM Lep 00799	MG522360	BOLD:AAE9034	Landesmuseum Kaernten
Chionodes distinctella	LEFIJ249-10	MM14533	JF853492	BOLD:AAE9034	University of Oulu
Chionodes distinctella	NLLEA656-12	RMNH.INS.540851	KX048385	BOLD:AAE9034	Naturalis Biodiversity Centre

Chionodes distinctella	ABOLA807-15	TLMF Lep 16767	MG522628	BOLD:AAE9034	Tiroler Landesmuseum
Chionodes distinctella	LEFIJ146-10	MM10028	JF853459	BOLD:AAE9035	University of Oulu
Chionodes distinctella	PHLAF135-11	TLMF Lep 05305	MG522569	BOLD:AAE9034	Tiroler Landesmuseum
Chionodes distinctella	LEFIJ157-10	MM10343	JF853470	BOLD:AAE9034	University of Oulu
Chionodes distinctella	LEFID565-10	MM06558	HM873330	BOLD:AAE9034	University of Oulu
Chionodes distinctella	LEATE780-13	TLMF Lep 12192	MG522000	BOLD:AAE9034	Tiroler Landesmuseum
Chionodes distinctella	PHLAJ020-13	KLM Lep 00780	MG522057	BOLD:AAE9034	Landesmuseum Kaernten
Chionodes distinctella	NLLEA401-12	RMNH.INS.539016	KX048142	BOLD:AAE9034	Naturalis Biodiversity Centre
Chionodes distinctella	LEFIJ142-10	MM09982	KM373601	BOLD:AAE9035	University of Oulu
Chionodes distinctella	NLLEA683-12	RMNH.INS.540878	KX050116	BOLD:AAE9034	Naturalis Biodiversity Centre
Chionodes distinctella	PHLAE083-11	TLMF Lep 04303	JN271070	BOLD:AAE9034	Tiroler Landesmuseum
Chionodes holosericella	LEALT227-16	TLMF Lep 20450	MG522176	BOLD:ABY6213	Tiroler Landesmuseum
Chionodes holosericella	LEALT044-16	TLMF Lep 20267	MG522520	BOLD:ABY6213	Tiroler Landesmuseum
Chionodes holosericella	LEALT043-16	TLMF Lep 20266	MG522244	BOLD:ABY6213	Tiroler Landesmuseum
Chionodes holosericella	LEALT042-16	TLMF Lep 20265	MG522282	BOLD:ABY6213	Tiroler Landesmuseum
Chionodes holosericella	PHLAB1114-10	TLMF Lep 01914	JF859626	BOLD:ABY6213	Tiroler Landesmuseum
Chionodes holosericella	LEATJ670-15	TLMF Lep 18245	MG522334	BOLD:ABY6213	Tiroler Landesmuseum
Chionodes holosericella	LEFIB739-10	MM02573	HM871617	BOLD:ABY6213	University of Oulu
Chionodes holosericella	LEATC433-13	TLMF Lep 12415	MG522114	BOLD:ABY6213	Tiroler Landesmuseum
Chionodes holosericella	PHLAB1116-10	TLMF Lep 01916	HQ968328	BOLD:ABY6213	Tiroler Landesmuseum
Chionodes holosericella	PHLAC681-10	TLMF Lep 02716	KX042596	BOLD:ABY6213	Tiroler Landesmuseum
Chionodes holosericella	LEATB003-13	TLMF Lep 10180	MG522448	BOLD:ABY6213	Tiroler Landesmuseum
Chionodes holosericella	PHLAD494-11	TLMF Lep 03669	JN271001	BOLD:ABY6213	Tiroler Landesmuseum
Chionodes holosericella	LEFIE025-10	MM08090	HM873774	BOLD:ABY6213	University of Oulu
Chionodes holosericella	PHLAB1113-10	TLMF Lep 01913	JF859625	BOLD:ABY6213	Tiroler Landesmuseum
Chionodes holosericella	LEATC432-13	TLMF Lep 12414	MG522842	BOLD:ABY6213	Naturmuseum Suedtirol
Chionodes holosericella	LEFIE969-10	MM10386	HM874686	BOLD:ABY6213	University of Oulu
Chionodes holosericella	LEATI277-15	TLMF Lep 17662	MG521993	BOLD:ABY6213	Tiroler Landesmuseum
Chionodes holosericella	PHLAB1115-10	TLMF Lep 01915	HQ968327	BOLD:ABY6213	Tiroler Landesmuseum
Chionodes holosericella	LEATB002-13	TLMF Lep 10179	MG522735	BOLD:ABY6213	Tiroler Landesmuseum

Chionodes holosericella	PHLAB1112-10	TLMF Lep 01912	JF859624	BOLD:ABY6213	Tiroler Landesmuseum
Coenonympha glycerion	LEALT290-16	TLMF Lep 20513	MG522386	BOLD:AAA9402	Tiroler Landesmuseum
Coenonympha glycerion	EZROM107-08	RV-07-D191	HQ004221	BOLD:AAA9402	Institut de Biologia Evolutiva (CSIC-UPF)
Coenonympha glycerion	PHLAH470-12	TLMF Lep 08289	KM573397	BOLD:AAA9402	inatura, Dornbirn
Coenonympha glycerion	LEFIE850-10	MM10081	HM874569	BOLD:AAA9402	University of Oulu
Coenonympha glycerion	EZROM106-08	RV-06-K649	HQ004222	BOLD:AAA9402	Institut de Biologia Evolutiva (CSIC-UPF)
Coenonympha glycerion	LEATF463-14	TLMF Lep 13775	MG522314	BOLD:AAA9402	Tiroler Landesmuseum
Coenonympha glycerion	LEFID313-10	MM06198	HM873110	BOLD:AAA9402	University of Oulu
Coenonympha glycerion	EZROM871-08	RVcoll.08-M381	HQ004223	BOLD:AAA9402	Institut de Biologia Evolutiva (CSIC-UPF)
Coenonympha glycerion	EZROM616-08	RV-07-D906	HQ004219	BOLD:AAA9402	Institut de Biologia Evolutiva (CSIC-UPF)
Coenonympha glycerion	FBLMV676-09	BC ZSM Lep 28656	GU707297	BOLD:AAA9402	SNSB, Zoologische Staatssammlung Muenchen
Coenonympha glycerion	FBLMU382-09	BC ZSM Lep 27032	GU707146	BOLD:AAA9402	SNSB, Zoologische Staatssammlung Muenchen
Coenonympha glycerion	LEALT386-16	TLMF Lep 20609	MG522192	BOLD:AAA9402	Tiroler Landesmuseum
Coenonympha glycerion	LEALT289-16	TLMF Lep 20512	MG522727	BOLD:AAA9402	Tiroler Landesmuseum
Coenonympha glycerion	EZROM870-08	RVcoll.08-M277	HQ004224	BOLD:AAA9402	Institut de Biologia Evolutiva (CSIC-UPF)
Coenonympha glycerion	EZROM867-08	RVcoll.08-M217	HQ004227	BOLD:AAA9402	Institut de Biologia Evolutiva (CSIC-UPF)
Coenonympha glycerion	GWORT467-10	BC ZSM Lep 32153	HM910012	BOLD:AAA9402	SNSB, Zoologische Staatssammlung Muenchen
Coenonympha glycerion	EZROM108-08	RV-07-C955	HQ004220	BOLD:AAA9402	Institut de Biologia Evolutiva (CSIC-UPF)
Coenonympha glycerion	EZROM869-08	RVcoll.08-M240	HQ004226	BOLD:AAA9402	Institut de Biologia Evolutiva (CSIC-UPF)
Coenonympha glycerion	EZROM868-08	RVcoll.08-M221	HQ004225	BOLD:AAA9402	Institut de Biologia Evolutiva (CSIC-UPF)
Coenonympha glycerion	PHLAB1222-10	TLMF Lep 02022	HQ968432	BOLD:AAA9402	Tiroler Landesmuseum
Coenonympha glycerion	PHLAA642-09	TLMF Lep 00682	HM426043	BOLD:AAA9402	Tiroler Landesmuseum
Coenonympha glycerion	PHLAA641-09	TLMF Lep 00681	HM426042	BOLD:AAA9402	Tiroler Landesmuseum
Coenonympha glycerion	LEFIG385-10	MM14398	HM876062	BOLD:AAA9402	University of Oulu
Coenonympha glycerion	GWORT464-10	BC ZSM Lep 32150	HM910010	BOLD:AAA9402	SNSB, Zoologische Staatssammlung Muenchen
Coenonympha glycerion	LEATF464-14	TLMF Lep 13776	MG522227	BOLD:AAA9402	Tiroler Landesmuseum
Coenonympha glycerion	FBLMU488-09	BC ZSM Lep 27138	HM391830	BOLD:AAA9402	SNSB, Zoologische Staatssammlung Muenchen
Coenonympha glycerion	GBLAA375-14	BC ZSM Lep 80371	MG522150	BOLD:AAA9402	SNSB, Zoologische Staatssammlung Muenchen
Coenonympha tullia	LOWA147-06	2005-LOWA-147	FJ663404	BOLD:AAA3563	McGuire Centre for Lepidoptera and Biodiversity
Coenonympha tullia	EZRMN361-08	RVcoll.08-M728	HQ004248	BOLD:AAA3563	Institut de Biologia Evolutiva (CSIC-UPF)

Coenonympha tullia	LEFIG583-10	MM14772	HM876254	BOLD:AAA3563	University of Oulu
Coenonympha tullia	LEALT529-16	TLMF Lep 20752	MG522064	BOLD:AAA3563	Tiroler Landesmuseum
Coenonympha tullia	LEATJ1252-16	TLMF Lep 19575	MG522528	BOLD:AAA3563	Tiroler Landesmuseum
Coenonympha tullia	LEATA404-13	TLMF Lep 10011	KM573064	BOLD:AAA3563	inatura, Dornbirn
Coenonympha tullia	LEATJ1253-16	TLMF Lep 19576	MG522743	BOLD:AAA3563	Tiroler Landesmuseum
Coenonympha tullia	EZROM114-08	RV-06-N023	HQ004251	BOLD:AAA3563	Institut de Biologia Evolutiva (CSIC-UPF)
Coenonympha tullia	LEFID529-10	MM06510	HM873294	BOLD:AAA3563	University of Oulu
Coenonympha tullia	GBLAB768-13	BC ZSM Lep 75539	MG522089	BOLD:AAA3563	SNSB, Zoologische Staatssammlung Muenchen
Coenonympha tullia	GWORT465-10	BC ZSM Lep 32151	HM910011	BOLD:AAA3563	SNSB, Zoologische Staatssammlung Muenchen
Coenonympha tullia	LEATI051-15	TLMF Lep 17436	MG522588	BOLD:AAA3563	Tiroler Landesmuseum
Coenonympha tullia	LEALT530-16	TLMF Lep 20753	MG522618	BOLD:AAA3563	Tiroler Landesmuseum
Coenonympha tullia	FBLMU482-09	BC ZSM Lep 27132	GU707176	BOLD:AAA3563	SNSB, Zoologische Staatssammlung Muenchen
Coenonympha tullia	LEATI050-15	TLMF Lep 17435	MG521958	BOLD:AAA3563	Tiroler Landesmuseum
Coenonympha tullia	LEFIC021-10	MM03232	HM871891	BOLD:AAA3563	University of Oulu
Coenonympha tullia	LOWA098-06	2005-LOWA-98	FJ663406	BOLD:AAA3563	McGuire Centre for Lepidoptera and Biodiversity
Coenonympha tullia	LEALT531-16	TLMF Lep 20754	MG522229	BOLD:AAA3563	Tiroler Landesmuseum
Coenonympha tullia	EZRMN359-08	RVcoll.08-M726	HQ004250	BOLD:AAA3563	Institut de Biologia Evolutiva (CSIC-UPF)
Coenonympha tullia	EZRMN362-08	RVcoll.08-M729	HQ004247	BOLD:AAA3563	Institut de Biologia Evolutiva (CSIC-UPF)
Coenonympha tullia	EZRMN360-08	RVcoll.08-M727	HQ004249	BOLD:AAA3563	Institut de Biologia Evolutiva (CSIC-UPF)
Colostygia aptata	LEATB822-13	TLMF Lep 10999	MG522125	BOLD:AAB9512	Tiroler Landesmuseum
Colostygia aptata	PHLAF362-11	TLMF Lep 05532	MG522422	BOLD:AAB9512	Tiroler Landesmuseum
Colostygia aptata	GWORK535-09	BC ZSM Lep 21865	JF415199	BOLD:AAB9512	SNSB, Zoologische Staatssammlung Muenchen
Colostygia aptata	LEFIC791-10	MM04930	HM872610	BOLD:AAB9512	University of Oulu
Colostygia aptata	PHLAF269-11	TLMF Lep 05439	MG522835	BOLD:AAB9512	Tiroler Landesmuseum
Colostygia aptata	ABOLB603-15	KLM Lep 02788	MG522018	BOLD:AAB9512	Landesmuseum Kaernten
Colostygia aptata	PHLSA746-11	TLMF Lep 06201	KM572561	BOLD:AAB9512	Tiroler Landesmuseum
Colostygia aptata	LEFIC792-10	MM04931	HM872611	BOLD:AAB9512	University of Oulu
Colostygia aptata	LEATD493-13	TLMF Lep 13140	MG522377	BOLD:AAB9512	Tiroler Landesmuseum
Colostygia aptata	PHLAH678-12	TLMF Lep 08497	KM573669	BOLD:AAB9512	inatura, Dornbirn
Colostygia aptata	LEFIA353-10	MM01400	HM386696	BOLD:AAB9512	University of Oulu

Colostygia aptata	PHLAF381-11	TLMF Lep 05551	MG522126	BOLD:AAB9512	Tiroler Landesmuseum
Colostygia aptata	GWORM118-09	BC ZSM Lep 24167	GU687233	BOLD:AAB9512	Research Collection of Alfred Haslberger
Colostygia aptata	LEATD565-13	TLMF Lep 13212	MG521914	BOLD:AAB9512	Tiroler Landesmuseum
Colostygia aptata	PHLAC451-10	TLMF Lep 02486	JF860026	BOLD:AAB9512	Tiroler Landesmuseum
Colostygia aptata	LEFIA324-10	MM01371	HM386667	BOLD:AAB9512	University of Oulu
Colostygia aptata	LEFIA323-10	MM01370	HM386666	BOLD:AAB9512	University of Oulu
Colostygia aptata	GWORP409-09	SE MNC Lep 00596	JN274057	BOLD:AAB9512	Museum fuer Naturkunde, Chemnitz
Colostygia aptata	GWOSA790-10	BC ZSM Lep 36656	HQ565497	BOLD:AAB9512	SNSB, Zoologische Staatssammlung Muenchen
Colostygia aptata	GWOR1688-07	BC ZSM Lep 01688	HQ601128	BOLD:AAB9512	SNSB, Zoologische Staatssammlung Muenchen
Colostygia aptata	LEALT474-16	TLMF Lep 20697	MG522251	BOLD:AAB9512	Tiroler Landesmuseum
Colostygia aptata	LEALT475-16	TLMF Lep 20698	MG522302	BOLD:AAB9512	Tiroler Landesmuseum
Coscinia cribraria	NLLEA524-12	RMNH.INS.540716	KX048935	BOLD:AAD9041	Naturalis Biodiversity Centre
Coscinia cribraria	PHLAD072-11	TLMF Lep 03057	JN307289	BOLD:ACE7017	Tiroler Landesmuseum
Coscinia cribraria	PHLAC495-10	TLMF Lep 02530	JF860067	BOLD:ABZ5211	Tiroler Landesmuseum
Coscinia cribraria	PHLAE182-11	TLMF Lep 04402	JN307404	BOLD:AAD9042	Tiroler Landesmuseum
Coscinia cribraria	PHLAD084-11	TLMF Lep 03069	JN307294	BOLD:AAD9042	Tiroler Landesmuseum
Coscinia cribraria	PHLAH755-12	TLMF Lep 08574	MG522678	BOLD:ABZ5211	Tiroler Landesmuseum
Coscinia cribraria	PHLAB1190-10	TLMF Lep 01990	HQ968400	BOLD:ACE7017	Tiroler Landesmuseum
Coscinia cribraria	PHLAG421-12	TLMF Lep 06815	MG522601	BOLD:AAD9041	Tiroler Landesmuseum
Coscinia cribraria	PHLAE171-11	TLMF Lep 04391	KX042130	BOLD:AAT9511	Tiroler Landesmuseum
Coscinia cribraria	PHLAE332-11	TLMF Lep 04647	JN307422	BOLD:ABZ5211	Tiroler Landesmuseum
Coscinia cribraria	PHLAD074-11	TLMF Lep 03059	JN307291	BOLD:ACE7017	Tiroler Landesmuseum
Coscinia cribraria	PHLAB1235-10	TLMF Lep 02035	HQ968443	BOLD:AAD9041	Tiroler Landesmuseum
Coscinia cribraria	PHLAH826-12	TLMF Lep 08630	MG522484	BOLD:ABZ5211	Tiroler Landesmuseum
Coscinia cribraria	NLLEA525-12	RMNH.INS.540717	KX050070	BOLD:AAD9041	Naturalis Biodiversity Centre
Coscinia cribraria	LEALT311-16	TLMF Lep 20534	MG522519	BOLD:AAD9041	Tiroler Landesmuseum
Coscinia cribraria	LEFIA1064-10	MM05671	KX041662	BOLD:AAD9041	University of Oulu
Coscinia cribraria	PHLAE181-11	TLMF Lep 04401	JN307403	BOLD:ABZ5211	Tiroler Landesmuseum
Coscinia cribraria	PHLAE172-11	TLMF Lep 04392	KX042015	BOLD:AAT9511	Tiroler Landesmuseum
Coscinia cribraria	LEFID778-10	MM06846	HM873535	BOLD:AAD9041	University of Oulu

Coscinia cribraria	PHLSA425-11	TLMF Lep 05880	MG522220	BOLD:ACE7017	Tiroler Landesmuseum
Coscinia cribraria	PHLAD679-11	TLMF Lep 04044	JN307361	BOLD:AAD9042	Tiroler Landesmuseum
Coscinia cribraria	LEFID630-10	MM06649	HM873393	BOLD:AAD9041	University of Oulu
Coscinia cribraria	PHLAD680-11	TLMF Lep 04045	JN307362	BOLD:AAD9042	Tiroler Landesmuseum
Coscinia cribraria	PHLAH568-12	TLMF Lep 08387	MG522261	BOLD:AAD9042	Tiroler Landesmuseum
Coscinia cribraria	PHLAD073-11	TLMF Lep 03058	JN307290	BOLD:ACE7017	Tiroler Landesmuseum
Coscinia cribraria	PHLAH570-12	TLMF Lep 08389	MG522577	BOLD:AAT9511	Tiroler Landesmuseum
Coscinia cribraria	NLLEA523-12	RMNH.INS.540715	KX049326	BOLD:AAD9041	Naturalis Biodiversity Centre
Coscinia cribraria	PHLSA424-11	TLMF Lep 05879	MG522748	BOLD:ACE7017	Tiroler Landesmuseum
Coscinia cribraria	PHLAH849-12	TLMF Lep 08653	MG522438	BOLD:AAT9511	Tiroler Landesmuseum
Coscinia cribraria	PHLSA600-11	TLMF Lep 06055	MG522620	BOLD:AAD9042	Tiroler Landesmuseum
Coscinia cribraria	PHLAH569-12	TLMF Lep 08388	MG522482	BOLD:AAD9042	Tiroler Landesmuseum
Coscinia cribraria	PHLAH825-12	TLMF Lep 08629	MG522164	BOLD:ABZ5211	Tiroler Landesmuseum
Coscinia cribraria	LEALT349-16	TLMF Lep 20572	MG522766	BOLD:AAD9041	Tiroler Landesmuseum
Coscinia cribraria	LEALT312-16	TLMF Lep 20535	MG522228	BOLD:AAD9041	Tiroler Landesmuseum
Coscinia cribraria	PHLAE183-11	TLMF Lep 04403	JN307405	BOLD:AAD9042	Tiroler Landesmuseum
Coscinia cribraria	PHLAD083-11	TLMF Lep 03068	JN307293	BOLD:AAD9042	Tiroler Landesmuseum
Coscinia cribraria	PHLAD085-11	TLMF Lep 03070	JN307295	BOLD:ABZ5211	Tiroler Landesmuseum
Coscinia cribraria	LEFIF551-10	MM12248	HM875236	BOLD:AAD9041	University of Oulu
Coscinia cribraria	PHLAA308-09	TLMF Lep 00348	HM425840	BOLD:AAD9042	Tiroler Landesmuseum
Coscinia cribraria	LEALT453-16	TLMF Lep 20676	MG522693	BOLD:AAD9041	Tiroler Landesmuseum
Coscinia cribraria	PHLAD762-11	TLMF Lep 04127	JN307372	BOLD:ABZ5211	Tiroler Landesmuseum
Coscinia cribraria	PHLAH850-12	TLMF Lep 08654	MG522358	BOLD:AAD9042	Tiroler Landesmuseum
Coscinia cribraria	PHLAB1189-10	TLMF Lep 01989	HQ968399	BOLD:ACE7017	Tiroler Landesmuseum
Coscinia cribraria	PHLAD761-11	TLMF Lep 04126	JN307371	BOLD:ABZ5211	Tiroler Landesmuseum
Crambus perlella	PHLAB1091-10	TLMF Lep 01891	HQ968317	BOLD:AAA6137	Tiroler Landesmuseum
Crambus perlella	LEATE855-13	TLMF Lep 12267	MG522613	BOLD:AAA6137	Tiroler Landesmuseum
Crambus perlella	LEALT114-16	TLMF Lep 20337	MG521969	BOLD:AAA6137	Tiroler Landesmuseum
Crambus perlella	LEFIB875-10	MM02946	HM871752	BOLD:AAA6137	University of Oulu
Crambus perlella	LEALT113-16	TLMF Lep 20336	MG522425	BOLD:AAA6137	Tiroler Landesmuseum

Crambus perlella	LEATE161-13	TLMF Lep 11573	MG522730	BOLD:AAA6137	Tiroler Landesmuseum
Crambus perlella	LEATB038-13	TLMF Lep 10215	MG522746	BOLD:AAA6137	Tiroler Landesmuseum
Crambus perlella	LEFIC806-10	MM04976	HM872625	BOLD:AAA6137	University of Oulu
Crambus perlella	LEFID559-10	MM06551	HM873324	BOLD:AAA6137	University of Oulu
Crambus perlella	LEALT120-16	TLMF Lep 20343	MG522043	BOLD:AAA6137	Tiroler Landesmuseum
Crambus perlella	LEATB944-13	TLMF Lep 11121	MG522715	BOLD:AAA6137	Tiroler Landesmuseum
Crambus perlella	PHLAV187-12	TLMF Lep 08006	KM573411	BOLD:AAA6137	inatura, Dornbirn
Crambus perlella	PHLAC073-10	TLMF Lep 02108	JF859695	BOLD:AAA6137	Tiroler Landesmuseum
Crambus perlella	LEFIB429-10	MM01915	HM871328	BOLD:AAA6137	University of Oulu
Crambus perlella	LEALT115-16	TLMF Lep 20338	MG521997	BOLD:AAA6137	Tiroler Landesmuseum
Crambus perlella	LEFIE100-10	MM08284	HM873848	BOLD:AAA6137	University of Oulu
Crambus perlella	PHLAB1090-10	TLMF Lep 01890	JF859619	BOLD:AAA6137	Tiroler Landesmuseum
Crocallis elinguaria	LEFIA321-10	MM01368	HM386664	BOLD:AAB0677	University of Oulu
Crocallis elinguaria	GBLAC707-13	BC ZSM Lep 77758	MG522632	BOLD:AAB0677	SNSB, Zoologische Staatssammlung Muenchen
Crocallis elinguaria	LEATB805-13	TLMF Lep 10982	MG522262	BOLD:AAB0677	Tiroler Landesmuseum
Crocallis elinguaria	GWORB3769-14	BC ZSM Lep 84611	MG522174	BOLD:AAB0677	SNSB, Zoologische Staatssammlung Muenchen
Crocallis elinguaria	GBLAA442-14	BC ZSM Lep 80533	MG522571	BOLD:AAB0677	SNSB, Zoologische Staatssammlung Muenchen
Crocallis elinguaria	GWOTL279-13	BC ZSM Lep 67305	MG522840	BOLD:AAB0677	SNSB, Zoologische Staatssammlung Muenchen
Crocallis elinguaria	NLLEA790-12	RMNH.INS.540985	KX049926	BOLD:AAB0677	Naturalis Biodiversity Centre
Crocallis elinguaria	GWOR081-07	BC ZSM Lep 00081	HQ601142	BOLD:AAB0677	SNSB, Zoologische Staatssammlung Muenchen
Crocallis elinguaria	LEATB806-13	TLMF Lep 10983	MG522285	BOLD:AAB0677	Tiroler Landesmuseum
Crocallis elinguaria	GWORE2205-09	BC ZSM Lep 24028	GU654886	BOLD:AAB0677	Research Collection of Alfred Haslberger
Crocallis elinguaria	GBLAF842-14	BC ZSM Lep 82928	MG522104	BOLD:AAE4231	SNSB, Zoologische Staatssammlung Muenchen
Crocallis elinguaria	LEATB714-13	TLMF Lep 10891	MG522741	BOLD:AAB0677	Naturmuseum Suedtirol
Crocallis elinguaria	GWOR4130-09	BC ZSM Lep 21414	HQ601144	BOLD:AAB0677	SNSB, Zoologische Staatssammlung Muenchen
Crocallis elinguaria	NLLEA818-12	RMNH.INS.541013	KX048303	BOLD:AAB0677	Naturalis Biodiversity Centre
Crocallis elinguaria	GWORG089-08	BC ZSM Lep 02909	JF415153	BOLD:AAB0677	SNSB, Zoologische Staatssammlung Muenchen
Crocallis elinguaria	LEALT558-16	TLMF Lep 20781	MG522182	BOLD:AAB0677	Tiroler Landesmuseum
Crocallis elinguaria	GBLAF840-14	BC ZSM Lep 82926	MG522175	BOLD:AAE4231	SNSB, Zoologische Staatssammlung Muenchen
Crocallis elinguaria	LEALT556-16	TLMF Lep 20779	MG521981	BOLD:AAB0677	Tiroler Landesmuseum

Crocallis elinguaria	ABOLA215-14	KLM Lep 01735	MG522496	BOLD:AAE4231	Landesmuseum Kaernten
Crocallis elinguaria	FBLMZ127-12	BC ZSM Lep 51383	KX041020	BOLD:AAB0677	SNSB, Zoologische Staatssammlung Muenchen
Crocallis elinguaria	LEFIA320-10	MM01367	HM386663	BOLD:AAB0677	University of Oulu
Crocallis elinguaria	LEALT557-16	TLMF Lep 20780	MG522406	BOLD:AAB0677	Tiroler Landesmuseum
Crocallis elinguaria	GBLAA1427-15	BC ZSM Lep 87218	MG522069	BOLD:AAB0677	SNSB, Zoologische Staatssammlung Muenchen
Crocallis elinguaria	PHLAD773-11	TLMF Lep 04138	JN269323	BOLD:AAB0677	Tiroler Landesmuseum
Crocallis elinguaria	GWORK525-09	BC ZSM Lep 21855	JF415154	BOLD:AAB0677	SNSB, Zoologische Staatssammlung Muenchen
Crocallis elinguaria	GBLAC870-13	BC ZSM Lep 77446	MG522095	BOLD:AAB0677	SNSB, Zoologische Staatssammlung Muenchen
Crocallis elinguaria	GBLAC656-13	BC ZSM Lep 78277	MG522030	BOLD:AAB0677	SNSB, Zoologische Staatssammlung Muenchen
Crocallis elinguaria	GWOR080-07	BC ZSM Lep 00080	HQ601143	BOLD:AAB0677	SNSB, Zoologische Staatssammlung Muenchen
Crocallis elinguaria	PHLSA668-11	TLMF Lep 06123	KM572315	BOLD:AAB0677	Tiroler Landesmuseum
Crocallis elinguaria	LEFIB839-10	MM02805	HM871716	BOLD:AAB0677	University of Oulu
Cupido minimus	GBLAA1364-15	BC ZSM Lep 87060	MG522365	BOLD:AAA9082	SNSB, Zoologische Staatssammlung Muenchen
Cupido minimus	LEFIF124-10	MM10580	HM874825	BOLD:AAA9082	University of Oulu
Cupido minimus	EZROM347-08	RV-08-A025	HQ004329	BOLD:AAA9082	Institut de Biologia Evolutiva (CSIC-UPF)
Cupido minimus	GWORL438-09	BC ZSM Lep 22340	GU686862	BOLD:AAA9082	SNSB, Zoologische Staatssammlung Muenchen
Cupido minimus	GBLAA1685-15	BC ZSM Lep 80066	MG521992	BOLD:AAA9082	SNSB, Zoologische Staatssammlung Muenchen
Cupido minimus	FBLMU452-09	BC ZSM Lep 27102	GU707085	BOLD:AAA9082	SNSB, Zoologische Staatssammlung Muenchen
Cupido minimus	EZROM135-08	RV-07-C941	HQ004337	BOLD:AAA9082	Institut de Biologia Evolutiva (CSIC-UPF)
Cupido minimus	GBLAA1686-15	BC ZSM Lep 80067	MG522351	BOLD:AAA9082	SNSB, Zoologische Staatssammlung Muenchen
Cupido minimus	GBLAD950-14	BC ZSM Lep 84556	MG522277	BOLD:AAA9082	SNSB, Zoologische Staatssammlung Muenchen
Cupido minimus	LEATG416-14	TLMF Lep 14203	MG522157	BOLD:AAA9082	Naturmuseum Suedtirol
Cupido minimus	GWORO792-09	BC ZSM Lep 30484	GU688441	BOLD:AAA9082	SNSB, Zoologische Staatssammlung Muenchen
Cupido minimus	LEFIF123-10	MM10579	HM874824	BOLD:AAA9082	University of Oulu
Cupido minimus	EZROM321-08	RV-07-E683	HQ004332	BOLD:AAA9082	Institut de Biologia Evolutiva (CSIC-UPF)
Cupido minimus	GBLAA1684-15	BC ZSM Lep 80065	MG522551	BOLD:AAA9082	SNSB, Zoologische Staatssammlung Muenchen
Cupido minimus	LEATG470-14	TLMF Lep 14257	MG522268	BOLD:AAA9082	Naturmuseum Suedtirol
Cupido minimus	PHLAH453-12	TLMF Lep 08272	KM573651	BOLD:AAA9082	inatura, Dornbirn
Cupido minimus	LEATF114-14	TLMF Lep 13426	MG522603	BOLD:AAA9082	Tiroler Landesmuseum
Cupido minimus	GBLAC745-13	BC ZSM Lep 77796	MG522253	BOLD:AAA9082	SNSB, Zoologische Staatssammlung Muenchen

Cupido minimus	LEATG417-14	TLMF Lep 14204	MG522456	BOLD:AAA9082	Naturmuseum Suedtirol
Cupido minimus	LEALT517-16	TLMF Lep 20740	MG522295	BOLD:AAA9082	Tiroler Landesmuseum
Cupido minimus	EZROM320-08	RV-07-E682	HQ004331	BOLD:AAA9082	Institut de Biologia Evolutiva (CSIC-UPF)
Cupido minimus	GBLAD265-14	BC ZSM Lep 78836	MG522029	BOLD:AAA9082	SNSB, Zoologische Staatssammlung Muenchen
Cupido minimus	LEATF113-14	TLMF Lep 13425	MG521937	BOLD:AAA9082	Tiroler Landesmuseum
Cupido minimus	EZROM898-08	RVcoll.08-M349	HQ004336	BOLD:AAA9082	Institut de Biologia Evolutiva (CSIC-UPF)
Cupido minimus	FBLMT889-09	BC ZSM Lep 25449	GU655019	BOLD:AAA9082	SNSB, Zoologische Staatssammlung Muenchen
Cupido minimus	LEFIL356-10	MM18666	JN276922	BOLD:AAA9082	University of Oulu
Cupido minimus	EZROM136-08	RV-07-E525	HQ004333	BOLD:AAA9082	Institut de Biologia Evolutiva (CSIC-UPF)
Cupido minimus	PHLAB299-10	TLMF Lep 01099	HQ968466	BOLD:AAA9082	Tiroler Landesmuseum
Cupido minimus	EZROM133-08	RV-06-N004	HQ004334	BOLD:AAA9082	Institut de Biologia Evolutiva (CSIC-UPF)
Cupido minimus	LEATH471-14	TLMF Lep 15683	MG522538	BOLD:AAA9082	Tiroler Landesmuseum
Cupido minimus	LEALT516-16	TLMF Lep 20739	MG521962	BOLD:AAA9082	Tiroler Landesmuseum
Cupido minimus	LEATD095-13	TLMF Lep 12742	MG522461	BOLD:AAA9082	Tiroler Landesmuseum
Cupido minimus	EZROM134-08	RV-07-D920	HQ004335	BOLD:AAA9082	Institut de Biologia Evolutiva (CSIC-UPF)
Cupido minimus	EZROM345-08	RV-08-A023	HQ004330	BOLD:AAA9082	Institut de Biologia Evolutiva (CSIC-UPF)
Cupido minimus	GBLAB120-13	BC ZSM Lep 75746	MG522178	BOLD:AAA9082	SNSB, Zoologische Staatssammlung Muenchen
Cupido minimus	EZROM622-08	RV-07-D521	HQ004328	BOLD:AAA9082	Institut de Biologia Evolutiva (CSIC-UPF)
Cupido minimus	GWORA2462-09	BC ZSM Lep 30674	HM393190	BOLD:AAA9082	Research Collection of Ralph Sturm
Cupido minimus magna	LOWA745-06	2005-LOWA-745	FJ663441	BOLD:AAA9082	McGuire Centre for Lepidoptera and Biodiversity
Cupido minimus magna	LOWA746-06	2005-LOWA-746	FJ663440	BOLD:AAA9082	McGuire Centre for Lepidoptera and Biodiversity
Cupido minimus magna	LOWA747-06	2005-LOWA-747	FJ663439	BOLD:AAA9082	McGuire Centre for Lepidoptera and Biodiversity
Cupido minimus minimus	LOWA186-06	2005-LOWA-186	FJ663442	BOLD:AAA9082	McGuire Centre for Lepidoptera and Biodiversity
Cupido minimus minimus	LOWA185-06	2005-LOWA-185	FJ663443	BOLD:AAA9082	McGuire Centre for Lepidoptera and Biodiversity
Cupido minimus minimus	LOWA184-06	2005-LOWA-184	FJ663444	BOLD:AAA9082	McGuire Centre for Lepidoptera and Biodiversity
Cyaniris semiargus	GWORL261-09	BC ZSM Lep 18667	GU686989	BOLD:AAB3027	Research Collection of Ralf Bolz
Cyaniris semiargus	EZROM904-08	RVcoll.08-M599	HQ004351	BOLD:AAB3027	Institut de Biologia Evolutiva (CSIC-UPF)
Cyaniris semiargus	EZROM568-08	RV-07-D530	HQ004346	BOLD:AAB3027	Institut de Biologia Evolutiva (CSIC-UPF)
Cyaniris semiargus	LEATC574-13	TLMF Lep 12556	MG522441	BOLD:AAB3027	Tiroler Landesmuseum
Cyaniris semiargus	LEALT377-16	TLMF Lep 20600	MG522634	BOLD:AAB3027	Tiroler Landesmuseum

Cyaniris semiargus	EZROM902-08	RVcoll.08-M251	HQ004350	BOLD:AAB3027	Institut de Biologia Evolutiva (CSIC-UPF)
Cyaniris semiargus	LEFIJ518-10	MM17143	JF853640	BOLD:AAB3027	University of Oulu
Cyaniris semiargus	EZROM903-08	RVcoll.08-M347	HQ004349	BOLD:AAB3027	Institut de Biologia Evolutiva (CSIC-UPF)
Cyaniris semiargus	LEATA409-13	TLMF Lep 10016	KM573008	BOLD:AAB3027	inatura, Dornbirn
Cyaniris semiargus	GBLAB128-13	BC ZSM Lep 75754	MG522529	BOLD:AAB3027	SNSB, Zoologische Staatssammlung Muenchen
Cyaniris semiargus	PHLSA438-11	TLMF Lep 05893	MG521946	BOLD:AAB3027	Tiroler Landesmuseum
Cyaniris semiargus	FBLMX195-11	BC ZSM Lep 50406	KP870306	BOLD:AAB3027	Research Collection of Alfred Haslberger
Cyaniris semiargus	EZROM138-08	RV-07-D334	HQ004348	BOLD:AAB3027	Institut de Biologia Evolutiva (CSIC-UPF)
Cyaniris semiargus	EZROM905-08	RVcoll.08-M643	HQ004352	BOLD:AAB3027	Institut de Biologia Evolutiva (CSIC-UPF)
Cyaniris semiargus	LOWA674-06	2005-LOWA-674	FJ663453	BOLD:AAB3027	McGuire Centre for Lepidoptera and Biodiversity
Cyaniris semiargus	LEATD458-13	TLMF Lep 13105	MG522291	BOLD:AAB3027	Tiroler Landesmuseum
Cyaniris semiargus	EZROM623-08	RV-07-C925	HQ004345	BOLD:AAB3027	Institut de Biologia Evolutiva (CSIC-UPF)
Cyaniris semiargus	LEATG225-14	TLMF Lep 14012	MG522383	BOLD:AAB3027	Tiroler Landesmuseum
Cyaniris semiargus	LEALT378-16	TLMF Lep 20601	MG522812	BOLD:AAB3027	Tiroler Landesmuseum
Cyaniris semiargus	EZROM139-08	RV-07-C985	HQ004347	BOLD:AAB3027	Institut de Biologia Evolutiva (CSIC-UPF)
Cyaniris semiargus	GWORL258-09	BC ZSM Lep 18664	GU686987	BOLD:AAB3027	Research Collection of Ralf Bolz
Cyaniris semiargus	GBLAB776-13	BC ZSM Lep 75547	MG522803	BOLD:AAB3027	SNSB, Zoologische Staatssammlung Muenchen
Cyaniris semiargus	FBLMW312-10	BC ZSM Lep 37413	HQ563552	BOLD:AAB3027	SNSB, Zoologische Staatssammlung Muenchen
Cyaniris semiargus	LEALT379-16	TLMF Lep 20602	MG522821	BOLD:AAB3027	Tiroler Landesmuseum
Cyaniris semiargus	LEATG104-14	TLMF Lep 13891	MG522124	BOLD:AAB3027	Tiroler Landesmuseum
Cyaniris semiargus	LEATG014-14	TLMF Lep 13801	MG522081	BOLD:AAB3027	Tiroler Landesmuseum
Cyaniris semiargus	LEFIJ520-10	MM17145	KM573316	BOLD:AAB3027	University of Oulu
Diarsia brunnea	LEATC243-13	TLMF Lep 11370	MG522359	BOLD:AAD6686	Naturmuseum Suedtirol
Diarsia brunnea	GBLAF293-14	BC ZSM Lep 81999	MG522575	BOLD:AAD6686	SNSB, Zoologische Staatssammlung Muenchen
Diarsia brunnea	LEFIA560-10	MM01654	KM573198	BOLD:AAD6686	University of Oulu
Diarsia brunnea	NLLEA346-12	RMNH.INS.538961	KX049766	BOLD:AAD6686	Naturalis Biodiversity Centre
Diarsia brunnea	GWORL291-09	BC ZSM Lep 22003	GU686969	BOLD:AAD6686	SNSB, Zoologische Staatssammlung Muenchen
Diarsia brunnea	LEFIB827-10	MM02773	HM871704	BOLD:AAD6686	University of Oulu
Diarsia brunnea	LEATB509-13	TLMF Lep 10686	MG522122	BOLD:AAD6686	Tiroler Landesmuseum
Diarsia brunnea	GBLAD745-14	BC ZSM Lep 84256	MG522789	BOLD:AAD6686	SNSB, Zoologische Staatssammlung Muenchen

Diarsia brunnea	GBLAD459-14	BC ZSM Lep 77985	MG522809	BOLD:AAD6686	SNSB, Zoologische Staatssammlung Muenchen
Diarsia brunnea	GBLAC544-13	BC ZSM Lep 78165	MG522472	BOLD:AAD6686	SNSB, Zoologische Staatssammlung Muenchen
Diarsia brunnea	LEALT545-16	TLMF Lep 20768	MG522680	BOLD:AAD6686	Tiroler Landesmuseum
Diarsia brunnea	LEALT544-16	TLMF Lep 20767	MG522676	BOLD:AAD6686	Tiroler Landesmuseum
Diarsia brunnea	ABOLA075-14	KLM Lep 01595	MG522460	BOLD:AAD6686	Landesmuseum Kaernten
Diarsia brunnea	LEFIK435-10	MM18010	KM573123	BOLD:AAD6686	University of Oulu
Diarsia brunnea	FBLMV025-09	BC ZSM Lep 28005	GU707437	BOLD:AAD6686	SNSB, Zoologische Staatssammlung Muenchen
Diarsia brunnea	ABOLC048-16	TLMF Lep 20081	MG521954	BOLD:AAD6686	Tiroler Landesmuseum
Diarsia brunnea	GBLAC559-13	BC ZSM Lep 78180	MG522287	BOLD:AAD6686	SNSB, Zoologische Staatssammlung Muenchen
Diarsia brunnea	LEATC205-13	TLMF Lep 11332	MG522384	BOLD:AAD6686	Tiroler Landesmuseum
Diarsia brunnea	PHLAC435-10	TLMF Lep 02470	JF860011	BOLD:AAD6686	Tiroler Landesmuseum
Diarsia brunnea	FBLMV026-09	BC ZSM Lep 28006	HQ955362	BOLD:AAD6686	SNSB, Zoologische Staatssammlung Muenchen
Diarsia brunnea	GBLAD132-14	BC ZSM Lep 78703	MG521973	BOLD:AAD6686	SNSB, Zoologische Staatssammlung Muenchen
Diarsia brunnea	PHLAV323-12	TLMF Lep 08142	KM572746	BOLD:AAD6686	inatura, Dornbirn
Diarsia brunnea	GBLAC494-13	BC ZSM Lep 78115	MG522570	BOLD:AAD6686	SNSB, Zoologische Staatssammlung Muenchen
Diarsia brunnea	GWOTL101-13	BC ZSM Lep 67032	MG522288	BOLD:AAD6686	SNSB, Zoologische Staatssammlung Muenchen
Diarsia brunnea	GWORO845-09	BC ZSM Lep 30537	GU688488	BOLD:AAD6686	SNSB, Zoologische Staatssammlung Muenchen
Diarsia brunnea	LEFIA559-10	MM01653	HQ963151	BOLD:AAD6686	University of Oulu
Diarsia brunnea	GBLAC1137-13	BC ZSM Lep 77428	MG522094	BOLD:AAD6686	SNSB, Zoologische Staatssammlung Muenchen
Diarsia brunnea	LEALT546-16	TLMF Lep 20769	MG522508	BOLD:AAD6686	Tiroler Landesmuseum
Diarsia brunnea	GWOTL261-13	BC ZSM Lep 67287	MG522040	BOLD:AAD6686	SNSB, Zoologische Staatssammlung Muenchen
Diarsia brunnea	GBLAA2023-15	BC ZSM Lep 100027	MG522177	BOLD:AAD6686	SNSB, Zoologische Staatssammlung Muenchen
Diarsia brunnea	GWOTL262-13	BC ZSM Lep 67288	MG522762	BOLD:AAD6686	SNSB, Zoologische Staatssammlung Muenchen
Diarsia mendica	LEALT485-16	TLMF Lep 20708	MG522387	BOLD:ABZ6600	Tiroler Landesmuseum
Diarsia mendica	LEALT486-16	TLMF Lep 20709	MG522434	BOLD:ABZ6600	Tiroler Landesmuseum
Diarsia mendica	LEALT478-16	TLMF Lep 20701	MG522617	BOLD:ABZ6600	Tiroler Landesmuseum
Diarsia mendica	GWOR4042-09	BC ZSM Lep 21326	JF415536	BOLD:ABZ6600	SNSB, Zoologische Staatssammlung Muenchen
Diarsia mendica	GBLAD764-14	BC ZSM Lep 84275	MG522218	BOLD:ABZ6600	SNSB, Zoologische Staatssammlung Muenchen
Diarsia mendica	LEATA394-13	TLMF Lep 10001	KP253323	BOLD:ABZ6600	inatura, Dornbirn

Diarsia mendica	LEFIA737-10	MM04091	HM386881	BOLD:ABZ6600	University of Oulu
Diarsia mendica	LEALT479-16	TLMF Lep 20702	MG521925	BOLD:ABZ6600	Tiroler Landesmuseum
Diarsia mendica	LEFIJ112-10	MM08075	KM573041	BOLD:AAB0038	University of Oulu
Diarsia mendica	LEFIA554-10	MM01647	KM572930	BOLD:AAB0038	University of Oulu
Diarsia mendica	LEFIA553-10	MM01646	KM573557	BOLD:AAB0038	University of Oulu
Diarsia mendica	ABOLA074-14	KLM Lep 01594	MG522044	BOLD:ABZ6600	Landesmuseum Kaernten
Diarsia mendica	LEFIJ046-10	MM02776	KM572703	BOLD:AAB0038	University of Oulu
Diarsia mendica	LEFIK340-10	MM17915	KM573297	BOLD:AAB0038	University of Oulu
Diarsia mendica	LEATB612-13	TLMF Lep 10789	MG522500	BOLD:ABZ6600	Naturmuseum Suedtirol
Diarsia mendica	NLLEA499-12	RMNH.INS.540691	KX048726	BOLD:ABZ6600	Naturalis Biodiversity Centre
Diarsia mendica	LEFIJ167-10	MM10960	KM573112	BOLD:ABZ6600	University of Oulu
Diarsia mendica	GWORO847-09	BC ZSM Lep 30539	GU688486	BOLD:ABZ6600	SNSB, Zoologische Staatssammlung Muenchen
Diarsia mendica	GWORL326-09	BC ZSM Lep 22038	GU686945	BOLD:ABZ6600	SNSB, Zoologische Staatssammlung Muenchen
Diarsia mendica	GWORL323-09	BC ZSM Lep 22035	GU686943	BOLD:ABZ6600	SNSB, Zoologische Staatssammlung Muenchen
Diarsia mendica	LEFIK434-10	MM18009	KM572634	BOLD:ABZ6600	University of Oulu
Diarsia mendica	GWORK413-09	BC ZSM Lep 21743	JF415535	BOLD:ABZ6600	SNSB, Zoologische Staatssammlung Muenchen
Diarsia mendica	LEATB425-13	TLMF Lep 10602	MG522827	BOLD:ABZ6600	Tiroler Landesmuseum
Diarsia mendica	PHLSA652-11	TLMF Lep 06107	KM573299	BOLD:ABZ6600	Tiroler Landesmuseum
Diarsia mendica	GBLAC711-13	BC ZSM Lep 77762	MG522135	BOLD:ABZ6600	SNSB, Zoologische Staatssammlung Muenchen
Diarsia mendica	LEFIJ047-10	MM02777	KM572872	BOLD:ABZ6600	University of Oulu
Diarsia mendica	LEFIJ139-10	MM09918	JF853453	BOLD:AAB0038	University of Oulu
Diarsia mendica	LEFIK433-10	MM18008	JF854058	BOLD:AAB0038	University of Oulu
Diarsia mendica	GWORR793-10	BC ZSM Lep 33999	HM904548		SNSB, Zoologische Staatssammlung Muenchen
Diarsia mendica	ABOLC110-16	TLMF Lep 20143	MG522083	BOLD:ABZ6600	Tiroler Landesmuseum
Diarsia mendica	LEFIJ120-10	MM08462	KM573068	BOLD:AAB0038	University of Oulu
Diarsia mendica	GWOR4043-09	BC ZSM Lep 21327	JF415534	BOLD:ABZ6600	SNSB, Zoologische Staatssammlung Muenchen
Diarsia mendica	LEFIJ1512-12	MM22780	KM572819	BOLD:AAB0038	University of Oulu
Diarsia mendica	LEFIJ068-10	MM05112	KM573116	BOLD:ABZ6600	University of Oulu
Diarsia mendica	LEATB801-13	TLMF Lep 10978	MG522606	BOLD:ABZ6600	Tiroler Landesmuseum
Diarsia mendica	PHLAC434-10	TLMF Lep 02469	JF860010	BOLD:ABZ6600	Tiroler Landesmuseum

Diarsia mendica	LEFIJ119-10	MM08461	KM572453	BOLD:AAB0038	University of Oulu
Dicallomera fascelina	LEATG191-14	TLMF Lep 13978	MG522458	BOLD:AAE7512	Tiroler Landesmuseum
Dicallomera fascelina	LEALT415-16	TLMF Lep 20638	MG522556	BOLD:AAT9838	Tiroler Landesmuseum
Dicallomera fascelina	LEATI380-15	TLMF Lep 17860	MG522186	BOLD:AAE7512	Tiroler Landesmuseum
Dicallomera fascelina	LEFID695-10	MM06746	HM873456	BOLD:AAE7512	University of Oulu
Dicallomera fascelina	PHLSA648-11	TLMF Lep 06103	KM573275	BOLD:AAE7512	Tiroler Landesmuseum
Dicallomera fascelina	LEATG210-14	TLMF Lep 13997	MG522345	BOLD:AAE7512	Tiroler Landesmuseum
Dicallomera fascelina	PHLAH818-12	TLMF Lep 08622	MG522837	BOLD:AAE7512	Tiroler Landesmuseum
Dicallomera fascelina	LEFIG984-10	MM15848	HM876623	BOLD:AAE7512	University of Oulu
Dicallomera fascelina	PHLAA352-09	TLMF Lep 00392	HM425878	BOLD:AAE7512	Tiroler Landesmuseum
Dicallomera fascelina	LEALT414-16	TLMF Lep 20637	MG521922	BOLD:AAT9838	Tiroler Landesmuseum
Dicallomera fascelina	LEALT303-16	TLMF Lep 20526	MG522313	BOLD:AAT9838	Tiroler Landesmuseum
Dicallomera fascelina	ABOLB764-15	KLM Lep 03424	MG522378	BOLD:AAE7512	Landesmuseum Kaernten
Dicallomera fascelina	LEFIE250-10	MM08578	HM873984	BOLD:AAE7512	University of Oulu
Eana osseana	PHLAC287-10	TLMF Lep 02322	JF859890	BOLD:AAA6265	Tiroler Landesmuseum
Eana osseana	LEFIE951-10	MM10348	HM874668	BOLD:AAA6265	University of Oulu
Eana osseana	LEALT009-16	TLMF Lep 20232	MG522771	BOLD:AAA4129	Tiroler Landesmuseum
Eana osseana	LEATE202-13	TLMF Lep 11614	MG522328	BOLD:AAA6265	Tiroler Landesmuseum
Eana osseana	LEALT011-16	TLMF Lep 20234	MG522591	BOLD:AAA4129	Tiroler Landesmuseum
Eana osseana	LEFIB897-10	MM02993	HM871774	BOLD:AAA6265	University of Oulu
Eana osseana	LEALT010-16	TLMF Lep 20233	MG522506	BOLD:AAA4129	Tiroler Landesmuseum
Eana osseana	LEATE203-13	TLMF Lep 11615	MG521940	BOLD:AAA6265	Tiroler Landesmuseum
Eana osseana	PHLAB914-10	TLMF Lep 01714	HQ968918	BOLD:AAA6265	Tiroler Landesmuseum
Eana osseana	PHLAC286-10	TLMF Lep 02321	JF859889	BOLD:AAA6265	Tiroler Landesmuseum
Eana osseana	LEFIA786-10	MM04140	HM386927	BOLD:AAA6265	University of Oulu
Eana osseana	PHLAI590-13	TLMF Lep 09152	KM571988	BOLD:AAA6265	Tiroler Landesmuseum
Eana penziana	PHLAB140-10	TLMF Lep 00940	HM381508	BOLD:AAB8228	Tiroler Landesmuseum
Eana penziana	PHLAA719-09	TLMF Lep 00759	HM426113	BOLD:AAB8228	Tiroler Landesmuseum
Eana penziana	PHLAF094-11	TLMF Lep 05264	MG522602	BOLD:AAB8228	Tiroler Landesmuseum
Eana penziana	PHLAA540-09	TLMF Lep 00580	HM381422	BOLD:AAB8228	Tiroler Landesmuseum

Eana penziana	LEFIC119-10	MM03455	HM871966	BOLD:AAB8228	University of Oulu
Eana penziana	PHLAB261-10	TLMF Lep 01061	HM381621	BOLD:AAB8228	Tiroler Landesmuseum
Eana penziana	LEALT097-16	TLMF Lep 20320	MG522050	BOLD:AAB8228	Tiroler Landesmuseum
Eana penziana	LEFIC154-10	MM03500	HM872000	BOLD:AAB8228	University of Oulu
Eana penziana	LEFIE117-10	MM08314	HM873865	BOLD:AAB8228	University of Oulu
Eana penziana	LEALT098-16	TLMF Lep 20321	MG522754	BOLD:AAB8228	Tiroler Landesmuseum
Eana penziana	LEATA380-13	TLMF Lep 09797	MG522640	BOLD:AAB8228	Tiroler Landesmuseum
Eana penziana	PHLAD430-11	TLMF Lep 03605	JN286453	BOLD:AAB8228	Tiroler Landesmuseum
Eana penziana	PHLAA079-09	TLMF Lep 00119	GU689178	BOLD:AAB8228	Tiroler Landesmuseum
Eana penziana	LEATB218-13	TLMF Lep 10395	MG521974	BOLD:AAB8228	Tiroler Landesmuseum
Eana penziana	PHLAB910-10	TLMF Lep 01710	HQ968914	BOLD:AAB8228	Tiroler Landesmuseum
Eana penziana	PHLAB908-10	TLMF Lep 01708	HQ968912	BOLD:AAB8228	Tiroler Landesmuseum
Eana penziana	LEFIE108-10	MM08293	HM873856	BOLD:AAB8228	University of Oulu
Eana penziana	PHLAB192-10	TLMF Lep 00992	HM381556	BOLD:AAB8228	Tiroler Landesmuseum
Eana penziana	LEATB012-13	TLMF Lep 10189	MG522201	BOLD:AAB8228	Tiroler Landesmuseum
Eana penziana	PHLAA080-09	TLMF Lep 00120	GU689179	BOLD:AAB8228	Tiroler Landesmuseum
Eana penziana	LEALT096-16	TLMF Lep 20319	MG522504	BOLD:AAB8228	Tiroler Landesmuseum
Eana penziana	PHLAB783-10	TLMF Lep 01583	HQ968791	BOLD:AAB8228	Tiroler Landesmuseum
Eana penziana	LEATF271-14	TLMF Lep 13583	MG522273	BOLD:AAB8228	Tiroler Landesmuseum
Eana penziana	FBLMZ149-12	BC ZSM Lep 51405	KX041177	BOLD:AAB8228	SNSB, Zoologische Staatssammlung Muenchen
Eana penziana	PHLAB125-10	TLMF Lep 00925	HM381495	BOLD:AAB8228	Tiroler Landesmuseum
Eana penziana	PHLAB126-10	TLMF Lep 00926	HM381496	BOLD:AAB8228	Tiroler Landesmuseum
Eana penziana	PHLAB909-10	TLMF Lep 01709	HQ968913	BOLD:AAB8228	Tiroler Landesmuseum
Eana penziana	PHLAB124-10	TLMF Lep 00924	HM381494	BOLD:AAB8228	Tiroler Landesmuseum
Eana penziana	PHLAI055-12	KLM Lep 00530	MG522235	BOLD:AAB8228	Landesmuseum Kaernten
Eana penziana	PHLAB784-10	TLMF Lep 01584	HQ968792	BOLD:AAB8228	Tiroler Landesmuseum
Eilema lutarella	LEALT439-16	TLMF Lep 20662	MG522554	BOLD:ABZ2872	Tiroler Landesmuseum
Eilema lutarella	LEALT440-16	TLMF Lep 20663	MG522214	BOLD:ABZ2872	Tiroler Landesmuseum
Eilema lutarella	LEFIC753-10	MM04868	HM872572	BOLD:ABZ2872	University of Oulu
Eilema lutarella	ABOLB752-15	KLM Lep 03412	MG522051	BOLD:ABZ2872	Landesmuseum Kaernten

Eilema lutarella	LEFIJ825-10	MM17450	KT782737	BOLD:ABZ2872	University of Oulu
Eilema lutarella	FBLMU538-09	BC ZSM Lep 27188	HM391855	BOLD:ABZ2872	SNSB, Zoologische Staatssammlung Muenchen
Eilema lutarella	LEATG206-14	TLMF Lep 13993	MG521917	BOLD:ABZ2872	Tiroler Landesmuseum
Eilema lutarella	FBLMU540-09	BC ZSM Lep 27190	HM391856	BOLD:ABZ2872	SNSB, Zoologische Staatssammlung Muenchen
Eilema lutarella	LEFIA097-10	MM01020	HM396444	BOLD:ABZ2872	University of Oulu
Eilema lutarella	GWORO777-09	BC ZSM Lep 30469	GU688497	BOLD:ABZ2872	SNSB, Zoologische Staatssammlung Muenchen
Eilema lutarella	ABOLA134-14	KLM Lep 01654	MG522557	BOLD:ABZ2872	Landesmuseum Kaernten
Eilema lutarella	LEFIJ575-10	MM17200	JF853688	BOLD:ABZ2872	University of Oulu
Eilema lutarella	GBLAF393-14	BC ZSM Lep 82099	MG522511	BOLD:ABZ2872	SNSB, Zoologische Staatssammlung Muenchen
Eilema lutarella	GBLAA397-14	BC ZSM Lep 80488	MG522651	BOLD:ABZ2872	SNSB, Zoologische Staatssammlung Muenchen
Eilema lutarella	ABOLB753-15	KLM Lep 03413	MG522353	BOLD:ABZ2872	Landesmuseum Kaernten
Eilema lutarella	LEFIA098-10	MM01021	HM396445	BOLD:ABZ2872	University of Oulu
Eilema lutarella	PHLAA402-09	TLMF Lep 00442	HM425920	BOLD:ABZ2872	Tiroler Landesmuseum
Elachista bedellella	LEATJ561-15	TLMF Lep 18136	MG522559	BOLD:AAQ0947	Tiroler Landesmuseum
Elachista bedellella	LEATC394-13	TLMF Lep 12376	MG522774	BOLD:AAQ0947	Naturmuseum Suedtirol
Elachista bedellella	LEATJ402-15	TLMF Lep 17977	MG522102	BOLD:AAQ0947	Tiroler Landesmuseum
Elachista bedellella	GBLAB1277-14	BC ZSM Lep 76427	MG522567	BOLD:AAQ0947	SNSB, Zoologische Staatssammlung Muenchen
Elachista bedellella	GBLAB1270-14	BC ZSM Lep 76420	MG522696	BOLD:AAQ0947	SNSB, Zoologische Staatssammlung Muenchen
Elachista bedellella	LASTS707-15	TLMF Lep 16572	MG522560	BOLD:AAQ0947	Tiroler Landesmuseum
Elachista bedellella	LEATJ563-15	TLMF Lep 18138	MG522637	BOLD:AAQ0947	Tiroler Landesmuseum
Elachista bedellella	LASTS706-15	TLMF Lep 16571	MG522542	BOLD:AAQ0947	Tiroler Landesmuseum
Elachista bedellella	LASTS733-15	TLMF Lep 16598	MG522217	BOLD:AAQ0947	Tiroler Landesmuseum
Elachista bedellella	PHLAI139-12	KLM Lep 00614	MG522331	BOLD:AAQ0947	Landesmuseum Kaernten
Elachista bedellella	LEALT221-16	TLMF Lep 20444	MG522503	BOLD:ACF1904	Tiroler Landesmuseum
Elachista bedellella	LEALT222-16	TLMF Lep 20445	MG522622	BOLD:ACF1904	Tiroler Landesmuseum
Elachista bedellella	LEALT220-16	TLMF Lep 20443	MG522088	BOLD:ACF1904	Tiroler Landesmuseum
Entephria caesiata	LEALT469-16	TLMF Lep 20692	MG522467	BOLD:AAB9028	Tiroler Landesmuseum
Entephria caesiata	LEALT468-16	TLMF Lep 20691	MG522734	BOLD:AAB9028	Tiroler Landesmuseum
Entephria caesiata	LEALT467-16	TLMF Lep 20690	MG522131	BOLD:AAB9028	Tiroler Landesmuseum
Entephria caesiata	GBLAD934-14	BC ZSM Lep 84540	MG522666	BOLD:AAB9028	SNSB, Zoologische Staatssammlung Muenchen

Entephria caesiata	GBLAC415-13	BC ZSM Lep 77656	MG522021	BOLD:AAB9028	SNSB, Zoologische Staatssammlung Muenchen
Entephria caesiata	LEATB510-13	TLMF Lep 10687	MG522614	BOLD:AAB9028	Tiroler Landesmuseum
Entephria caesiata	ABOLB834-15	KLM Lep 03494	MG522543	BOLD:AAB9028	Landesmuseum Kaernten
Entephria caesiata	PHLAG882-12	TLMF Lep 07561	KM572419	BOLD:AAB9028	inatura, Dornbirn
Entephria caesiata	PHLAC418-10	TLMF Lep 02453	JF859994	BOLD:AAB9028	Tiroler Landesmuseum
Entephria caesiata	GWOR1671-07	BC ZSM Lep 01671	HQ601202	BOLD:AAB9028	SNSB, Zoologische Staatssammlung Muenchen
Entephria caesiata	GWORK538-09	BC ZSM Lep 21868	JF415214	BOLD:AAB9028	SNSB, Zoologische Staatssammlung Muenchen
Entephria caesiata	GMGRD3598-13	BIOUG06173-B06	MG522828	BOLD:AAB9028	SNSB, Zoologische Staatssammlung Muenchen
Entephria caesiata	GWORM079-09	BC ZSM Lep 24128	GU687267	BOLD:AAB9028	Research Collection of Alfred Haslberger
Entephria caesiata	GBLAC391-13	BC ZSM Lep 77632	MG522407	BOLD:AAB9028	SNSB, Zoologische Staatssammlung Muenchen
Entephria caesiata	LEATD570-13	TLMF Lep 13217	MG522491	BOLD:AAB9028	Tiroler Landesmuseum
Entephria caesiata	PHLAA221-09	TLMF Lep 00261	HM425768	BOLD:AAB9028	Tiroler Landesmuseum
Entephria caesiata	GWORM078-09	BC ZSM Lep 24127	GU687266	BOLD:AAB9028	Research Collection of Alfred Haslberger
Entephria caesiata	GWORK536-09	BC ZSM Lep 21866	JF415215	BOLD:AAB9028	SNSB, Zoologische Staatssammlung Muenchen
Entephria caesiata	NOLEP062-14	BCZMBNLep0062	MG521979	BOLD:AAB9028	University of Bergen, Natural History Collections
Entephria caesiata	GWORL344-09	BC ZSM Lep 22056	GU686924	BOLD:AAB9028	SNSB, Zoologische Staatssammlung Muenchen
Entephria caesiata	LEFIB292-10	MM00796	HM871193	BOLD:AAB9028	University of Oulu
Entephria caesiata	PHLAA331-09	TLMF Lep 00371	HQ968173	BOLD:AAB9028	Tiroler Landesmuseum
Entephria caesiata	GBLAC931-13	BC ZSM Lep 77507	MG522833	BOLD:AAB9028	SNSB, Zoologische Staatssammlung Muenchen
Entephria caesiata	LEATB552-13	TLMF Lep 10729	MG522329	BOLD:AAB9028	Naturmuseum Suedtirol
Entephria caesiata	NOLEP053-14	BCZMBNLep0053	MG522143	BOLD:AAB9028	University of Bergen, Natural History Collections
Entephria caesiata	GWORA2062-09	BC ZSM Lep 26464	GU655467	BOLD:AAB9028	SNSB, Zoologische Staatssammlung Muenchen
Entephria caesiata	LEFIE033-10	MM08109	HM873782	BOLD:AAB9028	University of Oulu
Entephria caesiata	GBLAD935-14	BC ZSM Lep 84541	MG522270	BOLD:AAB9028	SNSB, Zoologische Staatssammlung Muenchen
Entephria caesiata	LEFIB857-10	MM02878	HM871734	BOLD:AAB9028	University of Oulu
Entephria caesiata	GBLAC927-13	BC ZSM Lep 77503	MG522646	BOLD:AAB9028	SNSB, Zoologische Staatssammlung Muenchen
Epermenia illigerella	LEALT041-16	TLMF Lep 20264	MG522550	BOLD:AAE3914	Tiroler Landesmuseum
Epermenia illigerella	LEATE607-13	TLMF Lep 12019	MG522338	BOLD:AAE3914	Tiroler Landesmuseum
Epermenia illigerella	FBLMZ620-12	BC ZSM Lep 61471	KX045277	BOLD:AAE3914	Research Collection of Peter Lichtmannecker
Epermenia illigerella	LEFIE423-10	MM09033	HM874147	BOLD:AAE3914	University of Oulu

Epermenia illigerella	PHLAB074-10	TLMF Lep 00874	HQ968248	BOLD:AAE3914	Tiroler Landesmuseum
Epermenia illigerella	PHLAH159-12	TLMF Lep 06659	MG522786	BOLD:AAE3914	Research Collection of Peter Buchner
Epermenia illigerella	LEFIC900-10	MM05213	HM872717	BOLD:AAE3914	University of Oulu
Epermenia illigerella	LEALT040-16	TLMF Lep 20263	MG521928	BOLD:AAE3914	Tiroler Landesmuseum
Epermenia illigerella	LEFIE424-10	MM09035	HM874148	BOLD:AAE3914	University of Oulu
Epermenia illigerella	DEEUR491-15	TLMF Lep 17686	MG522154	BOLD:AAE3914	Research Collection of Peter Buchner
Epermenia illigerella	LEALT039-16	TLMF Lep 20262	MG522524	BOLD:AAE3914	Tiroler Landesmuseum
Epermenia illigerella	PHLAI675-13	TLMF Lep 09237	MG522758	BOLD:AAE3914	Tiroler Landesmuseum
Epermenia illigerella	PHLAG600-12	TLMF Lep 07279	MG522278	BOLD:AAE3914	Tiroler Landesmuseum
Epermenia illigerella	LEFIC142-10	MM03487	HM871988	BOLD:AAE3914	University of Oulu
Epermenia illigerella	LEFIB715-10	MM02527	HM871593	BOLD:AAE3914	University of Oulu
Epermenia illigerella	FBLMZ625-12	BC ZSM Lep 61476	KX046489	BOLD:AAE3914	Research Collection of Peter Lichtmannecker
Epinotia cruciana	LEFIE133-10	MM08349	HM873881	BOLD:AAC2644	University of Oulu
Epinotia cruciana	LEFID555-10	MM06544	HM873320	BOLD:AAC2644	University of Oulu
Epinotia cruciana	LEATB942-13	TLMF Lep 11119	MG522155	BOLD:AAC2644	Tiroler Landesmuseum
Epinotia cruciana	FBLMZ717-12	BC ZSM Lep 61568	KX044327	BOLD:AAC2644	Research Collection of Peter Lichtmannecker
Epinotia cruciana	LEALT021-16	TLMF Lep 20244	MG522830	BOLD:AAC2644	Tiroler Landesmuseum
Epinotia cruciana	LEALT019-16	TLMF Lep 20242	MG522795	BOLD:AAC2644	Tiroler Landesmuseum
Epinotia cruciana	LEATE577-13	TLMF Lep 11989	MG522705	BOLD:ACE9994	Tiroler Landesmuseum
Epinotia cruciana	LEATE576-13	TLMF Lep 11988	MG522533	BOLD:ACE9994	Naturmuseum Suedtirol
Epinotia cruciana	LEALT020-16	TLMF Lep 20243	MG522341	BOLD:AAC2644	Tiroler Landesmuseum
Epinotia cruciana	LEFID821-10	MM06921	HM873578	BOLD:AAC2644	University of Oulu
Epinotia cruciana	PHLAA758-09	TLMF Lep 00798	HM426142	BOLD:AAC2644	Tiroler Landesmuseum
Epinotia cruciana	LEFIC053-10	MM03323	HM871922	BOLD:AAC2644	University of Oulu
Epinotia cruciana	LEATB928-13	TLMF Lep 11105	MG522180	BOLD:AAC2644	Tiroler Landesmuseum
Epinotia cruciana	FBLMZ232-12	BC ZSM Lep 64313	KX046895	BOLD:AAC2644	Research Collection of Alfred Haslberger
Epinotia trigonella	LEFIF573-10	MM12351	HM875258	BOLD:AAB2504	University of Oulu
Epinotia trigonella	LEFIC679-10	MM04647	HM872500	BOLD:AAB2504	University of Oulu
Epinotia trigonella	PHLAH374-12	TLMF Lep 07813	MG522163	BOLD:AAB2504	Tiroler Landesmuseum
Epinotia trigonella	PHLAH940-12	TLMF Lep 08744	KM572657	BOLD:AAB2504	Tiroler Landesmuseum

Epinotia trigonella	LEFIK746-10	MM18321	JF854306	BOLD:AAB2504	University of Oulu
Epinotia trigonella	LEFIB481-10	MM02047	HQ570263	BOLD:AAB2504	University of Oulu
Epinotia trigonella	LEALT005-16	TLMF Lep 20228	MG522723	BOLD:AAB2504	Tiroler Landesmuseum
Epinotia trigonella	ABOLB375-15	TLMF Lep 17380	MG522473	BOLD:AAB2504	Tiroler Landesmuseum
Epinotia trigonella	LEALT006-16	TLMF Lep 20229	MG522593	BOLD:AAB2504	Tiroler Landesmuseum
Epinotia trigonella	ABOLA361-14	TLMF Lep 15383	MG522368	BOLD:AAB2504	Tiroler Landesmuseum
Epinotia trigonella	LEFIF861-10	MM13231	HM875543	BOLD:AAB2504	University of Oulu
Epinotia trigonella	LEALT004-16	TLMF Lep 20227	MG522076	BOLD:AAB2504	Tiroler Landesmuseum
Eudonia alpina	LEFID438-10	MM06370	HQ570334	BOLD:AAB0095	University of Oulu
Eudonia alpina	LEFIB192-10	MM00623	HM871096	BOLD:AAB0095	University of Oulu
Eudonia alpina	LEALT068-16	TLMF Lep 20291	MG522699	BOLD:AAB0095	Tiroler Landesmuseum
Eudonia alpina	LEFIB191-10	MM00622	HM871095	BOLD:AAB0095	University of Oulu
Eudonia alpina	LEALT069-16	TLMF Lep 20292	MG522684	BOLD:AAB0095	Tiroler Landesmuseum
Eulamprotes wilkella	LEALT103-16	TLMF Lep 20326	MG522523	BOLD:AAF7554	Tiroler Landesmuseum
Eulamprotes wilkella	PHLAD012-11	TLMF Lep 02997	JN262187	BOLD:AAF7554	Tiroler Landesmuseum
Eulamprotes wilkella	NLLEA1353-14	RMNH.INS.552329	MG522048	BOLD:AAF7554	Naturalis Biodiversity Centre
Eulamprotes wilkella	LEATJ1446-16	TLMF Lep 19769	MG521923	BOLD:AAF7554	Tiroler Landesmuseum
Eulamprotes wilkella	PHLAF126-11	TLMF Lep 05296	KF714889	BOLD:AAF7554	Tiroler Landesmuseum
Eulamprotes wilkella	PHLAJ035-13	KLM Lep 00795	KF714880	BOLD:AAF7554	Landesmuseum Kaernten
Eulamprotes wilkella	PHLAJ029-13	KLM Lep 00789	KF714892	BOLD:ACF6810	Landesmuseum Kaernten
Eulamprotes wilkella	LEFIG081-10	MM13733	HM875760	BOLD:AAF7554	University of Oulu
Eulamprotes wilkella	LEATF023-14	TLMF Lep 13335	MG522415	BOLD:AAF7554	Tiroler Landesmuseum
Eulamprotes wilkella	PHLAB828-10	TLMF Lep 01628	HQ968832	BOLD:AAF7554	Tiroler Landesmuseum
Eulamprotes wilkella	LEFIF499-10	MM12068	HM875184	BOLD:AAF7554	University of Oulu
Eulamprotes wilkella	ABOLB181-15	TLMF Lep 17186	MG522275	BOLD:AAF7554	Tiroler Landesmuseum
Eulamprotes wilkella	LEATC473-13	TLMF Lep 12455	MG522814	BOLD:AAF7554	Tiroler Landesmuseum
Eulamprotes wilkella	LEALT102-16	TLMF Lep 20325	MG522356	BOLD:AAF7554	Tiroler Landesmuseum
Eulamprotes wilkella	LEATJ1441-16	TLMF Lep 19764	MG522309	BOLD:AAF7554	Tiroler Landesmuseum
Eulamprotes wilkella	LEFIG474-10	MM14521	HM876150	BOLD:AAF7554	University of Oulu
Eulamprotes wilkella	NLLEA1383-14	RMNH.INS.558000	MG522781	BOLD:AAF7554	Naturalis Biodiversity Centre

Eulamprotes wilkella	LEALT104-16	TLMF Lep 20327	MG522119	BOLD:AAF7554	Tiroler Landesmuseum
Eulamprotes wilkella	LEATE454-13	TLMF Lep 11866	MG522581	BOLD:ACF6810	Tiroler Landesmuseum
Eulithis populata	GBLAC860-13	BC ZSM Lep 77436	MG522648	BOLD:ABZ1837	SNSB, Zoologische Staatssammlung Muenchen
Eulithis populata	GWORO975-09	BC ZSM Lep 32282	GU688555	BOLD:ABZ1837	SNSB, Zoologische Staatssammlung Muenchen
Eulithis populata	GWOR3784-09	BC ZSM Lep 21068	HQ601244	BOLD:ABZ1837	SNSB, Zoologische Staatssammlung Muenchen
Eulithis populata	GWORO958-09	BC ZSM Lep 32265	GU688570	BOLD:ABZ1837	SNSB, Zoologische Staatssammlung Muenchen
Eulithis populata	PHLSA710-11	TLMF Lep 06165	KM573042	BOLD:ABZ1837	Tiroler Landesmuseum
Eulithis populata	GBLGC118-12	BC ZSM Lep R 21068	MG522813	BOLD:ABZ1837	SNSB, Zoologische Staatssammlung Muenchen
Eulithis populata	GWORB840-07	BC ZSM Lep 01216	HQ601243	BOLD:ABZ1837	SNSB, Zoologische Staatssammlung Muenchen
Eulithis populata	GBLAB103-13	BC ZSM Lep 75729	MG522023	BOLD:ABZ1837	SNSB, Zoologische Staatssammlung Muenchen
Eulithis populata	LEALT402-16	TLMF Lep 20625	MG522638	BOLD:ABZ1837	Tiroler Landesmuseum
Eulithis populata	LEATB828-13	TLMF Lep 11005	MG522202	BOLD:ABZ1837	Tiroler Landesmuseum
Eulithis populata	PHLSA711-11	TLMF Lep 06166	KM572127	BOLD:ABZ1837	Tiroler Landesmuseum
Eulithis populata	LEATB288-13	TLMF Lep 10465	MG522653	BOLD:ABZ1837	Naturmuseum Suedtirol
Eulithis populata	LEFIE024-10	MM08084	HM873773	BOLD:ABZ1837	University of Oulu
Eulithis populata	GBLAC632-13	BC ZSM Lep 78253	MG521959	BOLD:ABZ1837	SNSB, Zoologische Staatssammlung Muenchen
Eulithis populata	GBLAC876-13	BC ZSM Lep 77452	MG522619	BOLD:ABZ1837	SNSB, Zoologische Staatssammlung Muenchen
Eulithis populata	GBLAA2337-15	BC ZSM Lep 90028	MG522079	BOLD:ABZ1837	SNSB, Zoologische Staatssammlung Muenchen
Eulithis populata	LEATC146-13	TLMF Lep 11273	MG522768	BOLD:ABZ1837	Tiroler Landesmuseum
Eulithis populata	GWORK524-09	BC ZSM Lep 21854	JF415229	BOLD:ABZ1837	SNSB, Zoologische Staatssammlung Muenchen
Eulithis populata	LEALT400-16	TLMF Lep 20623	MG521971	BOLD:ABZ1837	Tiroler Landesmuseum
Eulithis populata	LEFIC885-10	MM05173	HM872703	BOLD:ABZ1837	University of Oulu
Eulithis populata	GWORL346-09	BC ZSM Lep 22058	GU686925	BOLD:ABZ1837	SNSB, Zoologische Staatssammlung Muenchen
Eulithis populata	GBLAC872-13	BC ZSM Lep 77448	MG522558	BOLD:ABZ1837	SNSB, Zoologische Staatssammlung Muenchen
Eulithis populata	LEALT401-16	TLMF Lep 20624	MG522123	BOLD:ABZ1837	Tiroler Landesmuseum
Eulithis populata	NOLEP045-14	BCZMBNLep0045	MG522561	BOLD:ABZ1837	University of Bergen, Natural History Collections
Eulithis populata	LEFIB293-10	MM00797	HM871194	BOLD:ABZ1837	University of Oulu
Eulithis populata	GBLAC856-13	BC ZSM Lep 77432	MG522572	BOLD:ABZ1837	SNSB, Zoologische Staatssammlung Muenchen
Eulithis populata	PHLAC439-10	TLMF Lep 02474	JF860015	BOLD:ABZ1837	Tiroler Landesmuseum

Eulithis prunata	GBLAB248-13	BC ZSM Lep 75684	MG522765	BOLD:AAC1579	SNSB, Zoologische Staatssammlung Muenchen
Eulithis prunata	GWOR4092-09	BC ZSM Lep 21376	HQ601245	BOLD:AAC1579	SNSB, Zoologische Staatssammlung Muenchen
Eulithis prunata	ABOLB632-15	KLM Lep 02817	MG522481	BOLD:AAC1579	Landesmuseum Kaernten
Eulithis prunata	ABOLA439-14	TLMF Lep 16126	MG522775	BOLD:AAC1579	Tiroler Landesmuseum
Eulithis prunata	LEATD529-13	TLMF Lep 13176	MG522832	BOLD:AAC1579	Tiroler Landesmuseum
Eulithis prunata	GBLAA1345-15	BC ZSM Lep 87041	MG522532	BOLD:AAC1579	SNSB, Zoologische Staatssammlung Muenchen
Eulithis prunata	LEALT329-16	TLMF Lep 20552	MG522709	BOLD:ABZ7875	Tiroler Landesmuseum
Eulithis prunata	GMGML062-14	BIOUG17228-E10	MG522091	BOLD:AAC1579	Zoologisches Forschungsmuseum Alexander Koenig
Eulithis prunata	GWORC021-07	BC ZSM Lep 01243	HQ601246	BOLD:AAC1579	SNSB, Zoologische Staatssammlung Muenchen
Eulithis prunata	LEALT429-16	TLMF Lep 20652	MG522068	BOLD:ABZ7875	Tiroler Landesmuseum
Eulithis prunata	GWORG179-08	BC ZSM Lep 03657	JF415231	BOLD:AAC1579	SNSB, Zoologische Staatssammlung Muenchen
Eulithis prunata	PHLAF304-11	TLMF Lep 05474	MG522645	BOLD:ABA2102	Tiroler Landesmuseum
Eulithis prunata	GWORG049-08	BC ZSM Lep 02869	JF415230	BOLD:AAC1579	SNSB, Zoologische Staatssammlung Muenchen
Eulithis prunata	GWORC1505-08	BC ZSM Lep 01505	HQ601247	BOLD:AAC1579	SNSB, Zoologische Staatssammlung Muenchen
Eulithis prunata	ABOLA383-14	TLMF Lep 16070	MG522841	BOLD:AAC1579	Tiroler Landesmuseum
Eulithis prunata	LASTS040-14	TLMF Lep 14492	KP253428	BOLD:AAC1579	Tiroler Landesmuseum
Eulithis prunata	LEATD528-13	TLMF Lep 13175	MG522783	BOLD:AAC1579	Naturmuseum Suedtirol
Eulithis prunata	GMGMJ051-14	BIOUG17068-C06	MG522631	BOLD:AAC1579	Zoologisches Forschungsmuseum Alexander Koenig
Eulithis prunata	LEALT405-16	TLMF Lep 20628	MG522720	BOLD:ABZ7875	Tiroler Landesmuseum
Eulithis prunata	LEFIB294-10	MM00798	HM871195	BOLD:AAC1581	University of Oulu
Eulithis prunata	LEFIA429-10	MM01492	HM386770	BOLD:AAC1581	University of Oulu
Eulithis prunata	GBLAC134-13	BC ZSM Lep 66875	MG522188	BOLD:AAC1579	SNSB, Zoologische Staatssammlung Muenchen
Eulithis prunata	GWOSZ1002-11	BC ZSM Lep 62803	KX044782	BOLD:AAC1579	ZSM Collection of Manfred Sommerer
Eulithis prunata	GBLAD378-14	BC ZSM Lep 81894	MG522161	BOLD:AAC1579	SNSB, Zoologische Staatssammlung Muenchen
Eulithis prunata	GBLAC133-13	BC ZSM Lep 66874	MG522442	BOLD:AAC1579	SNSB, Zoologische Staatssammlung Muenchen
Eulithis prunata	GWORE2013-09	BC ZSM Lep 22411	GU654919	BOLD:AAC1579	SNSB, Zoologische Staatssammlung Muenchen
Eulithis prunata	LEFIA428-10	MM01491	HM386769	BOLD:AAC1581	University of Oulu
Eulithis testata	LEFIA347-10	MM01394	HM386690	BOLD:ABZ0682	University of Oulu
Eulithis testata	NOLEP066-14	BCZMBNLep0066	MG522022	BOLD:ABZ0682	University of Bergen, Natural History Collections
Eulithis testata	GWOSA789-10	BC ZSM Lep 36655	KX040208	BOLD:ABZ0682	SNSB, Zoologische Staatssammlung Muenchen

Eulithis testata	LEFIB291-10	MM00794	HM871192	BOLD:ABZ0682	University of Oulu
Eulithis testata	LEALT336-16	TLMF Lep 20559	MG522035	BOLD:ABZ0682	Tiroler Landesmuseum
Eulithis testata	GBLAB1740-14	BC ZSM Lep 76985	MG522695	BOLD:ABZ0682	Zentrum fur Biodokumentation des Saarlandes
Eulithis testata	LEFIA346-10	MM01393	HM386689	BOLD:ABZ0682	University of Oulu
Eulithis testata	LEALT335-16	TLMF Lep 20558	MG522347	BOLD:ABZ0682	Tiroler Landesmuseum
Eumedonia eumedon	LEFIC009-10	MM03212	HM871879	BOLD:AAC3364	University of Oulu
Eumedonia eumedon	PHLAB311-10	TLMF Lep 01111	HQ968475	BOLD:AAC3364	Tiroler Landesmuseum
Eumedonia eumedon	EZROM604-08	RV-06-M828	HQ004471	BOLD:AAC3364	Institut de Biologia Evolutiva (CSIC-UPF)
Eumedonia eumedon	PHLAF433-11	TLMF Lep 05603	MG522260	BOLD:AAC3364	Tiroler Landesmuseum
Eumedonia eumedon	LEFIJ515-10	MM17140	JF853637	BOLD:AAC3364	University of Oulu
Eumedonia eumedon	LASTS338-14	TLMF Lep 14790	KP253476	BOLD:AAC3364	Tiroler Landesmuseum
Eumedonia eumedon	GBLAA1412-15	BC ZSM Lep 87108	MG522142	BOLD:AAC3364	SNSB, Zoologische Staatssammlung Muenchen
Eumedonia eumedon	LOWA770-06	2005-LOWA-770	FJ663552	BOLD:AAC3364	McGuire Centre for Lepidoptera and Biodiversity
Eumedonia eumedon	LEATG224-14	TLMF Lep 14011	MG522654	BOLD:AAC3364	Tiroler Landesmuseum
Eumedonia eumedon	PHLAB310-10	TLMF Lep 01110	HQ968474	BOLD:AAC3364	Tiroler Landesmuseum
Eumedonia eumedon	EZROM051-08	RV-06-K670	HQ004472	BOLD:AAC3364	Institut de Biologia Evolutiva (CSIC-UPF)
Eumedonia eumedon	LOWA470-06	2005-LOWA-470	FJ663553	BOLD:AAC3364	McGuire Centre for Lepidoptera and Biodiversity
Eumedonia eumedon	GBLAA1410-15	BC ZSM Lep 87106	MG522721	BOLD:AAC3364	SNSB, Zoologische Staatssammlung Muenchen
Eumedonia eumedon	LEATD297-13	TLMF Lep 12944	MG522171	BOLD:AAC3364	Tiroler Landesmuseum
Eumedonia eumedon	LEFIG228-10	MM14128	HM875908	BOLD:AAC3364	University of Oulu
Eumedonia eumedon	PHLAW046-13	TLMF Lep 09843	KM572165	BOLD:AAC3364	Tiroler Landesmuseum
Eumedonia eumedon	EZROM052-08	RV-06-K675	HQ004473	BOLD:AAC3364	Institut de Biologia Evolutiva (CSIC-UPF)
Eumedonia eumedon	PHLAB358-10	TLMF Lep 01158	HQ968517	BOLD:AAC3364	Tiroler Landesmuseum
Eumedonia eumedon	LEATD150-13	TLMF Lep 12797	MG522700	BOLD:AAC3364	Tiroler Landesmuseum
Eumedonia eumedon	PHLAI501-13	TLMF Lep 09063	MG522147	BOLD:AAC3364	Tiroler Landesmuseum
Eumedonia eumedon	PHLSA697-11	TLMF Lep 06152	KM573528	BOLD:AAC3364	Tiroler Landesmuseum
Eumedonia eumedon	EZROM662-08	RV-06-K674	HQ004470	BOLD:AAC3364	Institut de Biologia Evolutiva (CSIC-UPF)
Eumedonia eumedon	LEALT368-16	TLMF Lep 20591	MG522545	BOLD:AAC3364	Tiroler Landesmuseum
Eumedonia eumedon	LEALT367-16	TLMF Lep 20590	MG522299	BOLD:AAC3364	Tiroler Landesmuseum
Eumedonia eumedon	LEALT369-16	TLMF Lep 20592	MG522342	BOLD:AAC3364	Tiroler Landesmuseum

Euphyia unangulata	LEALT552-16	TLMF Lep 20775	MG522713	BOLD:AAA4559	Tiroler Landesmuseum
Euphyia unangulata	GBLAA1247-15	BC ZSM Lep 87133	MG522046	BOLD:AAA4559	SNSB, Zoologische Staatssammlung Muenchen
Euphyia unangulata	GWORM144-09	BC ZSM Lep 24193	GU687212	BOLD:AAA4559	Research Collection of Alfred Haslberger
Euphyia unangulata	ABOLB630-15	KLM Lep 02815	MG522718	BOLD:AAA4559	Landesmuseum Kaernten
Euphyia unangulata	ABOLB281-15	TLMF Lep 17286	MG522643	BOLD:AAA4559	Tiroler Landesmuseum
Euphyia unangulata	LEFIE700-10	MM09682	HM874421	BOLD:AAA4559	University of Oulu
Euphyia unangulata	GBLAF737-14	BC ZSM Lep 82823	MG522568	BOLD:AAA4559	SNSB, Zoologische Staatssammlung Muenchen
Euphyia unangulata	GBLAC1022-13	BC ZSM Lep 76553	MG522380	BOLD:AAA4559	SNSB, Zoologische Staatssammlung Muenchen
Euphyia unangulata	FBLMV441-09	BC ZSM Lep 28421	GU707363	BOLD:AAA4559	Research Collection of Theo Gruenewald
Euphyia unangulata	LEFIB858-10	MM02880	HM871735	BOLD:AAA4559	University of Oulu
Euphyia unangulata	LEALT551-16	TLMF Lep 20774	MG522357	BOLD:AAA4559	Tiroler Landesmuseum
Euphyia unangulata	GBLAD719-14	BC ZSM Lep 84230	MG522522	BOLD:AAA4559	SNSB, Zoologische Staatssammlung Muenchen
Euphyia unangulata	GBLAB264-13	BC ZSM Lep 75700	MG522767	BOLD:AAA4559	SNSB, Zoologische Staatssammlung Muenchen
Euphyia unangulata	LEFIA406-10	MM01466	HM386748	BOLD:AAA4559	University of Oulu
Euphyia unangulata	GBLAF230-14	BC ZSM Lep 81936	MG522683	BOLD:AAA4559	SNSB, Zoologische Staatssammlung Muenchen
Euphyia unangulata	GBLAA437-14	BC ZSM Lep 80528	MG522457	BOLD:AAA4559	SNSB, Zoologische Staatssammlung Muenchen
Eupithecia pusillata	NOLEP050-14	BCZMBNLep0050	MG521936	BOLD:ABZ6329	University of Bergen, Natural History Collections
Eupithecia pusillata	PHLAI277-13	TLMF Lep 08772	KM572696	BOLD:ABZ6329	Tiroler Landesmuseum
Eupithecia pusillata	LEFIJ1730-13	MM23017	KM572758	BOLD:ABZ6329	University of Oulu
Eupithecia pusillata	LEATB315-13	TLMF Lep 10492	MG522128	BOLD:ABZ6329	Naturmuseum Suedtirol
Eupithecia pusillata	PHLAI278-13	TLMF Lep 08773	KM572224	BOLD:ABZ6329	Tiroler Landesmuseum
Eupithecia pusillata	LEFIJ1725-13	MM23012	KM572349	BOLD:ABZ6329	University of Oulu
Eupithecia pusillata	LEATB765-13	TLMF Lep 10942	MG522514	BOLD:ABZ6329	Naturmuseum Suedtirol
Eupithecia pusillata	PHLAF390-11	TLMF Lep 05560	MG522779	BOLD:ABZ6329	Tiroler Landesmuseum
Eupithecia pusillata	LEFIJ1734-13	MM23021	KM572594	BOLD:ABZ6329	University of Oulu
Eupithecia pusillata	GWORG155-08	BC ZSM Lep 03633	JF415242	BOLD:ABZ6329	SNSB, Zoologische Staatssammlung Muenchen
Eupithecia pusillata	LEFIE127-10	MM08340	HM873875	BOLD:ABZ6329	University of Oulu
Eupithecia pusillata	LEATD400-13	TLMF Lep 13047	MG522391	BOLD:ABZ6329	Tiroler Landesmuseum
Eupithecia pusillata	LEFIJ1732-13	MM23019	KM572378	BOLD:ABZ6329	University of Oulu
Eupithecia pusillata	LEFIE045-10	MM08143	HM873793	BOLD:ABZ6329	University of Oulu

Eupithecia pusillata	LEFIB288-10	MM00781	HM871189	BOLD:ABZ6329	University of Oulu
Eupithecia pusillata	ABOLB656-15	KLM Lep 02841	MG522297	BOLD:ABZ6329	Landesmuseum Kaernten
Eupithecia pusillata	NOLEP048-14	BCZMBNLep0048	MG521953	BOLD:ABZ6329	University of Bergen, Natural History Collections
Eupithecia pusillata	ABOLA927-15	TLMF Lep 16887	MG522578	BOLD:ABZ6329	Tiroler Landesmuseum
Eupithecia pusillata	LEATB766-13	TLMF Lep 10943	MG522199	BOLD:ABZ6329	Tiroler Landesmuseum
Eupithecia pusillata	LEFIJ1726-13	MM23013	KM573291	BOLD:ABZ6329	University of Oulu
Eupithecia pusillata	GWORL550-09	BC ZSM Lep 22832	GU686764	BOLD:ABZ6329	Research Collection of Alfred Haslberger
Eupithecia pusillata	LEATD639-13	TLMF Lep 13286	MG522318	BOLD:ABZ6329	Tiroler Landesmuseum
Eupithecia pusillata	NOLEP049-14	BCZMBNLep0049	MG522555	BOLD:ABZ6329	University of Bergen, Natural History Collections
Eupithecia pusillata	LEATD638-13	TLMF Lep 13285	MG522800	BOLD:ABZ6329	Tiroler Landesmuseum
Eupithecia pusillata	PHLAC597-10	TLMF Lep 02632	JF860164	BOLD:ABZ6329	Tiroler Landesmuseum
Eupithecia pusillata	LEFIJ1731-13	MM23018	KM573261	BOLD:ABZ6329	University of Oulu
Eupithecia pusillata	PHLAI303-13	TLMF Lep 08798	KM573465	BOLD:ABZ6329	Tiroler Landesmuseum
Eupithecia pusillata	LEFIJ1729-13	MM23016	KM572837	BOLD:ABZ6329	University of Oulu
Eupithecia pusillata	LEFIA659-10	MM01786	HM870907	BOLD:ABZ6329	University of Oulu
Eupithecia pusillata	PHLAF284-11	TLMF Lep 05454	MG522080	BOLD:ABZ6329	Tiroler Landesmuseum
Eupithecia pusillata	LEFIJ1724-13	MM23011	KM572206	BOLD:ABZ6329	University of Oulu
Eupithecia pusillata	LEFIJ1733-13	MM23020	KM573185	BOLD:ABZ6329	University of Oulu
Eupithecia pusillata	LEFIA658-10	MM01785	HM870906	BOLD:ABZ6329	University of Oulu
Eupithecia pusillata	LEFIJ588-10	MM17213	KM573453	BOLD:ABZ6329	University of Oulu
Eupithecia pusillata	LEATB827-13	TLMF Lep 11004	MG522041	BOLD:ABZ6329	Tiroler Landesmuseum
Eupithecia pusillata	GWORL549-09	BC ZSM Lep 22831	GU686771	BOLD:ABZ6329	Research Collection of Alfred Haslberger
Eupithecia pusillata	LEFIJ1723-13	MM23010	KM572214	BOLD:ABZ6329	University of Oulu
Eupithecia pusillata	LEALT539-16	TLMF Lep 20762	MG522664	BOLD:ABZ6329	Tiroler Landesmuseum
Eupithecia pusillata	LEALT540-16	TLMF Lep 20763	MG522346	BOLD:ABZ6329	Tiroler Landesmuseum
Eupithecia pusillata	LEALT538-16	TLMF Lep 20761	MG522739	BOLD:ABZ6329	Tiroler Landesmuseum
Eurois occulta	LEATB449-13	TLMF Lep 10626	MF921694	BOLD:AAA3312	Tiroler Landesmuseum
Eurois occulta	LEALT324-16	TLMF Lep 20547	MG522065	BOLD:AAA3312	Tiroler Landesmuseum
Eurois occulta	PHLAC426-10	TLMF Lep 02461	JF860002	BOLD:AAA3312	Tiroler Landesmuseum
Eurois occulta	GWORZ520-10	BC ZSM Lep 31731	HM914228	BOLD:AAA3312	Research Collection of Alfred Haslberger

Eurois occulta	LEATD097-13	TLMF Lep 12744	MF921237	BOLD:AAA3312	Tiroler Landesmuseum
Eurois occulta	LEFIB831-10	MM02786	HM871708	BOLD:AAA3312	University of Oulu
Eurois occulta	LEALT398-16	TLMF Lep 20621	MG522780	BOLD:AAA3312	Tiroler Landesmuseum
Eurois occulta	GWORL332-09	BC ZSM Lep 22044	GU686935	BOLD:AAA3312	SNSB, Zoologische Staatssammlung Muenchen
Eurois occulta	FBLMV041-09	BC ZSM Lep 28021	HQ955370	BOLD:AAA3312	SNSB, Zoologische Staatssammlung Muenchen
Eurois occulta	FGMLD017-13	BC ZSM Lep 78873	MF922073	BOLD:AAA3312	Research Collection of Rudolf Tannert
Eurois occulta	PHLAA251-09	TLMF Lep 00291	HM425790	BOLD:AAA3312	Tiroler Landesmuseum
Eurois occulta	GWORO849-09	BC ZSM Lep 30541	GU688482	BOLD:AAA3312	SNSB, Zoologische Staatssammlung Muenchen
Eurois occulta	LEATD161-13	TLMF Lep 12808	MF921608	BOLD:AAA3312	Tiroler Landesmuseum
Eurois occulta	GBLAC466-13	BC ZSM Lep 77707	MF921700	BOLD:AAA3312	SNSB, Zoologische Staatssammlung Muenchen
Eurois occulta	PHLAA209-09	TLMF Lep 00249	HM425758	BOLD:AAA3312	Tiroler Landesmuseum
Eurois occulta	ABOLB524-15	KLM Lep 02709	MG522096	BOLD:AAA3312	Landesmuseum Kaernten
Eurois occulta	GMFIT048-13	BIOUG04118-F08	KM572179	BOLD:AAA3312	Centre for Biodiversity Genomics
Eurois occulta	LEALT399-16	TLMF Lep 20622	MG522158	BOLD:AAA3312	Tiroler Landesmuseum
Eurois occulta	LEFIA583-10	MM01688	HM870832	BOLD:AAA3312	University of Oulu
Eurois occulta	LEFIA582-10	MM01687	HM870831	BOLD:AAA3312	University of Oulu
Euxoa recussa	PHLAC589-10	TLMF Lep 02624	JF860156	BOLD:ACE9579	Tiroler Landesmuseum
Euxoa recussa	LEFIB258-10	MM00730	HM871161	BOLD:ACE9579	University of Oulu
Euxoa recussa	LEATD621-13	TLMF Lep 13268	MG522136	BOLD:ACE9579	Tiroler Landesmuseum
Euxoa recussa	GWORZ496-10	BC ZSM Lep 31707	HM914206	BOLD:ACE9579	Research Collection of Alfred Haslberger
Euxoa recussa	LEFIK426-10	MM18001	JF854055	BOLD:ACE9579	University of Oulu
Euxoa recussa	LEFIB259-10	MM00731	HM871162	BOLD:ACE9579	University of Oulu
Euxoa recussa	DEEUR567-15	TLMF Lep 17762	MG522196	BOLD:ACE9579	Research Collection of Peter Buchner
Euxoa recussa	LEALT298-16	TLMF Lep 20521	MG522494	BOLD:ACE9579	Tiroler Landesmuseum
Euxoa recussa	LEALT299-16	TLMF Lep 20522	MG522463	BOLD:ACE9579	Tiroler Landesmuseum
Euxoa recussa	LEATD416-13	TLMF Lep 13063	MG522025	BOLD:ACE9579	Tiroler Landesmuseum
Euxoa recussa	LEALT297-16	TLMF Lep 20520	MG522537	BOLD:ACE9579	Tiroler Landesmuseum
Euxoa recussa	PHLSA642-11	TLMF Lep 06097	KM572912	BOLD:ACE9579	Tiroler Landesmuseum
Euxoa recussa	LEATG146-14	TLMF Lep 13933	MG522717	BOLD:ACE9579	Tiroler Landesmuseum
Euxoa recussa	PHLAH845-12	TLMF Lep 08649	MG522517	BOLD:ACE9579	Tiroler Landesmuseum

Euxoa recussa	LEATB752-13	TLMF Lep 10929	MG522451	BOLD:ACE9579	Naturmuseum Suedtirol
Gazoryctra ganna	LEFIJ892-10	MM17517	JX034573	BOLD:AAI8414	University of Oulu
Gazoryctra ganna	PHLAB1016-10	TLMF Lep 01816	HQ968280	BOLD:ADE0116	Tiroler Landesmuseum
Gazoryctra ganna	LEALT288-16	TLMF Lep 20511	MG522576	BOLD:ADE0255	Tiroler Landesmuseum
Gazoryctra ganna	PHLAB1015-10	TLMF Lep 01815	JF859593	BOLD:ADE0116	Tiroler Landesmuseum
Gazoryctra ganna	PHLAD076-11	TLMF Lep 03061	JN307292	BOLD:ADE0116	Tiroler Landesmuseum
Gazoryctra ganna	LEFIF617-10	MM12505	HM875301	BOLD:AAI8414	University of Oulu
Gazoryctra ganna	LEALT286-16	TLMF Lep 20509	MG522056	BOLD:ADE0255	Tiroler Landesmuseum
Gazoryctra ganna	LEFIF618-10	MM12506	HM875302	BOLD:AAI8414	University of Oulu
Graphiphora augur	GWORK476-09	BC ZSM Lep 21806	JF415549	BOLD:ACF0935	SNSB, Zoologische Staatssammlung Muenchen
Graphiphora augur	FBLMV017-09	BC ZSM Lep 27997	HQ955358	BOLD:ACF0935	SNSB, Zoologische Staatssammlung Muenchen
Graphiphora augur	LEALT483-16	TLMF Lep 20706	MG521945	BOLD:ACF0935	Tiroler Landesmuseum
Graphiphora augur	LEFIC755-10	MM04870	HM872574	BOLD:ACF0935	University of Oulu
Graphiphora augur	GBLAC946-13	BC ZSM Lep 77522	MG522443	BOLD:ACF0935	SNSB, Zoologische Staatssammlung Muenchen
Graphiphora augur	LEATB535-13	TLMF Lep 10712	MG522512	BOLD:ACF0935	Naturmuseum Suedtirol
Graphiphora augur	LEALT482-16	TLMF Lep 20705	MG522611	BOLD:ACF0935	Tiroler Landesmuseum
Graphiphora augur	LEFIE019-10	MM08074	HM873768	BOLD:ACF0935	University of Oulu
Graphiphora augur	ABOLB779-15	KLM Lep 03439	MG522796	BOLD:ACF0935	Landesmuseum Kaernten
Graphiphora augur	PHLSA645-11	TLMF Lep 06100	KM573629	BOLD:ACF0935	Tiroler Landesmuseum
Graphiphora augur	LEATD102-13	TLMF Lep 12749	MG522396	BOLD:ACF0935	Tiroler Landesmuseum
Graphiphora augur	LEFIB820-10	MM02757	HM871697	BOLD:ACF0935	University of Oulu
Graphiphora augur	LEALT484-16	TLMF Lep 20707	MG522772	BOLD:ACF0935	Tiroler Landesmuseum
Graphiphora augur	LEATB804-13	TLMF Lep 10981	MG521989	BOLD:ACF0935	Tiroler Landesmuseum
Graphiphora augur	LEATD451-13	TLMF Lep 13098	MG522144	BOLD:ACF0935	Tiroler Landesmuseum
Gypsonoma nitidulana	LEFIC513-10	MM04221	HM872337	BOLD:AAB1285	University of Oulu
Gypsonoma nitidulana	LEFIF862-10	MM13234	HM875544	BOLD:AAB1285	University of Oulu
Gypsonoma nitidulana	LEFIK759-10	MM18334	JF854316	BOLD:AAB1285	University of Oulu
Gypsonoma nitidulana	LEFID439-10	MM06371	HQ570335	BOLD:AAB1285	University of Oulu
Gypsonoma nitidulana	LEFIB497-10	MM02078	HM871392	BOLD:AAB1285	University of Oulu
Gypsonoma nitidulana	LEFIF863-10	MM13235	HM875545	BOLD:AAB1285	University of Oulu

Gypsonoma nitidulana	LEFIJ149-10	MM10057	JF853462	BOLD:AAB1285	University of Oulu
Gypsonoma nitidulana	LEFIC371-10	MM03900	HM872214	BOLD:AAB1285	University of Oulu
Gypsonoma nitidulana	LEFID489-10	MM06442	HM873254	BOLD:AAB1285	University of Oulu
Gypsonoma nitidulana	LEFIG178-10	MM14045	HM875858	BOLD:AAB1285	University of Oulu
Gypsonoma nitidulana	LEFIE130-10	MM08346	HM873878	BOLD:AAB1285	University of Oulu
Gypsonoma nitidulana	LEALT075-16	TLMF Lep 20298	MG522308	BOLD:AAB1285	Tiroler Landesmuseum
Gypsonoma nitidulana	LEALT074-16	TLMF Lep 20297	MG522430	BOLD:AAB1285	Tiroler Landesmuseum
Gypsonoma nitidulana	LEFIJ143-10	MM09988	JF853456	BOLD:AAB1285	University of Oulu
Gypsonoma nitidulana	LEFIB498-10	MM02079	HM871393	BOLD:AAB1285	University of Oulu
Gypsonoma nitidulana	LEFIK758-10	MM18333	JF854315	BOLD:AAB1285	University of Oulu
Gypsonoma nitidulana	LEFIK757-10	MM18332	JF854314	BOLD:AAB1285	University of Oulu
Gypsonoma nitidulana	PHLAI904-13	KLM Lep 01094	MG522241	BOLD:AAB1285	Landesmuseum Kaernten
Hadena compta	FBLMX177-11	BC ZSM Lep 37658	JN284038	BOLD:AAC8417	SNSB, Zoologische Staatssammlung Muenchen
Hadena compta	LEALT396-16	TLMF Lep 20619	MG522573	BOLD:AAC8417	Tiroler Landesmuseum
Hadena compta	LEATB473-13	TLMF Lep 10650	MG522778	BOLD:AAC8417	Tiroler Landesmuseum
Hadena compta	PHLSA575-11	TLMF Lep 06030	MG522753	BOLD:AAC8417	Tiroler Landesmuseum
Hadena compta	PHLAC557-10	TLMF Lep 02592	JF860126	BOLD:AAC8417	Tiroler Landesmuseum
Hadena compta	PHLAB1188-10	TLMF Lep 01988	HQ968398	BOLD:AAC8417	Tiroler Landesmuseum
Hadena compta	LEALT395-16	TLMF Lep 20618	MG522298	BOLD:AAC8417	Tiroler Landesmuseum
Hadena compta	ABOLB516-15	KLM Lep 02701	MG522012	BOLD:AAC8417	Landesmuseum Kaernten
Hadena compta	PHLAA470-09	TLMF Lep 00510	HM425982	BOLD:AAC8417	Tiroler Landesmuseum
Hadena compta	PHLAD071-11	TLMF Lep 03056	JN284159	BOLD:AAC8417	Tiroler Landesmuseum
Hadena compta	LEFIC628-10	MM04570	HM872449	BOLD:AAC8417	University of Oulu
Hadena compta	LEATB401-13	TLMF Lep 10578	MG522017	BOLD:AAC8417	Tiroler Landesmuseum
Hadena compta	PHLSA448-11	TLMF Lep 05903	MG522411	BOLD:AAC8417	Tiroler Landesmuseum
Hadena compta	LEALT394-16	TLMF Lep 20617	MG522300	BOLD:AAC8417	Tiroler Landesmuseum
Hadena compta	LEFIF544-10	MM12225	HM875229	BOLD:AAC8417	University of Oulu
Hadena compta	GWORK430-09	BC ZSM Lep 21760	JF415553	BOLD:AAC8417	SNSB, Zoologische Staatssammlung Muenchen
Hadena compta	GWOR3901-09	BC ZSM Lep 21185	JF415554	BOLD:AAC8417	SNSB, Zoologische Staatssammlung Muenchen
Hadena compta	LEFIC771-10	MM04898	HM872590	BOLD:AAC8417	University of Oulu

Hadena compta	FBLMV067-09	BC ZSM Lep 28047	HM902074		SNSB, Zoologische Staatssammlung Muenchen
Lasionycta imbecilla	LEATD484-13	TLMF Lep 13131	MG522685	BOLD:AAE1902	Tiroler Landesmuseum
Lasionycta imbecilla	LEFID594-10	MM06604	HM873359	BOLD:AAE1902	University of Oulu
Lasionycta imbecilla	LEATG311-14	TLMF Lep 14098	MG522597	BOLD:AAE1902	Tiroler Landesmuseum
Lasionycta imbecilla	GWORO705-09	BC ZSM Lep 27641	JF415546	BOLD:AAE1902	SNSB, Zoologische Staatssammlung Muenchen
Lasionycta imbecilla	LEALT409-16	TLMF Lep 20632	MG522728	BOLD:AAE1902	Tiroler Landesmuseum
Lasionycta imbecilla	LEATJ241-15	TLMF Lep 18671	MG522348	BOLD:AAE1902	Tiroler Landesmuseum
Lasionycta imbecilla	LEALT459-16	TLMF Lep 20682	MG521998	BOLD:AAE1902	Tiroler Landesmuseum
Lasionycta imbecilla	PHLSA653-11	TLMF Lep 06108	KM572959	BOLD:AAE1902	Tiroler Landesmuseum
Lasionycta imbecilla	LEFIA527-10	MM01610	KM571992	BOLD:AAE1902	University of Oulu
Lasionycta imbecilla	GWORZ486-10	BC ZSM Lep 31697	HM914196	BOLD:AAE1902	Research Collection of Alfred Haslberger
Lasionycta imbecilla	LEALT473-16	TLMF Lep 20696	MG522465	BOLD:AAE1902	Tiroler Landesmuseum
Lasionycta imbecilla	ABOLA034-14	KLM Lep 01554	MG522172	BOLD:AAE1902	Landesmuseum Kaernten
Lasionycta imbecilla	PHLAE360-11	TLMF Lep 04675	JN272314	BOLD:AAE1902	Tiroler Landesmuseum
Lasionycta imbecilla	LEFIA526-10	MM01609	KM573003	BOLD:AAE1902	University of Oulu
Lasionycta imbecilla	LEATJ249-15	TLMF Lep 18679	MG522085	BOLD:AAE1902	Tiroler Landesmuseum
Lasionycta imbecilla	PHLAH728-12	TLMF Lep 08547	KM572748	BOLD:AAE1902	inatura, Dornbirn
Lasionycta imbecilla	PHLAA384-09	TLMF Lep 00424	HM425904	BOLD:AAE1902	Tiroler Landesmuseum
Lasionycta proxima	PHLAA192-09	TLMF Lep 00232	HM425741	BOLD:AAD3486	Tiroler Landesmuseum
Lasionycta proxima	LEFIE768-10	MM09917	HM874487	BOLD:AAD3486	University of Oulu
Lasionycta proxima	FBLMV072-09	BC ZSM Lep 28052	GU707452	BOLD:AAD3486	SNSB, Zoologische Staatssammlung Muenchen
Lasionycta proxima	GWOSA399-10	BC ZSM Lep 34460	HQ565350	BOLD:AAD3486	Research Collection of Alfred Haslberger
Lasionycta proxima	ABOLA035-14	KLM Lep 01555	MG522574	BOLD:AAD3486	Landesmuseum Kaernten
Lasionycta proxima	PHLAI282-13	TLMF Lep 08777	KM573385	BOLD:AAD3486	Tiroler Landesmuseum
Lasionycta proxima	PHLAF432-11	TLMF Lep 05602	MG522032	BOLD:AAD3486	Tiroler Landesmuseum
Lasionycta proxima	PHLAF422-11	TLMF Lep 05592	MG522259	BOLD:AAD3486	Tiroler Landesmuseum
Lasionycta proxima	LEFIH026-10	MM15890	HM876662	BOLD:AAD3486	University of Oulu
Lasionycta proxima	LEATD104-13	TLMF Lep 12751	MG522725	BOLD:AAD3486	Tiroler Landesmuseum
Lasionycta proxima	PHLAH821-12	TLMF Lep 08625	MG522189	BOLD:AAD3486	Tiroler Landesmuseum
Lasionycta proxima	PHLAC453-10	TLMF Lep 02488	JF860028	BOLD:AAD3486	Tiroler Landesmuseum

Lasionycta proxima	LEFIH027-10	MM15891	HM876663	BOLD:AAD3486	University of Oulu
Lasionycta proxima	PHLAA299-09	TLMF Lep 00339	HM425831	BOLD:AAD3486	Tiroler Landesmuseum
Lasionycta proxima	LEATD105-13	TLMF Lep 12752	MG522647	BOLD:AAD3486	Tiroler Landesmuseum
Lasionycta proxima	PHLAH805-12	TLMF Lep 08609	MG522776	BOLD:AAD3486	Tiroler Landesmuseum
Lasionycta proxima	LEALT410-16	TLMF Lep 20633	MG522372	BOLD:AAD3486	Tiroler Landesmuseum
Lasionycta proxima	LEALT411-16	TLMF Lep 20634	MG522074	BOLD:AAD3486	Tiroler Landesmuseum
Lasionycta proxima	LEALT412-16	TLMF Lep 20635	MG521966	BOLD:AAD3486	Tiroler Landesmuseum
Lasionycta proxima	LEALT461-16	TLMF Lep 20684	MG522084	BOLD:AAD3486	Tiroler Landesmuseum
Levipalpus hepatariella	LEATE473-13	TLMF Lep 11885	MG521924	BOLD:AAJ2744	Tiroler Landesmuseum
Levipalpus hepatariella	LEFIJ918-10	MM17543	JX034617	BOLD:AAJ2744	University of Oulu
Levipalpus hepatariella	PHLAB1008-10	TLMF Lep 01808	KX042195	BOLD:AAJ2744	Tiroler Landesmuseum
Levipalpus hepatariella	LEFIG580-10	MM14769	HM876251	BOLD:AAJ2744	University of Oulu
Levipalpus hepatariella	PHLAC704-10	TLMF Lep 02739	JF860259	BOLD:AAJ2744	Tiroler Landesmuseum
Levipalpus hepatariella	LEFIG581-10	MM14770	HM876252	BOLD:AAJ2744	University of Oulu
Levipalpus hepatariella	PHLAD538-11	TLMF Lep 03713	JN266666	BOLD:AAJ2744	Tiroler Landesmuseum
Levipalpus hepatariella	PHLAB1009-10	TLMF Lep 01809	KX042106	BOLD:AAJ2744	Tiroler Landesmuseum
Levipalpus hepatariella	DEEUR003-11	TLMF Lep 04793	JX034570	BOLD:AAJ2744	Research Collection of Peter Sonderegger
Levipalpus hepatariella	LEFIJ1222-11	MM21082	KT782381	BOLD:AAJ2744	University of Oulu
Levipalpus hepatariella	LEALT175-16	TLMF Lep 20398	MG522507	BOLD:AAJ2744	Tiroler Landesmuseum
Levipalpus hepatariella	LEALT015-16	TLMF Lep 20238	MG522724	BOLD:AAJ2744	Tiroler Landesmuseum
Levipalpus hepatariella	LEALT174-16	TLMF Lep 20397	MG522644	BOLD:AAJ2744	Tiroler Landesmuseum
Levipalpus hepatariella	LEALT247-16	TLMF Lep 20470	MG522350	BOLD:AAJ2744	Tiroler Landesmuseum
Lycaena virgaureae	GWOTL174-13	BC ZSM Lep 67105	MG522589	BOLD:AAB4984	SNSB, Zoologische Staatssammlung Muenchen
Lycaena virgaureae	LEFIJ496-10	MM17121	JF853620	BOLD:AAB4984	University of Oulu
Lycaena virgaureae	EZRMN064-08	RVcoll.06-V698	HQ004690	BOLD:AAB4984	Institut de Biologia Evolutiva (CSIC-UPF)
Lycaena virgaureae	PHLAF290-11	TLMF Lep 05460	MG522770	BOLD:AAB4984	Tiroler Landesmuseum
Lycaena virgaureae	GBLAF378-14	BC ZSM Lep 82084	MG522492	BOLD:AAB4984	SNSB, Zoologische Staatssammlung Muenchen
Lycaena virgaureae	PHLAI506-13	TLMF Lep 09068	MG522711	BOLD:AAB4984	Tiroler Landesmuseum
Lycaena virgaureae	EZROM271-08	RV-06-M879	HQ004695	BOLD:AAB4984	Institut de Biologia Evolutiva (CSIC-UPF)
Lycaena virgaureae	PHLAH830-12	TLMF Lep 08634	MG522639	BOLD:AAB4984	Tiroler Landesmuseum

Lycaena virgaureae	EZROM273-08	RV-07-E489	HQ004694	BOLD:AAB4984	Institut de Biologia Evolutiva (CSIC-UPF)
Lycaena virgaureae	EZROM677-08	RV-06-N002	HQ004692	BOLD:AAB4984	Institut de Biologia Evolutiva (CSIC-UPF)
Lycaena virgaureae	LEALT372-16	TLMF Lep 20595	MG522003	BOLD:AAB4984	Tiroler Landesmuseum
Lycaena virgaureae	PHLSA406-11	TLMF Lep 05861	MG522431	BOLD:AAB4984	Tiroler Landesmuseum
Lycaena virgaureae	EZRMN066-08	RVcoll.08-M590	HQ004688	BOLD:AAB4984	Institut de Biologia Evolutiva (CSIC-UPF)
Lycaena virgaureae	LEALT373-16	TLMF Lep 20596	MG522583	BOLD:AAB4984	Tiroler Landesmuseum
Lycaena virgaureae	LEATC621-13	TLMF Lep 12603	KM573555	BOLD:AAB4984	Tiroler Landesmuseum
Lycaena virgaureae	GBLAA1250-15	BC ZSM Lep 87136	MG522001	BOLD:AAB4984	SNSB, Zoologische Staatssammlung Muenchen
Lycaena virgaureae	EZROM272-08	RV-07-D518	HQ004693	BOLD:AAB4984	Institut de Biologia Evolutiva (CSIC-UPF)
Lycaena virgaureae	PHLAF291-11	TLMF Lep 05461	MG522399	BOLD:AAB4984	Tiroler Landesmuseum
Lycaena virgaureae	LEFIG533-10	MM14659	HM876206	BOLD:AAB4984	University of Oulu
Lycaena virgaureae	EZRMN065-08	RVcoll.07-C907	HQ004691	BOLD:AAB4984	Institut de Biologia Evolutiva (CSIC-UPF)
Lycaena virgaureae	GWORA2465-09	BC ZSM Lep 30677	HM393191	BOLD:AAB4984	Research Collection of Ralph Sturm
Lycaena virgaureae	LEALT374-16	TLMF Lep 20597	MG522352	BOLD:AAB4984	Tiroler Landesmuseum
Lycaena virgaureae	PHLSA405-11	TLMF Lep 05860	MG522825	BOLD:AAB4984	Tiroler Landesmuseum
Lycaena virgaureae	LEFIB038-10	MM00305	HM870947	BOLD:AAB4984	University of Oulu
Lycaena virgaureae	LEATA081-13	TLMF Lep 09498	MG522195	BOLD:AAB4984	Tiroler Landesmuseum
Lycaena virgaureae	GWOTL173-13	BC ZSM Lep 67104	MG521935	BOLD:AAB4984	SNSB, Zoologische Staatssammlung Muenchen
Lycaena virgaureae	LEATD146-13	TLMF Lep 12793	MG522584	BOLD:AAB4984	Tiroler Landesmuseum
Lycaena virgaureae	EZRMN063-08	RVcoll.06-M904	HQ004689		Institut de Biologia Evolutiva (CSIC-UPF)
Macaria brunneata	LEFIE023-10	MM08082	HM873772	BOLD:ABY9522	University of Oulu
Macaria brunneata	LEFIA396-10	MM01455	HM386738	BOLD:ABY9522	University of Oulu
Macaria brunneata	LEFIA397-10	MM01456	HM386739	BOLD:ABY9522	University of Oulu
Macaria brunneata	LEALT421-16	TLMF Lep 20644	MG522716	BOLD:ABY9522	Tiroler Landesmuseum
Macaria brunneata	GWORL440-09	BC ZSM Lep 22342	GU686856	BOLD:ABY9522	SNSB, Zoologische Staatssammlung Muenchen
Macaria brunneata	GWOR3786-09	BC ZSM Lep 21070	HQ601412	BOLD:ABY9522	SNSB, Zoologische Staatssammlung Muenchen
Macaria brunneata	PHLSA715-11	TLMF Lep 06170	KM572273	BOLD:ABY9522	Tiroler Landesmuseum
Macaria brunneata	LEALT488-16	TLMF Lep 20711	MG522049	BOLD:ABY9522	Tiroler Landesmuseum
Macaria brunneata	FBLMZ120-12	BC ZSM Lep 51376	KX040768	BOLD:ABY9522	SNSB, Zoologische Staatssammlung Muenchen
Macaria brunneata	GBLAB078-13	BC ZSM Lep 72569	MG522289	BOLD:ABY9522	SNSB, Zoologische Staatssammlung Muenchen

Macaria brunneata	LEATJ283-15	TLMF Lep 18713	MG522736	BOLD:ABY9522	Tiroler Landesmuseum
Macaria brunneata	PHLAC441-10	TLMF Lep 02476	JF860017	BOLD:ABY9522	Tiroler Landesmuseum
Macaria brunneata	GWORL437-09	BC ZSM Lep 22339	GU686861	BOLD:ABY9522	SNSB, Zoologische Staatssammlung Muenchen
Macaria brunneata	LEATC101-13	TLMF Lep 11228	MG522627	BOLD:ABY9522	Naturmuseum Suedtirol
Macaria brunneata	LEALT422-16	TLMF Lep 20645	MG522045	BOLD:ABY9522	Tiroler Landesmuseum
Macaria brunneata	ABOLB063-15	TLMF Lep 17068	MG522831	BOLD:ABY9522	Tiroler Landesmuseum
Macaria brunneata	GBLAF704-14	BC ZSM Lep 82790	MG522009	BOLD:ABY9522	SNSB, Zoologische Staatssammlung Muenchen
Macaria brunneata	GBLAF459-14	BC ZSM Lep 82165	MG522393	BOLD:ABY9522	SNSB, Zoologische Staatssammlung Muenchen
Macaria brunneata	GWORE2188-09	BC ZSM Lep 24011	GU654899	BOLD:ABY9522	Research Collection of Alfred Haslberger
Macaria brunneata	GBLGC120-12	BC ZSM Lep R 21070	MG522410	BOLD:ABY9522	SNSB, Zoologische Staatssammlung Muenchen
Matilella fusca	LEATF245-14	TLMF Lep 13557	MG522486	BOLD:AAA4759	Tiroler Landesmuseum
Matilella fusca	PHLAB083-10	TLMF Lep 00883	HQ968257	BOLD:AAA4759	Tiroler Landesmuseum
Matilella fusca	PHLAC043-10	TLMF Lep 02078	JF859667	BOLD:AAA4759	Tiroler Landesmuseum
Matilella fusca	PHLAW126-13	TLMF Lep 09923	KM573585	BOLD:AAA4759	inatura, Dornbirn
Matilella fusca	LEFIE782-10	MM09959	HM874501	BOLD:AAA4759	University of Oulu
Matilella fusca	PHLAB027-10	TLMF Lep 00827	HQ968203	BOLD:AAA4759	Tiroler Landesmuseum
Matilella fusca	LEFIE574-10	MM09468	HM874297	BOLD:AAA4759	University of Oulu
Matilella fusca	LEATF244-14	TLMF Lep 13556	MG522087	BOLD:AAA4759	Tiroler Landesmuseum
Matilella fusca	LEALT136-16	TLMF Lep 20359	MG522112	BOLD:AAA4759	Tiroler Landesmuseum
Matilella fusca	LEFID631-10	MM06652	HM873394	BOLD:AAA4759	University of Oulu
Matilella fusca	LEALT080-16	TLMF Lep 20303	MG522495	BOLD:AAA4759	Tiroler Landesmuseum
Matilella fusca	PHLAC044-10	TLMF Lep 02079	JF859668	BOLD:AAA4759	Tiroler Landesmuseum
Miltochrista miniata	FBLMZ115-12	BC ZSM Lep 51371	KX040216	BOLD:AAC4781	SNSB, Zoologische Staatssammlung Muenchen
Miltochrista miniata	LEFIA105-10	MM01028	HM396452	BOLD:AAC4781	University of Oulu
Miltochrista miniata	GWOTL184-13	BC ZSM Lep 67115	MG522340	BOLD:AAC4781	SNSB, Zoologische Staatssammlung Muenchen
Miltochrista miniata	PHLAV293-12	TLMF Lep 08112	KM572432	BOLD:AAC4781	inatura, Dornbirn
Miltochrista miniata	GBLAA394-14	BC ZSM Lep 80485	MG521988	BOLD:AAC4781	SNSB, Zoologische Staatssammlung Muenchen
Miltochrista miniata	LEALT524-16	TLMF Lep 20747	MG522808	BOLD:AAC4781	Tiroler Landesmuseum
Miltochrista miniata	GWOR4161-09	BC ZSM Lep 21445	JF415399	BOLD:AAC4781	SNSB, Zoologische Staatssammlung Muenchen

Miltochrista miniata	LEATC225-13	TLMF Lep 11352	MG522672	BOLD:AAC4781	Tiroler Landesmuseum
Miltochrista miniata	LEALT525-16	TLMF Lep 20748	MG522820	BOLD:AAC4781	Tiroler Landesmuseum
Miltochrista miniata	GWOTL140-13	BC ZSM Lep 67071	MG521976	BOLD:AAC4781	SNSB, Zoologische Staatssammlung Muenchen
Miltochrista miniata	LEATG178-14	TLMF Lep 13965	MG522535	BOLD:AAC4781	Tiroler Landesmuseum
Miltochrista miniata	PHLAC559-10	TLMF Lep 02594	JF860128	BOLD:AAC4781	Tiroler Landesmuseum
Miltochrista miniata	LEFIF382-10	MM11636	HM875067	BOLD:AAC4781	University of Oulu
Miltochrista miniata	GBLAF266-14	BC ZSM Lep 81972	MG522063	BOLD:AAC4781	SNSB, Zoologische Staatssammlung Muenchen
Miltochrista miniata	GWORK364-09	BC ZSM Lep 21599	JF415398	BOLD:AAC4781	SNSB, Zoologische Staatssammlung Muenchen
Miltochrista miniata	LEALT523-16	TLMF Lep 20746	MG522105	BOLD:AAC4781	Tiroler Landesmuseum
Miltochrista miniata	GWOTL138-13	BC ZSM Lep 67069	MG522412	BOLD:AAC4781	SNSB, Zoologische Staatssammlung Muenchen
Miltochrista miniata	GBLGC210-12	BC ZSM Lep R 21445	MG522605	BOLD:AAC4781	SNSB, Zoologische Staatssammlung Muenchen
Miltochrista miniata	ABOLC111-16	TLMF Lep 20144	MG522625	BOLD:AAC4781	Tiroler Landesmuseum
Miltochrista miniata	GBLAD117-14	BC ZSM Lep 78688	MG522073	BOLD:AAC4781	SNSB, Zoologische Staatssammlung Muenchen
Miltochrista miniata	LEFIA104-10	MM01027	HM396451	BOLD:AAC4781	University of Oulu
Miltochrista miniata	GBLAB626-13	BC ZSM Lep 75397	MG522026	BOLD:AAC4781	SNSB, Zoologische Staatssammlung Muenchen
Miltochrista miniata	GWOTL139-13	BC ZSM Lep 67070	MG522400	BOLD:AAC4781	SNSB, Zoologische Staatssammlung Muenchen
Miltochrista miniata	GWOTL183-13	BC ZSM Lep 67114	MG522817	BOLD:AAC4781	SNSB, Zoologische Staatssammlung Muenchen
Miltochrista miniata	ABOLA123-14	KLM Lep 01643	MG522231	BOLD:AAC4781	Landesmuseum Kaernten
Miltochrista miniata	GBLAC582-13	BC ZSM Lep 78203	MG522737	BOLD:AAC4781	SNSB, Zoologische Staatssammlung Muenchen
Miltochrista miniata	FBLMV289-09	BC ZSM Lep 28269	GU707343	BOLD:AAC4781	Research Collection of Theo Gruenewald
Miltochrista miniata	LEATD597-13	TLMF Lep 13244	MG522130	BOLD:AAC4781	Tiroler Landesmuseum
Mompha locupletella	LEALT255-16	TLMF Lep 20478	MG522599	BOLD:AAF2616	Tiroler Landesmuseum
Mompha locupletella	LEATC537-13	TLMF Lep 12519	KM573346	BOLD:AAF2616	Tiroler Landesmuseum
Mompha locupletella	LEALT257-16	TLMF Lep 20480	MG522471	BOLD:AAF2616	Tiroler Landesmuseum
Mompha locupletella	PHLAG241-12	TLMF Lep 06551	MG522010	BOLD:AAF2616	Research Collection of Peter Buchner
Mompha locupletella	LEFIK381-10	MM17956	KM573421	BOLD:AAF2616	University of Oulu
Mompha locupletella	LEATG585-14	TLMF Lep 14372	MG522650	BOLD:AAF2616	Tiroler Landesmuseum
Mompha locupletella	LEATE698-13	TLMF Lep 12110	MG522596	BOLD:AAF2616	Tiroler Landesmuseum
Mompha locupletella	PHLAH056-12	KLM Lep 00341	KX047310	BOLD:AAF2616	Landesmuseum Kaernten

Mompha locupletella	LEALT256-16	TLMF Lep 20479	MG521948	BOLD:AAF2616	Tiroler Landesmuseum
Mompha locupletella	LEFIG415-10	MM14441	HM876091	BOLD:AAF2616	University of Oulu
Mompha locupletella	FBLMZ593-12	BC ZSM Lep 61444	KX044311	BOLD:AAF2616	Research Collection of Peter Lichtmannecker
Mompha locupletella	LEFIG416-10	MM14442	HM876092	BOLD:AAF2616	University of Oulu
Monopis spilotella	LEALT065-16	TLMF Lep 20288	MG522708	BOLD:ABY7597	Tiroler Landesmuseum
Monopis spilotella	LEALT063-16	TLMF Lep 20286	MG522221	BOLD:ABY7597	Tiroler Landesmuseum
Monopis spilotella	LEALT064-16	TLMF Lep 20287	MG522763	BOLD:ABY7597	Tiroler Landesmuseum
Monopis spilotella	LEFIA803-10	MM04157	HM386944	BOLD:ABY7597	University of Oulu
Monopis spilotella	LEFIB595-10	MM02304	HM871474	BOLD:ABY7597	University of Oulu
Monopis spilotella	LEFIB970-10	MM03158	HM871844	BOLD:ABY7597	University of Oulu
Noctua interposita	LEATB583-13	TLMF Lep 10760	MG522747	BOLD:AAD4803	Tiroler Landesmuseum
Noctua interposita	PHLSA457-11	TLMF Lep 05912	MG522787	BOLD:AAD4803	Tiroler Landesmuseum
Noctua interposita	LEALT536-16	TLMF Lep 20759	MG521941	BOLD:AAD4803	Tiroler Landesmuseum
Noctua interposita	LEFIG405-10	MM14430	HM876081	BOLD:AAD4803	University of Oulu
Noctua interposita	FBLMV006-09	BC ZSM Lep 27986	HQ955350	BOLD:AAD4803	SNSB, Zoologische Staatssammlung Muenchen
Noctua interposita	GBLAA2812-15	BC ZSM Lep 92023	MG522286	BOLD:AAD4803	SNSB, Zoologische Staatssammlung Muenchen
Noctua interposita	FBLMV007-09	BC ZSM Lep 27987	HQ955351	BOLD:AAD4803	SNSB, Zoologische Staatssammlung Muenchen
Noctua interposita	GBLAB598-13	BC ZSM Lep 75369	MG521934	BOLD:AAD4803	SNSB, Zoologische Staatssammlung Muenchen
Noctua interposita	ABOLA517-14	TLMF Lep 16204	MG522663	BOLD:AAD4803	Tiroler Landesmuseum
Noctua interposita	LEATB582-13	TLMF Lep 10759	MG522671	BOLD:AAD4803	Naturmuseum Suedtirol
Noctua interposita	LEFID158-10	MM05922	HM872965	BOLD:AAD4803	University of Oulu
Noctua interposita	LEALT534-16	TLMF Lep 20757	MG522020	BOLD:AAD4803	Tiroler Landesmuseum
Noctua interposita	GBLAA2811-15	BC ZSM Lep 92022	MG522773	BOLD:AAD4803	SNSB, Zoologische Staatssammlung Muenchen
Noctua interposita	LEALT535-16	TLMF Lep 20758	MG522055	BOLD:AAD4803	Tiroler Landesmuseum
Noctua interposita	GBLAA1789-15	BC ZSM Lep 79790	MG522745	BOLD:AAD4803	SNSB, Zoologische Staatssammlung Muenchen
Noctua interposita	PHLAF328-11	TLMF Lep 05498	MG522107	BOLD:AAD4803	Tiroler Landesmuseum
Noctua interposita	FBLMT861-09	BC ZSM Lep 25421	GU654991	BOLD:AAD4803	SNSB, Zoologische Staatssammlung Muenchen
Noctua interposita	LEFIF303-10	MM11090	HM874990	BOLD:AAD4803	University of Oulu
Noctua interposita	ABOLA516-14	TLMF Lep 16203	MG522525	BOLD:AAD4803	Tiroler Landesmuseum
Noctua interposita	GWOTG533-12	BC ZSM Lep 63189	KX046576	BOLD:AAD4803	SNSB, Zoologische Staatssammlung Muenchen

Noctua interposita	GBLAF438-14	BC ZSM Lep 82144	MG522255	BOLD:AAD4803	SNSB, Zoologische Staatssammlung Muenchen
Noctua interposita	PHLAA286-09	TLMF Lep 00326	HM425819	BOLD:AAD4803	Tiroler Landesmuseum
Noctua interposita	LEATD385-13	TLMF Lep 13032	MG522263	BOLD:AAD4803	Naturmuseum Suedtirol
Noctua interposita	GWOTG534-12	BC ZSM Lep 63190	KX046538	BOLD:AAD4803	SNSB, Zoologische Staatssammlung Muenchen
Ochsenheimeria urella	LTOLB133-08	MM-08-0008	KF491941	BOLD:AAE5995	University of Maryland
Ochsenheimeria urella	PHLAI177-12	KLM Lep 00652	MG521970	BOLD:AAE5996	Landesmuseum Kaernten
Ochsenheimeria urella	LEFID097-10	MM05711	HM872908	BOLD:AAE5996	University of Oulu
Ochsenheimeria urella	LEALT236-16	TLMF Lep 20459	MG522367	BOLD:AAE5995	Tiroler Landesmuseum
Ochsenheimeria urella	LEALT191-16	TLMF Lep 20414	MG522404	BOLD:AAE5995	Tiroler Landesmuseum
Ochsenheimeria urella	LEFIE952-10	MM10349	HM874669	BOLD:AAE5996	University of Oulu
Ochsenheimeria urella	LEALT193-16	TLMF Lep 20416	MG522665	BOLD:AAE5995	Tiroler Landesmuseum
Ochsenheimeria urella	FBLMZ094-12	BC ZSM Lep 61420	KX045581	BOLD:AAE5996	Research Collection of Richard Heindel
Ochsenheimeria urella	PHLAI176-12	KLM Lep 00651	MG522469	BOLD:AAE5996	Landesmuseum Kaernten
Ochsenheimeria urella	LEALT192-16	TLMF Lep 20415	MG522014	BOLD:AAE5995	Tiroler Landesmuseum
Ochsenheimeria urella	LEALT237-16	TLMF Lep 20460	MG522243	BOLD:AAE5995	Tiroler Landesmuseum
Ochsenheimeria urella	LEFIJ2391-14	MM23774	KT782455	BOLD:AAE5996	University of Oulu
Ochsenheimeria urella	LEFIG537-10	MM14672	HM876210	BOLD:AAE5996	University of Oulu
Ochsenheimeria urella	LEFIJ1511-12	MM22779	MG522042	BOLD:AAE5995	University of Oulu
Oidaematophorus rogenhoferi	LEATE219-13	TLMF Lep 11631	MG522113	BOLD:AAE5830	Tiroler Landesmuseum
Oidaematophorus rogenhoferi	LEALT008-16	TLMF Lep 20231	MG522659	BOLD:AAE5830	Tiroler Landesmuseum
Oidaematophorus rogenhoferi	PHLAC711-10	TLMF Lep 02746	JF860265	BOLD:AAE5830	Tiroler Landesmuseum
Oidaematophorus rogenhoferi	LEATJ577-15	TLMF Lep 18152	MG522807	BOLD:AAE5830	Tiroler Landesmuseum
Oidaematophorus rogenhoferi	LEALT095-16	TLMF Lep 20318	MG522034	BOLD:AAE5830	Tiroler Landesmuseum
Oidaematophorus rogenhoferi	LEFID580-10	MM06583	HM873345	BOLD:AAE5830	University of Oulu
Oidaematophorus rogenhoferi	PHLAA538-09	TLMF Lep 00578	HM381420	BOLD:AAE5830	Tiroler Landesmuseum
Oidaematophorus rogenhoferi	LEALT007-16	TLMF Lep 20230	MG522712	BOLD:AAE5830	Tiroler Landesmuseum
Oidaematophorus rogenhoferi	LEATE204-13	TLMF Lep 11616	MG521978	BOLD:AAE5830	Tiroler Landesmuseum
Oidaematophorus rogenhoferi	LEATE364-13	TLMF Lep 11776	MG522213	BOLD:AAE5830	Tiroler Landesmuseum
Oidaematophorus rogenhoferi	PHLAB916-10	TLMF Lep 01716	HQ968920	BOLD:AAE5830	Tiroler Landesmuseum
Oidaematophorus rogenhoferi	PHLAB915-10	TLMF Lep 01715	HQ968919	BOLD:AAE5830	Tiroler Landesmuseum

Oidaematophorus rogenhoferi	LEFID579-10	MM06582	HM873344	BOLD:AAE5830	University of Oulu
Oidaematophorus rogenhoferi	LEFID454-10	MM06393	HM873225	BOLD:AAE5830	University of Oulu
Oidaematophorus rogenhoferi	PHLAH943-12	TLMF Lep 08747	KM573073	BOLD:AAE5830	Tiroler Landesmuseum
Papestra biren	GWORL329-09	BC ZSM Lep 22041	GU686940	BOLD:AAA9849	SNSB, Zoologische Staatssammlung Muenchen
Papestra biren	FBLMV063-09	BC ZSM Lep 28043	GU707448	BOLD:AAA9849	SNSB, Zoologische Staatssammlung Muenchen
Papestra biren	GWORO835-09	BC ZSM Lep 30527	GU688487	BOLD:AAA9849	SNSB, Zoologische Staatssammlung Muenchen
Papestra biren	GWORK556-09	BC ZSM Lep 21886	JF415640	BOLD:AAA9849	SNSB, Zoologische Staatssammlung Muenchen
Papestra biren	LEATD036-13	TLMF Lep 12683	MG522489	BOLD:AAA9849	Tiroler Landesmuseum
Papestra biren	LEATD035-13	TLMF Lep 12682	MG522389	BOLD:AAA9849	Tiroler Landesmuseum
Papestra biren	GBLAF549-14	BC ZSM Lep 82255	MG522385	BOLD:AAA9849	SNSB, Zoologische Staatssammlung Muenchen
Papestra biren	LEATD362-13	TLMF Lep 13009	MG522740	BOLD:AAA9849	Tiroler Landesmuseum
Papestra biren	LEFIF629-10	MM12519	HM875313	BOLD:AAA9849	University of Oulu
Papestra biren	LEFIA747-10	MM04101	HM386891	BOLD:AAA9849	University of Oulu
Papestra biren	PHLAE337-11	TLMF Lep 04652	JN272312	BOLD:AAA9849	Tiroler Landesmuseum
Papestra biren	LEALT462-16	TLMF Lep 20685	MG522219	BOLD:AAA9849	Tiroler Landesmuseum
Papestra biren	LEFIG616-10	MM14830	HM876282	BOLD:AAA9849	University of Oulu
Papestra biren	LEALT460-16	TLMF Lep 20683	MG522215	BOLD:AAA9849	Tiroler Landesmuseum
Papestra biren	ABOLB528-15	KLM Lep 02713	MG521957	BOLD:AAA9849	Landesmuseum Kaernten
Parnassius phoebus	PHLAA421-09	TLMF Lep 00461	HM425937	BOLD:AAB0370	Tiroler Landesmuseum
Parnassius phoebus	PHLAI537-13	TLMF Lep 09099	KP253364	BOLD:AAB0370	Tiroler Landesmuseum
Parnassius phoebus	LOWA302-06	2005-LOWA-302	FJ663904	BOLD:AAB0370	McGuire Centre for Lepidoptera and Biodiversity
Parnassius phoebus	LEALT457-16	TLMF Lep 20680	MG522531	BOLD:AAB0370	Tiroler Landesmuseum
Parnassius phoebus	PHLAC932-10	TLMF Lep 02967	JF860400	BOLD:AAB0370	Tiroler Landesmuseum
Parnassius phoebus	LEATI044-15	TLMF Lep 17429	MG522349	BOLD:AAB0370	Tiroler Landesmuseum
Parnassius phoebus	LEALT487-16	TLMF Lep 20710	MG522238	BOLD:AAB0370	Tiroler Landesmuseum
Parnassius phoebus	LEALT458-16	TLMF Lep 20681	MG522312	BOLD:AAB0370	Tiroler Landesmuseum
Parnassius phoebus	PHLAC933-10	TLMF Lep 02968	JF860401	BOLD:AAB0370	Tiroler Landesmuseum
Parnassius phoebus	PHLAC726-10	TLMF Lep 02761	JF860278	BOLD:AAB0370	Tiroler Landesmuseum
Parnassius phoebus	LOWA073-06	2005-LOWA-73	FJ663901	BOLD:AAB0370	McGuire Centre for Lepidoptera and Biodiversity
Parnassius phoebus	LOWA303-06	2005-LOWA-303	FJ663903	BOLD:AAB0370	McGuire Centre for Lepidoptera and Biodiversity

Parnassius phoebus	LEATG384-14	TLMF Lep 14171	MG522819	BOLD:AAB0370	Naturmuseum Suedtirol
Parnassius phoebus	LEATG383-14	TLMF Lep 14170	MG522306	BOLD:AAB0370	Naturmuseum Suedtirol
Parnassius phoebus	LOWA841-06	2005-LOWA-841	FJ663900	BOLD:AAB0370	McGuire Centre for Lepidoptera and Biodiversity
Parnassius phoebus	LEATI004-15	TLMF Lep 17389	MG522788	BOLD:AAB0370	Research Collection of Kurt Lechner
Parnassius phoebus	LOWA072-06	2005-LOWA-72	FJ663902	BOLD:AAB0370	McGuire Centre for Lepidoptera and Biodiversity
Pediasia aridella	LEFIE313-10	MM08672	HM874039	BOLD:AAA2313	University of Oulu
Pediasia aridella	PHLAH174-12	TLMF Lep 06674	MG522233	BOLD:AAA2313	Research Collection of Peter Buchner
Pediasia aridella	LEFIE917-10	MM10281	HM874634	BOLD:AAA2313	University of Oulu
Pediasia aridella	PHLAI045-12	KLM Lep 00520	MG521918	BOLD:AAA2313	Landesmuseum Kaernten
Pediasia aridella	PHLAE590-11	TLMF Lep 05095	KX042035	BOLD:AAA2313	Tiroler Landesmuseum
Pediasia aridella	LEFID562-10	MM06555	HM873327	BOLD:AAA2313	University of Oulu
Pediasia aridella	LEALT094-16	TLMF Lep 20317	MG522011	BOLD:AAA2313	Tiroler Landesmuseum
Pediasia aridella	LEALT093-16	TLMF Lep 20316	MG522445	BOLD:AAA2313	Tiroler Landesmuseum
Pediasia aridella	DEEUR371-12	TLMF Lep 07050	MG522311	BOLD:AAA2313	Research Collection of Peter Buchner
Perizoma hydrata	LEFIF731-10	MM12844	HM875415	BOLD:AAC0743	University of Oulu
Perizoma hydrata	LEALT451-16	TLMF Lep 20674	MG522797	BOLD:AAC0743	Tiroler Landesmuseum
Perizoma hydrata	PHLAC402-10	TLMF Lep 02437	JF859978	BOLD:AAC0743	Tiroler Landesmuseum
Perizoma hydrata	LEFIC698-10	MM04686	HM872519	BOLD:AAC0743	University of Oulu
Perizoma hydrata	LEATB405-13	TLMF Lep 10582	MG522760	BOLD:AAC0743	Tiroler Landesmuseum
Perizoma hydrata	LEFIK892-10	MM18467	JF854343	BOLD:AAC0743	University of Oulu
Perizoma hydrata	LEATB652-13	TLMF Lep 10829	MG522804	BOLD:AAC0743	Naturmuseum Suedtirol
Perizoma hydrata	LEATB480-13	TLMF Lep 10657	MG522449	BOLD:AAC0743	Tiroler Landesmuseum
Perizoma hydrata	PHLAH677-12	TLMF Lep 08496	KM572395	BOLD:AAC0743	inatura, Dornbirn
Perizoma hydrata	LEFIK893-10	MM18468	JF854344	BOLD:AAC0743	University of Oulu
Perizoma hydrata	LEALT452-16	TLMF Lep 20675	MG522670	BOLD:AAC0743	Tiroler Landesmuseum
Perizoma hydrata	LEALT450-16	TLMF Lep 20673	MG521915	BOLD:AAC0743	Tiroler Landesmuseum
Perizoma hydrata	LEATB653-13	TLMF Lep 10830	MG522661	BOLD:AAC0743	Tiroler Landesmuseum
Perizoma hydrata	PHLAF278-11	TLMF Lep 05448	MG522223	BOLD:AAC0743	Tiroler Landesmuseum
Perizoma hydrata	ABOLB663-15	KLM Lep 02848	MG522485	BOLD:AAC0743	Landesmuseum Kaernten
Perizoma hydrata	PHLAA668-09	TLMF Lep 00708	HM426069	BOLD:AAC0743	Tiroler Landesmuseum

Perizoma hydrata	PHLAF411-11	TLMF Lep 05581	MG522037	BOLD:AAC0743	Tiroler Landesmuseum
Perizoma hydrata	LEFIF387-10	MM11666	HM875072	BOLD:AAC0743	University of Oulu
Perizoma hydrata	GWOSD792-10	BC ZSM Lep 42833	JF849006	BOLD:AAC0743	SNSB, Zoologische Staatssammlung Muenchen
Phiaris obsoletana	LEALT226-16	TLMF Lep 20449	MG522414	BOLD:AAB1043	Tiroler Landesmuseum
Phiaris obsoletana	LEALT229-16	TLMF Lep 20452	MG522036	BOLD:AAB1043	Tiroler Landesmuseum
Phiaris obsoletana	LEALT037-16	TLMF Lep 20260	MG522166	BOLD:AAB1043	Tiroler Landesmuseum
Phiaris obsoletana	LEFIG234-10	MM14145	HM875913	BOLD:AAB1043	University of Oulu
Phiaris obsoletana	LEFIA762-10	MM04116	HM386905	BOLD:AAB1043	University of Oulu
Phiaris obsoletana	LEFIA763-10	MM04117	HM386906	BOLD:AAB1043	University of Oulu
Phiaris obsoletana	LEFIG820-10	MM15684	HQ570411	BOLD:AAB1043	University of Oulu
Plebejus orbitulus	LEALT385-16	TLMF Lep 20608	MG522354	BOLD:AAE5039	Tiroler Landesmuseum
Plebejus orbitulus	LEALT519-16	TLMF Lep 20742	MG522466	BOLD:AAE5039	Tiroler Landesmuseum
Plebejus orbitulus	LEALT518-16	TLMF Lep 20741	MG522722	BOLD:AAE5039	Tiroler Landesmuseum
Polia bombycina	PHLAI664-13	TLMF Lep 09227	KM573342	BOLD:AAD1839	Tiroler Landesmuseum
Polia bombycina	ABOLB500-15	KLM Lep 02685	MG522279	BOLD:AAD1839	Landesmuseum Kaernten
Polia bombycina	GBLAF433-14	BC ZSM Lep 82139	MG522674	BOLD:AAD1839	SNSB, Zoologische Staatssammlung Muenchen
Polia bombycina	FBLMT856-09	BC ZSM Lep 25416	GU654989	BOLD:AAD1839	SNSB, Zoologische Staatssammlung Muenchen
Polia bombycina	LEALT514-16	TLMF Lep 20737	MG522526	BOLD:AAD1839	Tiroler Landesmuseum
Polia bombycina	FBLMV043-09	BC ZSM Lep 28023	GU707441	BOLD:AAD1839	SNSB, Zoologische Staatssammlung Muenchen
Polia bombycina	ABOLB318-15	TLMF Lep 17323	MG522332	BOLD:AAD1839	Tiroler Landesmuseum
Polia bombycina	LEATB571-13	TLMF Lep 10748	MG522019	BOLD:AAD1839	Naturmuseum Suedtirol
Polia bombycina	LEFIF638-10	MM12540	HM875322	BOLD:AAD1839	University of Oulu
Polia bombycina	ABOLA448-14	TLMF Lep 16135	MG522116	BOLD:AAD1839	Tiroler Landesmuseum
Polia bombycina	FGMLB656-13	BC ZSM Lep 72292	MG522129	BOLD:AAD1839	SNSB, Zoologische Staatssammlung Muenchen
Polia bombycina	ABOLA551-14	TLMF Lep 16238	MG522419	BOLD:AAD1839	Tiroler Landesmuseum
Polia bombycina	ABOLA469-14	TLMF Lep 16156	MG522553	BOLD:AAD1839	Tiroler Landesmuseum
Polia bombycina	LEFIF639-10	MM12542	HM875323	BOLD:AAD1839	University of Oulu
Polia bombycina	LEFIA635-10	MM01750	HM870884	BOLD:AAD1839	University of Oulu
Polia bombycina	LEATB572-13	TLMF Lep 10749	MG521986	BOLD:AAD1839	Tiroler Landesmuseum
Polia bombycina	LEALT513-16	TLMF Lep 20736	MG522615	BOLD:AAD1839	Tiroler Landesmuseum

Polia bombycina	LEFIC864-10	MM05120	HM872682	BOLD:AAD1839	University of Oulu
Polypogon tentacularia	ABOLA550-14	TLMF Lep 16237	MG522222	BOLD:AAF0435	Tiroler Landesmuseum
Polypogon tentacularia	LEFIA364-10	MM01420	HM386706	BOLD:AAF0435	University of Oulu
Polypogon tentacularia	LEALT555-16	TLMF Lep 20778	MG522337	BOLD:AAF0435	Tiroler Landesmuseum
Polypogon tentacularia	LEATJ233-15	TLMF Lep 18663	MG522369	BOLD:AAF0435	Tiroler Landesmuseum
Polypogon tentacularia	GBLAB692-13	BC ZSM Lep 75463	MG522191	BOLD:AAF0435	SNSB, Zoologische Staatssammlung Muenchen
Polypogon tentacularia	LASTS330-14	TLMF Lep 14782	KP253313	BOLD:AAF0435	Tiroler Landesmuseum
Polypogon tentacularia	ABOLA172-14	KLM Lep 01692	MG522247	BOLD:AAF0435	Landesmuseum Kaernten
Polypogon tentacularia	LEALT553-16	TLMF Lep 20776	MG521929	BOLD:AAF0435	Tiroler Landesmuseum
Polypogon tentacularia	FBLMV264-09	BC ZSM Lep 28244	JF415835	BOLD:AAF0435	SNSB, Zoologische Staatssammlung Muenchen
Polypogon tentacularia	LEFIB863-10	MM02906	HM871740	BOLD:AAF0435	University of Oulu
Polypogon tentacularia	LEATJ1101-15	TLMF Lep 18961	MG522060	BOLD:AAF0435	Tiroler Landesmuseum
Polypogon tentacularia	ABOLB814-15	KLM Lep 03474	MG521920	BOLD:AAF0435	Landesmuseum Kaernten
Polypogon tentacularia	LEFIC637-10	MM04580	HM872458	BOLD:AAF0435	University of Oulu
Polypogon tentacularia	GBLGC342-12	BC ZSM Lep R 21672	MG522194	BOLD:AAF0435	SNSB, Zoologische Staatssammlung Muenchen
Polypogon tentacularia	LEALT554-16	TLMF Lep 20777	MG522791	BOLD:AAF0435	Tiroler Landesmuseum
Polypogon tentacularia	ABOLA482-14	TLMF Lep 16169	MG522052	BOLD:AAF0435	Tiroler Landesmuseum
Polypogon tentacularia	FBLMW101-10	BC ZSM Lep 29031	HQ563387	BOLD:AAF0435	Research Collection of Alfred Haslberger
Pontia callidice	LEATG067-14	TLMF Lep 13854	MG522242	BOLD:AAC9448	Tiroler Landesmuseum
Pontia callidice	LEATC114-13	TLMF Lep 11241	MG522398	BOLD:AAC9448	Naturmuseum Suedtirol
Pontia callidice	LEATC618-13	TLMF Lep 12600	KP253739	BOLD:AAC9448	Tiroler Landesmuseum
Pontia callidice	LEATC115-13	TLMF Lep 11242	MG522798	BOLD:AAC9448	Tiroler Landesmuseum
Pontia callidice	LEALT561-16	TLMF Lep 20784	MG522137	BOLD:ACE3801	Tiroler Landesmuseum
Pontia callidice	PHLAH290-12	TLMF Lep 07729	MG522785	BOLD:AAC9448	Tiroler Landesmuseum
Pontia callidice	LEATG066-14	TLMF Lep 13853	MG522204	BOLD:AAC9448	Tiroler Landesmuseum
Pontia callidice	LOWA629-06	2005-LOWA-629	FJ664003	BOLD:ACE3801	McGuire Centre for Lepidoptera and Biodiversity
Pontia callidice	LOWA520-06	2005-LOWA-520	FJ664004	BOLD:ACE3801	McGuire Centre for Lepidoptera and Biodiversity
Protolampra sobrina	LEALT434-16	TLMF Lep 20657	MG522234	BOLD:AAE8181	Tiroler Landesmuseum
Protolampra sobrina	LEFIB808-10	MM02737	HM871685	BOLD:AAE8181	University of Oulu

Protolampra sobrina	LEALT433-16	TLMF Lep 20656	MG522475	BOLD:AAE8181	Tiroler Landesmuseum
Protolampra sobrina	LEFIA607-10	MM01719	HM870856	BOLD:AAE8181	University of Oulu
Protolampra sobrina	PHLAI274-13	TLMF Lep 08769	KM573450	BOLD:AAE8181	Tiroler Landesmuseum
Protolampra sobrina	FBLMV020-09	BC ZSM Lep 28000	HQ955360	BOLD:AAE8181	SNSB, Zoologische Staatssammlung Muenchen
Protolampra sobrina	LEALT435-16	TLMF Lep 20658	MG522160	BOLD:AAE8181	Tiroler Landesmuseum
Protolampra sobrina	LEFIA606-10	MM01718	HM870855	BOLD:AAE8181	University of Oulu
Protolampra sobrina	FBLMV021-09	BC ZSM Lep 28001	HM422193	BOLD:AAE8181	SNSB, Zoologische Staatssammlung Muenchen
Protolampra sobrina	ABOLB317-15	TLMF Lep 17322	MG522371	BOLD:AAE8181	Tiroler Landesmuseum
Pyrausta aerealis	PHLAC299-10	TLMF Lep 02334	JF859898	BOLD:AAC7368	Tiroler Landesmuseum
Pyrausta aerealis	LEASS146-16	TLMF Lep 19989	MG522677	BOLD:AAC7368	Tiroler Landesmuseum
Pyrausta aerealis	LASTS073-14	TLMF Lep 14525	MG522296	BOLD:AAC7368	Tiroler Landesmuseum
Pyrausta aerealis	PHLAB112-10	TLMF Lep 00912	HM381484	BOLD:AAC7368	Tiroler Landesmuseum
Pyrausta aerealis	FBLMZ453-12	BC ZSM Lep 61209	KX046782	BOLD:AAC7368	Research Collection of Peter Lichtmannecker
Pyrausta aerealis	PHLAA477-09	TLMF Lep 00517	HM381363	BOLD:AAC7368	Tiroler Landesmuseum
Pyrausta aerealis	LEATB943-13	TLMF Lep 11120	MG522149	BOLD:AAC7368	Tiroler Landesmuseum
Pyrausta aerealis	PHLAF073-11	TLMF Lep 05243	MG522054	BOLD:AAC7368	Tiroler Landesmuseum
Pyrausta aerealis	LEATE775-13	TLMF Lep 12187	MG522521	BOLD:AAC7368	Tiroler Landesmuseum
Pyrausta aerealis	LEALT198-16	TLMF Lep 20421	MG522344	BOLD:AAC7368	Tiroler Landesmuseum
Pyrausta aerealis	LEALT166-16	TLMF Lep 20389	MG521977	BOLD:AAC7368	Tiroler Landesmuseum
Pyrausta aerealis	LEFIK264-10	MM17839	KM572264	BOLD:AAF8527	University of Oulu
Pyrausta aerealis	PHLAA594-09	TLMF Lep 00634	HM426002	BOLD:AAC7368	Tiroler Landesmuseum
Pyrausta aerealis	LEATB682-13	TLMF Lep 10859	MG522755	BOLD:AAC7368	Naturmuseum Suedtirol
Pyrausta aerealis	LEALT165-16	TLMF Lep 20388	MG522416	BOLD:AAC7368	Tiroler Landesmuseum
Scopula incanata	GBLAA1434-15	BC ZSM Lep 87225	MG522544	BOLD:AAC9956	SNSB, Zoologische Staatssammlung Muenchen
Scopula incanata	LEATB397-13	TLMF Lep 10574	MG522008	BOLD:AAC9956	Tiroler Landesmuseum
Scopula incanata	LEATB825-13	TLMF Lep 11002	MG522810	BOLD:AAC9956	Tiroler Landesmuseum
Scopula incanata	PHLAC464-10	TLMF Lep 02499	JF860039	BOLD:AAC9956	Tiroler Landesmuseum
Scopula incanata	PHLAF251-11	TLMF Lep 05421	KF807155	BOLD:ACF2292	Tiroler Landesmuseum
Scopula incanata	GBLAA1303-15	BC ZSM Lep 87189	MG522447	BOLD:AAC9956	SNSB, Zoologische Staatssammlung Muenchen
Scopula incanata	PHLSA455-11	TLMF Lep 05910	KF807582	BOLD:ACF0740	Tiroler Landesmuseum

Scopula incanata	PHLAH809-12	TLMF Lep 08613	KF807586	BOLD:ACF0740	Tiroler Landesmuseum
Scopula incanata	PHLAA223-09	TLMF Lep 00263	HM381352	BOLD:AAC9956	Tiroler Landesmuseum
Scopula incanata	LEFIF733-10	MM12852	HM875417	BOLD:AAC9956	University of Oulu
Scopula incanata	PHLAB1163-10	TLMF Lep 01963	HQ968375	BOLD:ACF0740	Tiroler Landesmuseum
Scopula incanata	PHLAF313-11	TLMF Lep 05483	KF807961	BOLD:AAC9956	Tiroler Landesmuseum
Scopula incanata	LEFIK838-10	MM18413	JN285636	BOLD:AAC9956	University of Oulu
Scopula incanata	ABOLB592-15	KLM Lep 02777	MG522239	BOLD:AAC9956	Landesmuseum Kaernten
Scopula incanata	PHLAH679-12	TLMF Lep 08498	KF807436	BOLD:AAC9956	inatura, Dornbirn
Scopula incanata	LEALT522-16	TLMF Lep 20745	MG522823	BOLD:AAC9956	Tiroler Landesmuseum
Scopula incanata	LEATB646-13	TLMF Lep 10823	MG522429	BOLD:AAC9956	Naturmuseum Suedtirol
Scopula incanata	LEALT520-16	TLMF Lep 20743	MG522706	BOLD:AAC9956	Tiroler Landesmuseum
Scopula incanata	LEFIC624-10	MM04558	HM872445	BOLD:AAC9956	University of Oulu
Scopula incanata	PHLAA318-09	TLMF Lep 00358	HQ968172	BOLD:ACF0740	Tiroler Landesmuseum
Scopula incanata	LEATD492-13	TLMF Lep 13139	MG522401	BOLD:AAC9956	Tiroler Landesmuseum
Scopula incanata	LEALT521-16	TLMF Lep 20744	MG521956	BOLD:AAC9956	Tiroler Landesmuseum
Scopula virgulata	LEALT472-16	TLMF Lep 20695	MG522209	BOLD:AAP7443	Tiroler Landesmuseum
Scopula virgulata	LEALT470-16	TLMF Lep 20693	MG521951	BOLD:AAP7443	Tiroler Landesmuseum
Scopula virgulata	LEALT471-16	TLMF Lep 20694	MG522322	BOLD:AAP7443	Tiroler Landesmuseum
Scopula virgulata	LEFIJ2041-13	MM22893	KM572020	BOLD:AAP7443	University of Oulu
Scopula virgulata	LEFIL398-10	MM18696	JF854583	BOLD:AAP7443	University of Oulu
Scopula virgulata	GBLAB1741-14	BC ZSM Lep 76986	MG522750	BOLD:AAP7443	Zentrum fur Biodokumentation des Saarlandes
Scopula virgulata	ABOLB116-15	TLMF Lep 17121	MG522733	BOLD:AAP7443	Tiroler Landesmuseum
Scopula virgulata	LEFIL397-10	MM18695	JF854582	BOLD:AAP7443	University of Oulu
Scopula virgulata	ABOLB589-15	KLM Lep 02774	MG522146	BOLD:AAP7443	Landesmuseum Kaernten
Scopula virgulata	LEFIJ2237-14	MM23417	KT782423	BOLD:AAP7443	University of Oulu
Scopula virgulata	PHLAV309-12	TLMF Lep 08128	KF807655	BOLD:AAP7443	inatura, Dornbirn
Scopula virgulata	LEFIJ805-10	MM17430	KM573709	BOLD:AAP7443	University of Oulu
Scopula virgulata	LEFIJ804-10	MM17429	JF853833	BOLD:AAP7443	University of Oulu
Scotopteryx chenopodiata	GBLAA426-14	BC ZSM Lep 80517	MG522483	BOLD:AAB5023	SNSB, Zoologische Staatssammlung Muenchen
Scotopteryx chenopodiata	GWORG048-08	BC ZSM Lep 02868	JF415275	BOLD:AAB5023	SNSB, Zoologische Staatssammlung Muenchen

Scotopteryx chenopodiata	LEALT503-16	TLMF Lep 20726	MG522320	BOLD:AAB5023	Tiroler Landesmuseum
Scotopteryx chenopodiata	LEFIE037-10	MM08120	HM873786	BOLD:AAB5023	University of Oulu
Scotopteryx chenopodiata	LEFIA352-10	MM01399	HM386695	BOLD:AAB5023	University of Oulu
Scotopteryx chenopodiata	NOLEP064-14	BCZMBNLep0064	MG522120	BOLD:AAB5023	University of Bergen, Natural History Collections
Scotopteryx chenopodiata	LEATB819-13	TLMF Lep 10996	MG522698	BOLD:AAB5023	Tiroler Landesmuseum
Scotopteryx chenopodiata	LEALT505-16	TLMF Lep 20728	MG522138	BOLD:AAB5023	Tiroler Landesmuseum
Scotopteryx chenopodiata	GBLAD785-14	BC ZSM Lep 84296	MG522140	BOLD:AAB5023	SNSB, Zoologische Staatssammlung Muenchen
Scotopteryx chenopodiata	GBLAD134-14	BC ZSM Lep 78705	MG522274	BOLD:AAB5023	SNSB, Zoologische Staatssammlung Muenchen
Scotopteryx chenopodiata	GWORB823-07	BC ZSM Lep 01199	HQ601541	BOLD:AAB5023	SNSB, Zoologische Staatssammlung Muenchen
Scotopteryx chenopodiata	GBLAA2068-15	BC ZSM Lep 100072	MG522283	BOLD:AAB5023	SNSB, Zoologische Staatssammlung Muenchen
Scotopteryx chenopodiata	GBLAA2067-15	BC ZSM Lep 100071	MG522106	BOLD:AAB5023	SNSB, Zoologische Staatssammlung Muenchen
Scotopteryx chenopodiata	GBLGC161-12	BC ZSM Lep R 21111	MG522170	BOLD:AAB5023	SNSB, Zoologische Staatssammlung Muenchen
Scotopteryx chenopodiata	LEATD581-13	TLMF Lep 13228	MG522838	BOLD:AAB5023	Tiroler Landesmuseum
Scotopteryx chenopodiata	GBLAC113-13	BC ZSM Lep 66854	MG522132	BOLD:AAB5023	SNSB, Zoologische Staatssammlung Muenchen
Scotopteryx chenopodiata	GBLAF376-14	BC ZSM Lep 82082	MG522757	BOLD:AAB5023	SNSB, Zoologische Staatssammlung Muenchen
Scotopteryx chenopodiata	LEFIF729-10	MM12825	HM875413	BOLD:AAB5023	University of Oulu
Scotopteryx chenopodiata	LEATD491-13	TLMF Lep 13138	MG522435	BOLD:AAB5023	Tiroler Landesmuseum
Scotopteryx chenopodiata	GWOR3827-09	BC ZSM Lep 21111	HQ601540	BOLD:AAB5023	SNSB, Zoologische Staatssammlung Muenchen
Scotopteryx chenopodiata	LEALT504-16	TLMF Lep 20727	MG522658	BOLD:AAB5023	Tiroler Landesmuseum
Scotopteryx chenopodiata	LEATB692-13	TLMF Lep 10869	MG522109	BOLD:AAB5023	Naturmuseum Suedtirol
Scotopteryx chenopodiata	GBLAC111-13	BC ZSM Lep 66852	MG522184	BOLD:AAB5023	SNSB, Zoologische Staatssammlung Muenchen
Scotopteryx chenopodiata	ABOLB833-15	KLM Lep 03493	MG522236	BOLD:AAB5023	Landesmuseum Kaernten
Scotopteryx chenopodiata	PHLAH675-12	TLMF Lep 08494	KM572542	BOLD:AAB5023	inatura, Dornbirn
Scotopteryx chenopodiata	PHLAF314-11	TLMF Lep 05484	MG522566	BOLD:AAB5023	Tiroler Landesmuseum
Scotopteryx chenopodiata	GWORM058-09	BC ZSM Lep 24107	GU687286	BOLD:AAB5023	Research Collection of Alfred Haslberger
Scotopteryx chenopodiata	LEFIJ1318-11	MM21178	KM572556	BOLD:AAB5023	University of Oulu
Scrobipalpula diffluella	LEFIJ957-11	MM21197	KM373591	BOLD:AAF1106	University of Oulu

Scrobipalpula diffluella	LEALT138-16	TLMF Lep 20361	MG522641	BOLD:AAF1106	Tiroler Landesmuseum
Scrobipalpula diffluella	PHLAI938-13	KLM Lep 01128	MG522668	BOLD:AAF1106	Landesmuseum Kaernten
Scrobipalpula diffluella	PHLAC719-10	TLMF Lep 02754	JF860272	BOLD:AAF1106	Tiroler Landesmuseum
Scrobipalpula diffluella	LEALT180-16	TLMF Lep 20403	MG522513	BOLD:AAF1106	Tiroler Landesmuseum
Scrobipalpula diffluella	LEALT153-16	TLMF Lep 20376	MG522148	BOLD:AAF1106	Tiroler Landesmuseum
Scrobipalpula diffluella	LEALT137-16	TLMF Lep 20360	MG522761	BOLD:AAF1106	Tiroler Landesmuseum
Scrobipalpula diffluella	LEALT122-16	TLMF Lep 20345	MG522141	BOLD:AAF1106	Tiroler Landesmuseum
Scrobipalpula diffluella	LEATI138-15	TLMF Lep 17523	MG522681	BOLD:AAF1106	Tiroler Landesmuseum
Scrobipalpula diffluella	PHLAB258-10	TLMF Lep 01058	HM381618	BOLD:AAF1106	Tiroler Landesmuseum
Scrobipalpula diffluella	PHLAB259-10	TLMF Lep 01059	HM381619	BOLD:AAF1106	Tiroler Landesmuseum
Scrobipalpula diffluella	PHLAA737-09	TLMF Lep 00777	HM381467	BOLD:AAF1106	Tiroler Landesmuseum
Scrobipalpula diffluella	LEALT214-16	TLMF Lep 20437	MG522039	BOLD:AAF1106	Tiroler Landesmuseum
Scrobipalpula diffluella	LEATI139-15	TLMF Lep 17524	MG522226	BOLD:AAF1106	Tiroler Landesmuseum
Selagia spadicella	LEATE446-13	TLMF Lep 11858	MG522548	BOLD:AAE1543	Tiroler Landesmuseum
Selagia spadicella	ABOLA286-14	TLMF Lep 15308	MG521996	BOLD:AAE1543	Tiroler Landesmuseum
Selagia spadicella	LEFIF781-10	MM13015	HM875465	BOLD:AAE1543	University of Oulu
Selagia spadicella	ABOLA817-15	TLMF Lep 16777	MG522197	BOLD:AAE1543	Tiroler Landesmuseum
Selagia spadicella	LEFIC178-10	MM03532	HM872023	BOLD:AAE1543	University of Oulu
Selagia spadicella	LEALT119-16	TLMF Lep 20342	MG522167	BOLD:AAE1543	Tiroler Landesmuseum
Selagia spadicella	LEALT118-16	TLMF Lep 20341	MG522330	BOLD:AAE1543	Tiroler Landesmuseum
Selagia spadicella	ABOLA287-14	TLMF Lep 15309	MG522086	BOLD:AAE1543	Tiroler Landesmuseum
Selagia spadicella	LEFIC088-10	MM03388	HM871956	BOLD:AAE1543	University of Oulu
Selagia spadicella	LEALT087-16	TLMF Lep 20310	MG522421	BOLD:AAE1543	Tiroler Landesmuseum
Setina irrorella	LEALT391-16	TLMF Lep 20614	MG522742	BOLD:ADE0333	Tiroler Landesmuseum
Setina irrorella	LEALT389-16	TLMF Lep 20612	MG522162	BOLD:ADE0333	Tiroler Landesmuseum
Setina irrorella	LEATB559-13	TLMF Lep 10736	MG522265	BOLD:AAC0260	Naturmuseum Suedtirol
Setina irrorella	PHLAA266-09	TLMF Lep 00306	HM425803	BOLD:ACF4655	Tiroler Landesmuseum
Setina irrorella	PHLAA006-09	TLMF Lep 00046	GU689188	BOLD:AAC0260	Tiroler Landesmuseum
Setina irrorella	PHLAH802-12	TLMF Lep 08606	MG522501	BOLD:ABZ4610	Tiroler Landesmuseum
Setina irrorella	LEATD479-13	TLMF Lep 13126	MG522103	BOLD:ABZ5368	Tiroler Landesmuseum

Setina irrorella	LEALT292-16	TLMF Lep 20515	MG522536	BOLD:ADE0333	Tiroler Landesmuseum
Setina irrorella	PHLAH801-12	TLMF Lep 08605	MG522450	BOLD:ABZ5368	Tiroler Landesmuseum
Setina irrorella	PHLAF321-11	TLMF Lep 05491	MG522839	BOLD:ABY4807	Tiroler Landesmuseum
Setina irrorella	PHLAA294-09	TLMF Lep 00334	HQ968171	BOLD:ABZ4609	Tiroler Landesmuseum
Setina irrorella	PHLSA664-11	TLMF Lep 06119	KM572083	BOLD:ACF4655	Tiroler Landesmuseum
Setina irrorella	LEALT293-16	TLMF Lep 20516	MG522549	BOLD:ADE0333	Tiroler Landesmuseum
Setina irrorella	PHLAB1148-10	TLMF Lep 01948	HQ968360	BOLD:AAC0260	Tiroler Landesmuseum
Setina irrorella	LEFIF133-10	MM10589	HM874831	BOLD:ABZ4612	University of Oulu
Setina irrorella	PHLAA293-09	TLMF Lep 00333	HM425826	BOLD:ABZ4609	Tiroler Landesmuseum
Setina irrorella	LEALT291-16	TLMF Lep 20514	MG521933	BOLD:ADE0333	Tiroler Landesmuseum
Setina irrorella	LEFIJ572-10	MM17197	JF853685	BOLD:ABZ4612	University of Oulu
Setina irrorella	LEFID143-10	MM05904	HM872953	BOLD:ABZ4612	University of Oulu
Setina irrorella	PHLAA121-09	TLMF Lep 00161	GU689164	BOLD:ACF5607	Tiroler Landesmuseum
Setina irrorella	PHLAA036-09	TLMF Lep 00076	GU689213	BOLD:AAC0260	Tiroler Landesmuseum
Setina irrorella	PHLAH800-12	TLMF Lep 08604	MG522082	BOLD:ABZ5368	Tiroler Landesmuseum
Setina irrorella	PHLSA603-11	TLMF Lep 06058	MG522732	BOLD:AAC0260	Tiroler Landesmuseum
Sparganothis pilleriana	LEALT249-16	TLMF Lep 20472	MG522610	BOLD:AAI0179	Tiroler Landesmuseum
Sparganothis pilleriana	LEATA467-13	TLMF Lep 10074	MG522319	BOLD:AAI0179	Tiroler Landesmuseum
Sparganothis pilleriana	LEATB087-13	TLMF Lep 10264	MG522185	BOLD:AAI0179	Tiroler Landesmuseum
Sparganothis pilleriana	FBLMZ222-12	BC ZSM Lep 64303	KX045395	BOLD:AAI0179	Research Collection of Alfred Haslberger
Sparganothis pilleriana	LEATA468-13	TLMF Lep 10075	MG522626	BOLD:AAI0179	Tiroler Landesmuseum
Sparganothis pilleriana	LEALT250-16	TLMF Lep 20473	MG522539	BOLD:AAI0179	Tiroler Landesmuseum
Sparganothis pilleriana	LEALT248-16	TLMF Lep 20471	MG522290	BOLD:AAI0179	Tiroler Landesmuseum
Sparganothis pilleriana	PHLAH630-12	TLMF Lep 08449	KP253541	BOLD:AAI0179	inatura, Dornbirn
Sparganothis pilleriana	LEATB086-13	TLMF Lep 10263	MG522047	BOLD:AAI0179	Naturmuseum Suedtirol
Syngrapha ain	LEALT322-16	TLMF Lep 20545	MG522579	BOLD:ABZ6944	Tiroler Landesmuseum
Syngrapha ain	LASTS381-14	TLMF Lep 14833	KP253380	BOLD:ABZ6944	Research Collection of Toni Mayr
Syngrapha ain	PHLAA312-09	TLMF Lep 00352	HM425844	BOLD:ABZ6944	Tiroler Landesmuseum
Syngrapha ain	ABOLB399-15	KLM Lep 02584	MG522751	BOLD:ABZ6944	Landesmuseum Kaernten
Syngrapha ain	LEALT463-16	TLMF Lep 20686	MG522649	BOLD:ABZ6944	Tiroler Landesmuseum

Syngrapha ain	PHLAB300-10	TLMF Lep 01100	HQ968467	BOLD:ABZ6944	Tiroler Landesmuseum
Syngrapha ain	PHLAC431-10	TLMF Lep 02466	JF860007	BOLD:ABZ6944	Tiroler Landesmuseum
Syngrapha ain	ABOLC038-16	TLMF Lep 20071	MG522782	BOLD:ABZ6944	Tiroler Landesmuseum
Syngrapha ain	LEATC139-13	TLMF Lep 11266	MG521991	BOLD:ABZ6944	Tiroler Landesmuseum
Syngrapha ain	LEALT323-16	TLMF Lep 20546	MG522540	BOLD:ABZ6944	Tiroler Landesmuseum
Syngrapha ain	LEATB385-13	TLMF Lep 10562	MG522527	BOLD:ABZ6944	Tiroler Landesmuseum
Syngrapha ain	FBLMZ536-12	BC ZSM Lep 61292	KX046403	BOLD:ABZ6944	Research Collection of Peter Lichtmannecker
Syngrapha ain	LEATB691-13	TLMF Lep 10868	MG522687	BOLD:ABZ6944	Naturmuseum Suedtirol
Syngrapha ain	FBLMZ179-12	BC ZSM Lep 51435	KX045183	BOLD:ABZ6944	Research Collection of Alfred Haslberger
Syngrapha hochenwarthi	LEALT447-16	TLMF Lep 20670	MG522013	BOLD:ABX4966	Tiroler Landesmuseum
Syngrapha hochenwarthi	PHLAC798-10	TLMF Lep 02833	JF860315	BOLD:ABX4966	Tiroler Landesmuseum
Syngrapha hochenwarthi	PHLAA133-09	TLMF Lep 00173	GU689153	BOLD:ABX4966	Tiroler Landesmuseum
Syngrapha hochenwarthi	PHLAC797-10	TLMF Lep 02832	JF860314	BOLD:ABX4966	Tiroler Landesmuseum
Syngrapha hochenwarthi	LEFID147-10	MM05909	HQ570322	BOLD:ABX4966	University of Oulu
Syngrapha hochenwarthi	PHLAA132-09	TLMF Lep 00172	GU689159	BOLD:ABX4966	Tiroler Landesmuseum
Syngrapha hochenwarthi	LEATC106-13	TLMF Lep 11233	MG522382	BOLD:ABX4966	Naturmuseum Suedtirol
Syngrapha hochenwarthi	FBLMZ178-12	BC ZSM Lep 51434	KX040972	BOLD:ABX4966	Research Collection of Alfred Haslberger
Syngrapha hochenwarthi	LEALT448-16	TLMF Lep 20671	MG522468	BOLD:ABX4966	Tiroler Landesmuseum
Syngrapha hochenwarthi	PHLAC725-10	TLMF Lep 02760	KX042651	BOLD:ABX4966	Tiroler Landesmuseum
Syngrapha hochenwarthi	LEFIA743-10	MM04097	HM386887	BOLD:ABX4966	University of Oulu
Syngrapha hochenwarthi	LEFIA001-10	MM00072	HM396351	BOLD:ABX4966	University of Oulu
Syngrapha hochenwarthi	LEATC107-13	TLMF Lep 11234	MG522759	BOLD:ABX4966	Tiroler Landesmuseum
Syngrapha interrogationis	LEFIC763-10	MM04885	HM872582	BOLD:AAB3481	University of Oulu
Syngrapha interrogationis	LEATC141-13	TLMF Lep 11268	MG522121	BOLD:AAB3481	Tiroler Landesmuseum
Syngrapha interrogationis	PHLAB304-10	TLMF Lep 01104	HQ968471	BOLD:AAB3481	Tiroler Landesmuseum
Syngrapha interrogationis	ABOLB400-15	KLM Lep 02585	MG522688	BOLD:AAB3481	Landesmuseum Kaernten
Syngrapha interrogationis	LEALT502-16	TLMF Lep 20725	MG522007	BOLD:AAB3481	Tiroler Landesmuseum
Syngrapha interrogationis	ABOLC037-16	TLMF Lep 20070	MG522264	BOLD:AAB3481	Tiroler Landesmuseum
Syngrapha interrogationis	PHLAC430-10	TLMF Lep 02465	JF860006	BOLD:AAB3481	Tiroler Landesmuseum
Syngrapha interrogationis	LEALT500-16	TLMF Lep 20723	MG522444	BOLD:AAB3481	Tiroler Landesmuseum

Syngrapha interrogationis	PHLAB338-10	TLMF Lep 01138	HQ968500	BOLD:AAB3481	Tiroler Landesmuseum
Syngrapha interrogationis	LEFIB816-10	MM02752	HM871693	BOLD:AAB3481	University of Oulu
Syngrapha interrogationis	LEFIH144-10	MM15052	HM876764	BOLD:AAB3481	University of Oulu
Syngrapha interrogationis	GMGRE2273-13	BIOUG06173-B11	MG522592	BOLD:AAB3481	SNSB, Zoologische Staatssammlung Muenchen
Syngrapha interrogationis	LEFIH173-10	MM15145	HM876793	BOLD:AAB3481	University of Oulu
Syngrapha interrogationis	LEFID903-10	MM07253	HM873653	BOLD:AAB3481	University of Oulu
Syngrapha interrogationis	PHLAB303-10	TLMF Lep 01103	HQ968470	BOLD:AAB3481	Tiroler Landesmuseum
Syngrapha interrogationis	FBLMV211-09	BC ZSM Lep 28191	GU707312	BOLD:AAB3481	SNSB, Zoologische Staatssammlung Muenchen
Syngrapha interrogationis	LEATB549-13	TLMF Lep 10726	MG522582	BOLD:AAB3481	Naturmuseum Suedtirol
Syngrapha interrogationis	LEFIH174-10	MM15150	HM876794	BOLD:AAB3481	University of Oulu
Syngrapha interrogationis	LEATC199-13	TLMF Lep 11326	MG521980	BOLD:AAB3481	Tiroler Landesmuseum
Syngrapha interrogationis	LEFIH145-10	MM15064	HM876765	BOLD:AAB3481	University of Oulu
Syngrapha interrogationis	PHLAH680-12	TLMF Lep 08499	KM572190	BOLD:AAB3481	inatura, Dornbirn
Syngrapha interrogationis	PHLAA257-09	TLMF Lep 00297	HM425794	BOLD:AAB3481	Tiroler Landesmuseum
Syngrapha interrogationis	LEFIH175-10	MM15155	HM876795	BOLD:AAB3481	University of Oulu
Syngrapha interrogationis	LEFIH177-10	MM15164	HM876797	BOLD:AAB3481	University of Oulu
Syngrapha interrogationis	LEFIH176-10	MM15157	HM876796	BOLD:AAB3481	University of Oulu
Trichiura crataegi	LEFIC220-10	MM03616	HM872064	BOLD:AAB4489	University of Oulu
Trichiura crataegi	LEATG211-14	TLMF Lep 13998	MG522168	BOLD:AAB4489	Tiroler Landesmuseum
Trichiura crataegi	GBLAD189-14	BC ZSM Lep 78760	MG522689	BOLD:AAB4489	SNSB, Zoologische Staatssammlung Muenchen
Trichiura crataegi	LEATD124-13	TLMF Lep 12771	MG522616	BOLD:AAB4489	Tiroler Landesmuseum
Trichiura crataegi	LEFID925-10	MM07389	HM873675	BOLD:AAB4489	University of Oulu
Trichiura crataegi	FBLMU946-09	BC ZSM Lep 27976	HQ955344	BOLD:AAB4489	SNSB, Zoologische Staatssammlung Muenchen
Trichiura crataegi	LEFIB312-10	MM00836	HM871212	BOLD:AAB4489	University of Oulu
Trichiura crataegi	LEFIA943-10	MM13871	HM387076	BOLD:AAB4489	University of Oulu
Trichiura crataegi	LEFIA124-10	MM01052	HM396471	BOLD:AAB4489	University of Oulu
Trichiura crataegi	LEALT304-16	TLMF Lep 20527	MG522474	BOLD:AAB4489	Tiroler Landesmuseum
Trichiura crataegi	ABOLB538-15	KLM Lep 02723	MG522546	BOLD:AAB4489	Landesmuseum Kaernten
Trichiura crataegi	LEFIA123-10	MM01051	HM396470	BOLD:AAB4489	University of Oulu
Trichiura crataegi	PHLAA183-09	TLMF Lep 00223	GU689129	BOLD:AAB4489	Tiroler Landesmuseum

Trichiura crataegi	LEFIB835-10	MM02797	HM871712	BOLD:AAB4489	University of Oulu
Trichiura crataegi	LEFIA942-10	MM13870	HM387075	BOLD:AAB4489	University of Oulu
Trichiura crataegi	GWORK492-09	BC ZSM Lep 21822	JF415446	BOLD:AAB4489	SNSB, Zoologische Staatssammlung Muenchen
Trichiura crataegi	GBLAA1670-15	BC ZSM Lep 80051	MG521947	BOLD:AAB4489	SNSB, Zoologische Staatssammlung Muenchen
Trichiura crataegi	PHLAH672-12	TLMF Lep 08491	KM572357	BOLD:AAB4489	inatura, Dornbirn
Trichiura crataegi	LEALT388-16	TLMF Lep 20611	MG522033	BOLD:AAB4489	Tiroler Landesmuseum
Trichiura crataegi	GWORK344-09	BC ZSM Lep 21579	JF415447	BOLD:AAB4489	SNSB, Zoologische Staatssammlung Muenchen
Trichiura crataegi	GBLAA1671-15	BC ZSM Lep 80052	MG522470	BOLD:AAB4489	SNSB, Zoologische Staatssammlung Muenchen
Trichiura crataegi	GBLAB1759-14	BC ZSM Lep 77004	MG522694	BOLD:AAB4489	Zentrum fur Biodokumentation des Saarlandes
Trichiura crataegi	LEATD123-13	TLMF Lep 12770	MG521961	BOLD:AAB4489	Tiroler Landesmuseum
Trichiura crataegi	LEFIE107-10	MM08292	HM873855	BOLD:AAB4489	University of Oulu
Trichiura crataegi	LEALT305-16	TLMF Lep 20528	MG522211	BOLD:AAB4489	Tiroler Landesmuseum
Trichiura crataegi	LEATD556-13	TLMF Lep 13203	MG522249	BOLD:AAB4489	Tiroler Landesmuseum
Trichiura crataegi	LEFIF650-10	MM12574	HM875334	BOLD:AAB4489	University of Oulu
Trichiura crataegi	LEFIE032-10	MM08104	HM873781	BOLD:AAB4489	University of Oulu
Udea uliginosalis	LEALT245-16	TLMF Lep 20468	MG191931	BOLD:ADE0060	Tiroler Landesmuseum
Udea uliginosalis	PHLAB173-10	TLMF Lep 00973	HM381538	BOLD:AAC7935	Tiroler Landesmuseum
Udea uliginosalis	LEATJ678-15	TLMF Lep 18253	MG522408	BOLD:AAC7935	Tiroler Landesmuseum
Udea uliginosalis	PHLAA595-09	TLMF Lep 00635	HM426003	BOLD:ACE9766	Tiroler Landesmuseum
Udea uliginosalis	LEALT562-16	TLMF Lep 20785	MG191929	BOLD:ADE0060	Tiroler Landesmuseum
Udea uliginosalis	PHLAB096-10	TLMF Lep 00896	HQ968269	BOLD:ACE9765	Tiroler Landesmuseum
Udea uliginosalis	PHLAB023-10	TLMF Lep 00823	HQ968199	BOLD:AAC7935	Tiroler Landesmuseum
Udea uliginosalis	LEATH119-14	TLMF Lep 15236	MG521995	BOLD:AAC7935	Tiroler Landesmuseum
Udea uliginosalis	PHLAB661-10	TLMF Lep 01461	HQ968677	BOLD:AAC7935	Tiroler Landesmuseum
Udea uliginosalis	LEATE201-13	TLMF Lep 11613	MG522607	BOLD:AAC7935	Tiroler Landesmuseum
Udea uliginosalis	PHLAB904-10	TLMF Lep 01704	HQ968908	BOLD:AAC7935	Tiroler Landesmuseum
Udea uliginosalis	PHLAA537-09	TLMF Lep 00577	HM381419	BOLD:ACE9765	Tiroler Landesmuseum
Udea uliginosalis	LEATE770-13	TLMF Lep 12182	MG522691	BOLD:ACE9765	Tiroler Landesmuseum
Udea uliginosalis	PHLAB903-10	TLMF Lep 01703	HQ968907	BOLD:AAC7935	Tiroler Landesmuseum
Udea uliginosalis	PHLAB222-10	TLMF Lep 01022	HM381586	BOLD:ACE9766	Tiroler Landesmuseum

Udea uliginosalis	PHLAB024-10	TLMF Lep 00824	HQ968200	BOLD:AAC7935	Tiroler Landesmuseum
Udea uliginosalis	LEATE200-13	TLMF Lep 11612	MG522097	BOLD:AAC7935	Tiroler Landesmuseum
Udea uliginosalis	PHLAE583-11	TLMF Lep 05088	KX042480	BOLD:AAC7935	Tiroler Landesmuseum
Udea uliginosalis	LEATC376-13	TLMF Lep 12358	MG522293	BOLD:ACE9765	Tiroler Landesmuseum
Udea uliginosalis	PHLAB221-10	TLMF Lep 01021	HM381585	BOLD:AAC7935	Tiroler Landesmuseum
Udea uliginosalis	LEALT014-16	TLMF Lep 20237	MG191927	BOLD:ADE0060	Tiroler Landesmuseum
Udea uliginosalis	ABOLA947-15	TLMF Lep 16907	MG522799	BOLD:ACE9765	Tiroler Landesmuseum
Udea uliginosalis	PHLAE582-11	TLMF Lep 05087	KX042181	BOLD:AAC7935	Tiroler Landesmuseum
Xanthorhoe decoloraria	LEALT407-16	TLMF Lep 20630	MG522624	BOLD:AAA5318	Tiroler Landesmuseum
Xanthorhoe decoloraria	PHLAA434-09	TLMF Lep 00474	HM425949	BOLD:AAA5318	Tiroler Landesmuseum
Xanthorhoe decoloraria	PHLSA399-11	TLMF Lep 05854	KM572578	BOLD:AAA5318	Tiroler Landesmuseum
Xanthorhoe decoloraria	ABOLA437-14	TLMF Lep 16124	MG522731	BOLD:AAA5318	Tiroler Landesmuseum
Xanthorhoe decoloraria	LEATB775-13	TLMF Lep 10952	MG522108	BOLD:AAA5318	Naturmuseum Suedtirol
Xanthorhoe decoloraria	LEALT365-16	TLMF Lep 20588	MG521987	BOLD:AAA5318	Tiroler Landesmuseum
Xanthorhoe decoloraria	PHLAC732-10	TLMF Lep 02767	KX042678	BOLD:AAA5318	Tiroler Landesmuseum
Xanthorhoe decoloraria	LEFIB853-10	MM02857	HM871730	BOLD:AAA5318	University of Oulu
Xanthorhoe decoloraria	LEFID431-10	MM06361	HQ570327	BOLD:AAA5318	University of Oulu
Xanthorhoe decoloraria	LEALT408-16	TLMF Lep 20631	MG521927	BOLD:AAA5318	Tiroler Landesmuseum
Xanthorhoe decoloraria	LEFIA752-10	MM04106	HM386896	BOLD:AAA5318	University of Oulu
Xanthorhoe decoloraria	PHLAA337-09	TLMF Lep 00377	HM425865	BOLD:AAA5318	Tiroler Landesmuseum
Xanthorhoe decoloraria	LEATC072-13	TLMF Lep 11199	MG522710	BOLD:AAA5318	Tiroler Landesmuseum
Xanthorhoe decoloraria	PHLAA685-09	TLMF Lep 00725	HM426085	BOLD:AAA5318	Tiroler Landesmuseum
Xanthorhoe decoloraria	LEATH552-14	TLMF Lep 15764	MG522454	BOLD:AAA5318	Naturmuseum Suedtirol
Xanthorhoe decoloraria	LEATB782-13	TLMF Lep 10959	MG522127	BOLD:AAA5318	Tiroler Landesmuseum
Xanthorhoe montanata	GBLAA2070-15	BC ZSM Lep 100074	MG522418	BOLD:AAB2524	SNSB, Zoologische Staatssammlung Muenchen
Xanthorhoe montanata	LEATB516-13	TLMF Lep 10693	MG521994	BOLD:AAB2524	Tiroler Landesmuseum
Xanthorhoe montanata	PHLAH808-12	TLMF Lep 08612	MG522585	BOLD:AAB2524	Tiroler Landesmuseum
Xanthorhoe montanata	LEFIK845-10	MM18420	JN279405	BOLD:AAB2524	University of Oulu
Xanthorhoe montanata	GWORE1503-08	BC ZSM Lep 15096	HQ601606	BOLD:AAB2524	SNSB, Zoologische Staatssammlung Muenchen

Xanthorhoe montanata	LEALT437-16	TLMF Lep 20660	MG521960	BOLD:AAB2524	Tiroler Landesmuseum
Xanthorhoe montanata	LEALT387-16	TLMF Lep 20610	MG522719	BOLD:AAB2524	Tiroler Landesmuseum
Xanthorhoe montanata	ABOLB838-15	KLM Lep 03498	MG521944	BOLD:AAB2524	Landesmuseum Kaernten
Xanthorhoe montanata	PHLAB301-10	TLMF Lep 01101	HQ968468	BOLD:AAB2524	Tiroler Landesmuseum
Xanthorhoe montanata	LEATD267-13	TLMF Lep 12914	MG521990	BOLD:AAB2524	Tiroler Landesmuseum
Xanthorhoe montanata	LEALT436-16	TLMF Lep 20659	MG521972	BOLD:AAB2524	Tiroler Landesmuseum
Xanthorhoe montanata	GWORA2956-15	BC ZSM Lep 86034	MG522816	BOLD:AAB2524	SNSB, Zoologische Staatssammlung Muenchen
Xanthorhoe montanata	PHLAB1195-10	TLMF Lep 01995	HQ968405	BOLD:AAB2524	Tiroler Landesmuseum
Xanthorhoe montanata	LEFIE177-10	MM08453	HM873923	BOLD:AAB2524	University of Oulu
Xanthorhoe montanata	LEATC220-13	TLMF Lep 11347	MG522066	BOLD:AAB2524	Tiroler Landesmuseum
Xanthorhoe montanata	LEATB829-13	TLMF Lep 11006	MG522145	BOLD:AAB2524	Tiroler Landesmuseum
Xanthorhoe montanata	LEFIB850-10	MM02846	HM871727	BOLD:AAB2524	University of Oulu
Xanthorhoe montanata	GBLAC120-13	BC ZSM Lep 66861	MG522530	BOLD:AAB2524	SNSB, Zoologische Staatssammlung Muenchen
Xanthorhoe montanata	NLLEA447-12	RMNH.INS.540639	KX048344	BOLD:AAB2524	Naturalis Biodiversity Centre
Xanthorhoe montanata	PHLAB1213-10	TLMF Lep 02013	HQ968423	BOLD:AAB2524	Tiroler Landesmuseum
Xanthorhoe montanata	GWORE1504-08	BC ZSM Lep 15097	HQ601605	BOLD:AAB2524	SNSB, Zoologische Staatssammlung Muenchen
Xanthorhoe montanata	NLLEA257-12	RMNH.INS.538872	KX047920	BOLD:AAB2524	Naturalis Biodiversity Centre
Xanthorhoe montanata	NOLEP061-14	BCZMBNLep0061	MG522077	BOLD:AAB2524	University of Bergen, Natural History Collections
Xanthorhoe montanata	PHLAC404-10	TLMF Lep 02439	JF859980	BOLD:AAB2524	Tiroler Landesmuseum
Xanthorhoe montanata	GBLAC928-13	BC ZSM Lep 77504	MG522198	BOLD:AAB2524	SNSB, Zoologische Staatssammlung Muenchen
Xanthorhoe montanata	PHLAH738-12	TLMF Lep 08557	KM573407	BOLD:AAB2524	inatura, Dornbirn
Xanthorhoe montanata	NLLEA140-12	RMNH.INS.538755	KX049780	BOLD:AAB2524	Naturalis Biodiversity Centre
Xanthorhoe montanata	GWORL351-09	BC ZSM Lep 22063	GU686921	BOLD:AAB2524	SNSB, Zoologische Staatssammlung Muenchen
Xanthorhoe montanata	LEATD315-13	TLMF Lep 12962	MG521931	BOLD:AAB2524	Tiroler Landesmuseum
Xanthorhoe montanata	GBLAC560-13	BC ZSM Lep 78181	MG522565	BOLD:AAB2524	SNSB, Zoologische Staatssammlung Muenchen
Xanthorhoe montanata	LEATB689-13	TLMF Lep 10866	MG522257	BOLD:AAB2524	Naturmuseum Suedtirol
Xestia speciosa	PHLAE212-11	TLMF Lep 04432	JN284165	BOLD:ACF2698	Tiroler Landesmuseum
Xestia speciosa	LEFIB313-10	MM00840	HM871213	BOLD:ACE4666	University of Oulu
Xestia speciosa	LEFIH165-10	MM15133	HM876785	BOLD:ACE4666	University of Oulu
Xestia speciosa	LEFIL463-10	MM18761	JF854609	BOLD:ACE4666	University of Oulu

Xestia speciosa	LEALT432-16	TLMF Lep 20655	MG522004	BOLD:ACE4665	Tiroler Landesmuseum
Xestia speciosa	PHLAA129-09	TLMF Lep 00169	GU689157	BOLD:ACF2698	Tiroler Landesmuseum
Xestia speciosa	PHLAA142-09	TLMF Lep 00182	GU689197	BOLD:ACF2698	Tiroler Landesmuseum
Xestia speciosa	LEFIE017-10	MM08072	HM873766	BOLD:ACE4666	University of Oulu
Xestia speciosa	LEFIH169-10	MM15137	HM876789	BOLD:ACE4666	University of Oulu
Xestia speciosa	FBLMV029-09	BC ZSM Lep 28009	GU707438	BOLD:ACF2698	SNSB, Zoologische Staatssammlung Muenchen
Xestia speciosa	LEFIH163-10	MM15114	HM876783	BOLD:ACE4666	University of Oulu
Xestia speciosa	PHLAE211-11	TLMF Lep 04431	JN284164	BOLD:ACF2698	Tiroler Landesmuseum
Xestia speciosa	LEFIJ1454-12	MM22722	KX048781	BOLD:ACE4666	University of Oulu
Xestia speciosa	LEFIL462-10	MM18760	JF854608	BOLD:ACE4666	University of Oulu
Xestia speciosa	LEFIH167-10	MM15135	HM876787	BOLD:ACE4666	University of Oulu
Xestia speciosa	PHLAA276-09	TLMF Lep 00316	HM425811	BOLD:ACF2698	Tiroler Landesmuseum
Xestia speciosa	LEFIE018-10	MM08073	HM873767	BOLD:ACE4666	University of Oulu
Xestia speciosa	PHLAA130-09	TLMF Lep 00170	GU689158	BOLD:ACF2698	Tiroler Landesmuseum
Xestia speciosa	LEATB798-13	TLMF Lep 10975	MG522802	BOLD:ACF2698	Tiroler Landesmuseum
Xestia speciosa	LEFIL464-10	MM18762	JF854610	BOLD:ACE4666	University of Oulu
Xestia speciosa	LEFIH171-10	MM15139	HM876791	BOLD:ACE4666	University of Oulu
Xestia speciosa	LEFIH162-10	MM15113	HM876782	BOLD:ACE4666	University of Oulu
Xestia speciosa	PHLAH669-12	TLMF Lep 08488	KM572274	BOLD:ACF2698	inatura, Dornbirn
Xestia speciosa	LEFIH170-10	MM15138	HM876790	BOLD:ACE4666	University of Oulu
Xestia speciosa	LEALT431-16	TLMF Lep 20654	MG522811	BOLD:ACE4665	Tiroler Landesmuseum
Xestia speciosa	LEFIB809-10	MM02739	HM871686	BOLD:ACE4666	University of Oulu
Xestia speciosa	DEEUR566-15	TLMF Lep 17761	MG522232	BOLD:ACF2698	Research Collection of Peter Buchner
Xestia speciosa	PHLAA143-09	TLMF Lep 00183	GU689151	BOLD:ACF2698	Tiroler Landesmuseum
Xestia speciosa	PHLAD678-11	TLMF Lep 04043	JN284160	BOLD:ACF2698	Tiroler Landesmuseum
Xestia speciosa	LEFIH168-10	MM15136	HM876788	BOLD:ACE4666	University of Oulu
Xestia speciosa	GBLAC932-13	BC ZSM Lep 77508	MG522630	BOLD:ACF2698	SNSB, Zoologische Staatssammlung Muenchen
Xestia speciosa	PHLAC935-10	TLMF Lep 02970	JF860403	BOLD:ACF2698	Tiroler Landesmuseum
Xestia speciosa	PHLAA210-09	TLMF Lep 00250	HM425759	BOLD:ACF2698	Tiroler Landesmuseum
Xestia speciosa	PHLAC423-10	TLMF Lep 02458	JF859999	BOLD:ACF2698	Tiroler Landesmuseum

Xestia speciosa	LEFID249-10	MM06113	HM873048	BOLD:ACE4666	University of Oulu
Xestia speciosa	LEFIH164-10	MM15132	HM876784	BOLD:ACE4666	University of Oulu
Xestia speciosa	LEFIH166-10	MM15134	HM876786	BOLD:ACE4666	University of Oulu
Xestia speciosa	PHLAC934-10	TLMF Lep 02969	JF860402	BOLD:ACF2698	Tiroler Landesmuseum
Xestia speciosa	LEALT360-16	TLMF Lep 20583	MG522397	BOLD:ACE4665	Tiroler Landesmuseum
Yponomeuta evonymella	LEATA469-13	TLMF Lep 10076	MG522655	BOLD:AAA7740	Tiroler Landesmuseum
Yponomeuta evonymella	LEATE828-13	TLMF Lep 12240	MG522692	BOLD:AAA7740	Tiroler Landesmuseum
Yponomeuta evonymella	LEFIC907-10	MM05233	HM872724	BOLD:AAA7740	University of Oulu
Yponomeuta evonymella	LEFIE040-10	MM08126	HM873789	BOLD:AAA7740	University of Oulu
Yponomeuta evonymella	LEFIB039-10	MM00307	HM870948	BOLD:AAA7740	University of Oulu
Yponomeuta evonymella	PHLAC181-10	TLMF Lep 02216	JF859798	BOLD:AAA7740	Tiroler Landesmuseum
Yponomeuta evonymella	LEALT085-16	TLMF Lep 20308	MG522307	BOLD:AAA7740	Tiroler Landesmuseum
Yponomeuta evonymella	PHLAV270-12	TLMF Lep 08089	KM573230	BOLD:AAA7740	inatura, Dornbirn
Yponomeuta evonymella	LEATB005-13	TLMF Lep 10182	MG522657	BOLD:AAA7740	Tiroler Landesmuseum
Yponomeuta evonymella	PHLAG591-12	TLMF Lep 07270	MG522388	BOLD:AAA7740	Tiroler Landesmuseum
Yponomeuta evonymella	LEFIF927-10	MM13368	HM875609	BOLD:AAA7740	University of Oulu
Yponomeuta evonymella	PHLAB082-10	TLMF Lep 00882	HQ968256	BOLD:AAA7740	Tiroler Landesmuseum
Yponomeuta evonymella	LEFIB550-10	MM02179	HM871433	BOLD:AAA7740	University of Oulu
Yponomeuta evonymella	LEALT086-16	TLMF Lep 20309	MG522612	BOLD:AAA7740	Tiroler Landesmuseum
Ypsolopha dentella	LEATE585-13	TLMF Lep 11997	MG522436	BOLD:AAB2626	Naturmuseum Suedtirol
Ypsolopha dentella	PHLAI783-13	TLMF Lep 09345	MG522053	BOLD:AAB2626	Tiroler Landesmuseum
Ypsolopha dentella	LEALT190-16	TLMF Lep 20413	MG521919	BOLD:AAB2626	Tiroler Landesmuseum
Ypsolopha dentella	LEFIF931-10	MM13373	HM875613	BOLD:AAB2626	University of Oulu
Ypsolopha dentella	PHLAG759-12	TLMF Lep 07438	MG522729	BOLD:AAB2626	Tiroler Landesmuseum
Ypsolopha dentella	LEALT197-16	TLMF Lep 20420	MG522478	BOLD:AAB2626	Tiroler Landesmuseum
Ypsolopha dentella	LEALT189-16	TLMF Lep 20412	MG522208	BOLD:AAB2626	Tiroler Landesmuseum
Ypsolopha dentella	LEATE586-13	TLMF Lep 11998	MG522656	BOLD:AAB2626	Tiroler Landesmuseum
Ypsolopha dentella	LEFIE382-10	MM08913	HM874106	BOLD:AAB2626	University of Oulu
Ypsolopha dentella	LEFIB552-10	MM02184	HM871435	BOLD:AAB2626	University of Oulu
Ypsolopha nemorella	LEALT023-16	TLMF Lep 20246	MG522562	BOLD:AAE4873	Tiroler Landesmuseum

Ypsolopha nemorella	PHLAH586-12	TLMF Lep 08405	KM572237	BOLD:AAE4873	inatura, Dornbirn
Ypsolopha nemorella	LEFID476-10	MM06418	HM873242	BOLD:AAE4873	University of Oulu
Ypsolopha nemorella	LEATE672-13	TLMF Lep 12084	MG521982	BOLD:AAE4873	Tiroler Landesmuseum
Ypsolopha nemorella	LEATF405-14	TLMF Lep 13717	MG522480	BOLD:AAE4873	Tiroler Landesmuseum
Ypsolopha nemorella	LEATJ1018-15	TLMF Lep 18878	MG522117	BOLD:AAE4873	Tiroler Landesmuseum
Ypsolopha nemorella	LEALT022-16	TLMF Lep 20245	MG522153	BOLD:AAE4873	Tiroler Landesmuseum
Ypsolopha nemorella	LEATE673-13	TLMF Lep 12085	MG522006	BOLD:AAE4873	Tiroler Landesmuseum
Ypsolopha nemorella	PHLAG160-12	TLMF Lep 06470	MG522417	BOLD:AAE4873	Tiroler Landesmuseum
Ypsolopha nemorella	LEFID474-10	MM06416	HQ963160	BOLD:AAE4873	University of Oulu
Ypsolopha nemorella	LEFIB553-10	MM02185	HM871436	BOLD:AAE4873	University of Oulu
Ypsolopha nemorella	LEALT024-16	TLMF Lep 20247	MG522024	BOLD:AAE4873	Tiroler Landesmuseum
Zeiraphera griseana	LEFIC561-10	MM04338	HM872382	BOLD:AAA7907	University of Oulu
Zeiraphera griseana	LEFIE888-10	MM10166	HM874606	BOLD:AAA7907	University of Oulu
Zeiraphera griseana	LEFIE376-10	MM08885	HM874100	BOLD:AAA7907	University of Oulu
Zeiraphera griseana	LEALT058-16	TLMF Lep 20281	MG522662	BOLD:AAA7907	Tiroler Landesmuseum
Zeiraphera griseana	LEFIG857-10	MM15721	HM876505	BOLD:AAA7907	University of Oulu
Zeiraphera griseana	LEFIE881-10	MM10158	HM874599	BOLD:AAA7907	University of Oulu
Zeiraphera griseana	LEFIE086-10	MM08259	HM873833	BOLD:AAA7907	University of Oulu
Zeiraphera griseana	LEATF398-14	TLMF Lep 13710	MG522151	BOLD:AAA7907	Tiroler Landesmuseum
Zeiraphera griseana	LEFIG856-10	MM15720	HM876504	BOLD:AAA7907	University of Oulu
Zeiraphera griseana	LEFIE882-10	MM10159	HM874600	BOLD:AAA7907	University of Oulu
Zeiraphera griseana	PHLAC288-10	TLMF Lep 02323	JN820128	BOLD:AAA7907	Tiroler Landesmuseum
Zeiraphera griseana	LEATC465-13	TLMF Lep 12447	MG522509	BOLD:AAA7907	Tiroler Landesmuseum
Zeiraphera griseana	LEFIG858-10	MM15722	HM876506	BOLD:AAA7907	University of Oulu
Zeiraphera griseana	LEFIE377-10	MM08886	HM874101	BOLD:AAA7907	University of Oulu
Zeiraphera griseana	LEFIE375-10	MM08884	HM874099	BOLD:AAA7907	University of Oulu
Zeiraphera griseana	LEFIE083-10	MM08249	HM873830	BOLD:AAA7907	University of Oulu
Zeiraphera griseana	LEFIE378-10	MM08887	HM874102	BOLD:AAA7907	University of Oulu
Zeiraphera griseana	LEALT079-16	TLMF Lep 20302	MG522317	BOLD:AAA7907	Tiroler Landesmuseum
Zeiraphera griseana	LEFIE883-10	MM10160	HM874601	BOLD:AAA7907	University of Oulu

Zeiraphera griseana	LEATB948-13	TLMF Lep 11125	MG522310	BOLD:AAA7907	Tiroler Landesmuseum
Zeiraphera griseana	LEATB945-13	TLMF Lep 11122	MG522635	BOLD:AAA7907	Tiroler Landesmuseum
Zeiraphera griseana	LEFIE049-10	MM08156	HM873797	BOLD:AAA7907	University of Oulu
Zeiraphera griseana	LEALT101-16	TLMF Lep 20324	MG522505	BOLD:AAA7907	Tiroler Landesmuseum
